# Membrane contact sites between chloroplasts and the pathogen interface underpin plant focal immune responses

**DOI:** 10.1093/plcell/koaf214

**Published:** 2025-09-05

**Authors:** Enoch Lok Him Yuen, Zachary Savage, Vojtěch Pražák, Zhongyuan Liu, Vanda Adamkova, Freddie King, Cristina Vuolo, Tarhan Ibrahim, Yijun Wang, Saskia Jenkins, Yuanyang Zhou, Yasin Tumtas, Jessica Lee Erickson, Jennifer Prautsch, Andrada I Balmez, Johannes Stuttmann, Cian Duggan, Francesco Rivetti, Camilla Molinari, David C A Gaboriau, Philip Carella, Xiaohong Zhuang, Martin Schattat, Tolga O Bozkurt

**Affiliations:** Department of Life Sciences, Imperial College London, London SW7 2AZ, UK; Department of Life Sciences, Imperial College London, London SW7 2AZ, UK; Leibniz-Institut für Virologie (LIV), Hamburg 20251, Germany; Centre for Structural Systems Biology, Hamburg 22607, Germany; Department of Biochemistry, University of Oxford, Oxford OX1 3QU, UK; Centre for Cell & Developmental Biology and State Key Laboratory of Agrobiotechnology, School of Life Sciences, Chinese University of Hong Kong, Hong Kong, China; Department of Life Sciences, Imperial College London, London SW7 2AZ, UK; Department of Life Sciences, Imperial College London, London SW7 2AZ, UK; Department of Life Sciences, Imperial College London, London SW7 2AZ, UK; Department of Life Sciences, Imperial College London, London SW7 2AZ, UK; Department of Life Sciences, Imperial College London, London SW7 2AZ, UK; Department of Life Sciences, Imperial College London, London SW7 2AZ, UK; Department of Life Sciences, Imperial College London, London SW7 2AZ, UK; Department of Life Sciences, Imperial College London, London SW7 2AZ, UK; Department of Biochemistry of Plant Interactions, Leibniz Institute for Plant Biochemistry, Halle 06120, Germany; Department of Plant Physiology, Martin-Luther-University Halle-Wittenberg, Halle 06120, Germany; Department of Life Sciences, Imperial College London, London SW7 2AZ, UK; Department of Plant Genetics, Martin-Luther-University Halle-Wittenberg, Halle 06120, Germany; CEA, CNRS, BIAM, UMR7265, LEMiRE (Microbial Ecology of the Rhizosphere), Aix Marseille University, Saint‑Paul lez Durance 13115, France; Department of Life Sciences, Imperial College London, London SW7 2AZ, UK; Department of Life Sciences, Imperial College London, London SW7 2AZ, UK; Department of Life Sciences, Imperial College London, London SW7 2AZ, UK; Facility for Imaging by Light Microscopy, NHLI, Imperial College London, London SW7 2AZ, UK; Cell and Developmental Biology, John Innes Centre, Norwich NR4 7UH, UK; Centre for Cell & Developmental Biology and State Key Laboratory of Agrobiotechnology, School of Life Sciences, Chinese University of Hong Kong, Hong Kong, China; Department of Plant Physiology, Martin-Luther-University Halle-Wittenberg, Halle 06120, Germany; Department of Life Sciences, Imperial College London, London SW7 2AZ, UK

## Abstract

Communication between cellular organelles is essential for mounting effective innate immune responses. The transport of organelles to pathogen penetration sites and their assembly around the host membrane, which delineates the plant–pathogen interface, are well documented. However, whether organelles associate with these specialized interfaces, and the extent to which this process contributes to immunity, remain unknown. Here, we discovered defense-related membrane contact sites (MCS) comprising a membrane tethering complex between chloroplasts and the extrahaustorial membrane (EHM) surrounding the haustorium of the pathogen *Phytophthora infestans* in *Nicotiana benthamiana*. The assembly of this complex involves association between the chloroplast outer envelope protein CHLOROPLAST UNUSUAL POSITIONING 1 (CHUP1) and its plasma membrane-associated partner KINESIN-LIKE PROTEIN FOR ACTIN-BASED CHLOROPLAST MOVEMENT 1 (KAC1). Our biochemical assays revealed that CHUP1 and KAC1 interact, and infection cell biology assays demonstrated their co-accumulation in foci where chloroplasts contact the EHM. Genetic depletion of CHUP1 or KAC1 reduces the focal deposition of callose around the haustorium without affecting other core immune processes. Our findings suggest that the chloroplast–EHM attachment complex promotes plant focal immunity, revealing key components and their potential roles in the deposition of defense materials at the pathogen interface. These results advance our understanding of organelle-mediated immunity and highlight the significance of MCS in plant–pathogen interactions.

## Teaser

Chloroplast tethering to the pathogen interface via membrane contact sites promotes focal callose deposition and strengthens plant immunity.

## Introduction

Filamentous pathogens such as oomycetes and fungi intimately interact with plant hosts, often through specialized infection structures that penetrate the host cells. In response, the invaded plant cell activates a cell-autonomous defense mechanism interface known as focal immunity (Kwon et al. 2008; Bozkurt et al. 2011; Yuen et al. 2023, 2024a). This defense involves significant cellular reorganization, including organelle relocation, cell-wall reinforcements at pathogen contact sites via callose deposition, and the polarized secretion of antimicrobials (Heath et al. 1997; Bozkurt and Kamoun 2020; Savage et al. 2021; Yuen et al. 2024a). In addition to the secretory system and the nucleus, organelles such as chloroplasts, mitochondria and peroxisomes accumulate around host cell penetration sites of fungal and oomycete pathogens (Fuchs et al. 2016; Bozkurt and Kamoun 2020; Savage et al. 2021). However, the physical association of organelles with the plant–pathogen interface and the extent to which these processes contribute to immunity remain unclear.

Cellular homeostasis relies on efficient interorganelle communication, a process facilitated by long-range vesicle trafficking and short-range membrane contact sites (MCS). MCS are specialized regions where organelles come into close proximity, allowing for the direct transfer of lipids, proteins, signaling molecules, and metabolites. These sites serve as critical gateways for rapid and effective intracellular communication, enabling cells to quickly adapt and respond to stress conditions (Chung et al. 2015; Pérez-Sancho et al. 2016; Prinz et al. 2020). Recent research has highlighted the emerging roles of MCS in mammalian innate immunity (Hubber et al. 2014). Moreover, pathogens have developed strategies to target and subvert MCS, perturbing interorganelle communications to undermine host immune defenses and exploit cellular resources (Agaisse and Derré 2014; Vormittag et al. 2023). However, the involvement of MCS in plant–pathogen interactions remains unexplored. Additionally, the identity and roles of protein-tethering complexes that regulate the functions of MCS in plants are still largely elusive (Pérez-Sancho et al. 2016). Understanding these mechanisms could reveal insights into how organelle communication contributes to immune responses.

Accumulating evidence points to key roles of chloroplasts in the deployment of various plant immune responses (Nomura et al. 2012; Littlejohn et al. 2021). Upon immune activation and signaling mediated through mitogen-activated protein kinases (MAPKs), chloroplasts terminate photosynthesis and activate a range of defense responses such as the production of reactive oxygen species (ROS) and defense hormones (Su et al. 2018). During the immune response, chloroplasts alter their morphology by extending stroma-filled tubules, called stromules, that can make contacts with other membranes and organelles such as the nucleus (Caplan et al. 2015; Savage et al. 2021; Jung et al. 2024). In addition, chloroplasts cluster around the nucleus during immune stimulation (Ding et al. 2019), a response that is presumed to contribute toward plant defense, possibly through facilitating more efficient chloroplast-to-nucleus signaling.

In line with these immune functions, an increasing number of pathogen effectors have been identified that target chloroplast processes (de Torres Zabala et al. 2015; Petre et al. 2016; Xu et al. 2019). Plants, in turn, monitor such effector activities using nucleotide-binding leucine-rich repeat (NLR) immune receptors (Shepherd et al. 2023; Contreras et al. 2023a, 2023b; Ibrahim et al. 2024; Selvaraj et al. 2024). For instance, effectors from a diverse range of pathogens target chloroplasts to interfere with immune signaling, photosynthetic activity, stromule formation, and salicylic acid biosynthesis, reinforcing the emerging role of chloroplasts in plant immunity (Jelenska et al. 2007, 2010; Rodriguez-Herva et al. 2012; Gao et al. 2020; Medina-Puche et al. 2020; Savage et al. 2021).

The Irish potato famine pathogen, *Phytophthora infestans*, can penetrate host cells via specialized infection structures called haustoria, which mediate the delivery of effector proteins inside the host cells. Haustoria are excluded from the host cytoplasm through a newly synthesized plant-derived membrane called the extrahaustorial membrane (EHM) (Whisson et al. 2007; Wang et al. 2018; King et al. 2024). Remarkably, chloroplasts frequently gather around *P. infestans* haustoria and appear to establish tight contacts with the EHM (Savage et al. 2021). Given the range of antimicrobial and defense components produced by chloroplasts, their positioning at the pathogen interface likely contributes to focal plant immune responses. The mechanisms underlying chloroplast photorelocation in response to light intensity, involving membrane attachment, detachment, and movement, are relatively well understood, with key components such as CHLOROPLAST UNUSUAL POSITIONING 1 (CHUP1) and KINESIN-LIKE PROTEIN FOR ACTIN-BASED CHLOROPLAST MOVEMENT 1 (KAC1) identified (Oikawa et al. 2003, 2008; Suetsugu et al. 2010, 2012). Both CHUP1 and KAC1 are involved in the photorelocation movement of chloroplasts and the association of chloroplasts with the plasma membrane (PM) by regulating short actin filaments on the chloroplast envelope (cp-actin filaments) (Oikawa et al. 2003, 2008; Kadota et al. 2009; Suetsugu et al. 2010). However, the extent to which chloroplast movement and positioning around the haustoria contributes to plant immunity remains to be elucidated.

In this study, we investigated the role of the chloroplast movement and membrane anchoring protein CHUP1 in plant immunity against *P. infestans*. Using virus-induced gene silencing (VIGS) and CRISPR knockouts, we found that CHUP1-deficient *Nicotiana benthamiana* plants exhibit significantly increased pathogen growth, indicating that CHUP1 positively contributes to immunity. Despite no significant changes in chloroplast movement toward haustoria or other core immune processes such as MAPK-triggered signaling and hypersensitive response (HR) cell death, CHUP1-knockout plants showed reduced callose deposition at haustoria penetration sites, highlighting the involvement of CHUP1 in focal immune responses. We discovered that CHUP1 interacts with the kinesin-like protein KAC1, and both proteins co-accumulate at chloroplast-EHM MCS. This suggests their cooperative role in anchoring chloroplasts to the pathogen interface and enhancing immunity. Consistently, KAC1 accumulated at chloroplast-EHM MCS in a CHUP1-dependent manner, and depletion of KAC1 led to reduced callose deposition around the haustorium and enhanced disease susceptibility. These findings underscore the importance of CHUP1 and KAC1 cooperation in coordinating the tethering of chloroplasts to the pathogen interface and mounting effective immune responses via MCS.

## Results

### CHUP1 positively contributes to immunity against *Phytophthora infestans*

**Figure 1.**
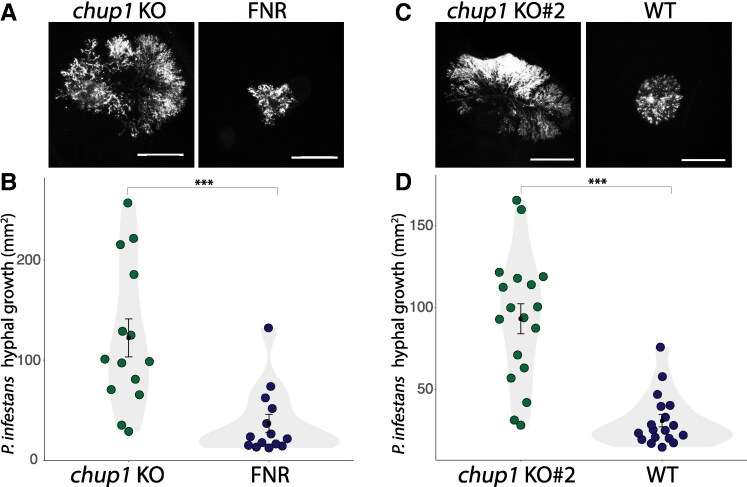
CHUP1 positively contributes to immunity against *P. infestans*. **A, B)**  *chup1* KO plants increase hyphal growth of *P. infestans* compared to control FNR plants. **A)** Four-wk-old *chup1* KO and FNR leaves were infected with tdTomato-expressing *P. infestans*, and pathogen growth was calculated by measuring hyphal growth using fluorescence stereomicroscope at 5 d post-inoculation. Scale bars represent 5 mm. **B)** Violin plot illustrating that *chup1* KO plants (122.1 mm^2^, *n* = 83 infection spots) display a significant increase in *P. infestans* hyphal growth compared to control FNR plants (36.9 mm^2^, *n* = 83 infection spots). **C, D)**  *chup1* KO#2 plants increase hyphal growth of *P. infestans* compared to control WT plants. **C)** Four-wk-old *chup1* KO#2 and WT leaves were infected with tdTomato-expressing *P. infestans*, and pathogen growth was calculated by measuring hyphal growth using fluorescence stereomicroscope at 5 d post-inoculation. Scale bars represent 5 mm. **D)** Violin plot illustrating that *chup1* KO#2 plants (93.1 mm^2^, *n* = 97 infection spots) exhibit a significant increase in *P. infestans* hyphal growth compared to control FNR plants (30.7 mm^2^, *n* = 94 infection spots). Each dot represents the average of all infection spots on the same leaf, with each leaf having between three and six infection spots. Statistical differences were analyzed by Mann–Whitney U test in R. Error bars represent the mean ± standard error of the mean (SE). Differences in measurements were considered highly significant when *P* < 0.001 (***). KO, knockout; WT, wild type.

The role of chloroplasts in providing biochemical defense against pathogens is well established (Nomura et al. 2012; Caplan et al. 2015; Littlejohn et al. 2021). An emerging yet less understood aspect of chloroplast immunity involves the positioning and movement of epidermal chloroplasts during infection and their contribution to the immune response (Ding et al. 2019; Irieda and Takano 2021). We previously demonstrated that during infection by the oomycete pathogen *P. infestans*, epidermal chloroplasts in the model solanaceous plant *N. benthamiana* accumulate around the haustoria (Savage et al. 2021). To investigate this phenomenon further, we performed infection assays on *N. benthamiana* following the downregulation of the chloroplast movement and anchoring gene, *CHUP1*. Silencing the two identified *CHUP1* alleles (*NbCHUP1a* and *NbCHUP1b*) in transgenic *N. benthamiana* expressing green fluorescent protein (GFP) in the chloroplast stroma (CP plants) via VIGS (Supplementary Fig. S1A) resulted in significantly higher levels of hyphal growth of tdTomato-expressing *P. infestans* compared to the silencing control (Supplementary Fig. S1, B and C). To further validate these findings, we generated CRISPR-knockout lines lacking *NbCHUP1a* and *NbCHUP1b* (referred to as *chup1* KO plants) from a transgenic parental line of *N. benthamiana* expressing the chimeric protein ferredoxin NADP^+^ oxidoreductase (FNR):eGFP, which targets the plastid stroma (referred to as FNR plants) (Supplementary Fig. S1D). *chup1* KO plants did not exhibit any major developmental defects compared to FNR control plants (Supplementary Fig. S1E). Following infection with *P. infestans*, the mean hyphal growth of the pathogen was approximately 3.3-fold higher in *chup1* KO plants compared to FNR control plants (Fig. 1, A and B), indicating that *chup1* KO plants are significantly more susceptible than the FNR control plants. Additionally, we generated independent *chup1* knockout lines from wild-type (WT) *N. benthamiana* plants (Supplementary Fig. S1F) (referred to as *chup1* KO#2 plants). These *chup1* KO#2 plants also demonstrated significantly higher *P. infestans* hyphal growth compared to the WT control plants (Fig. 1, C and D). These results implicate that the chloroplast outer envelope protein CHUP1 contributes to plant defense against adapted pathogens.

CHUP1 is known to facilitate chloroplast movement and photorelocation through cp-actin polymerization (Oikawa et al. 2003; Schmidt von Braun and Schleiff 2008; Wada and Kong 2018). Therefore, we hypothesized that increased susceptibility in *chup1* KO plants could be due to perturbations in chloroplast movement and positioning around the haustoria. To investigate this, we imaged infected *N. benthamiana* epidermal cells and quantified chloroplast–haustoria associations in *chup1* KO and FNR plants. Our quantitative analysis showed no significant difference in the number of haustoria that is associated with chloroplasts between *chup1* KO and FNR plants (Supplementary Fig. S1, G and H), indicating that enhanced susceptibility upon loss of CHUP1 is not due to impaired chloroplast movement toward the haustoria.

Chloroplasts clustering around nucleus is regarded to be a general plant immune response upon pathogen recognition and immune activation (Caplan et al. 2015; Ding et al. 2019). Therefore, we next checked whether chloroplast accumulation around the haustoria is impaired in *chup1* KO plants. Intriguingly, microscopy analysis revealed that chloroplasts tend to accumulate more around the nucleus in *chup1* KO plants compared to FNR plants (Supplementary Fig. S1, I and J), indicating that the enhanced susceptibility of *chup1* KO plants is not due to impaired perinuclear clustering of chloroplasts. Lastly, we revealed that the number of chloroplasts in the epidermal cells and the total chlorophyll concentration in leaves did not vary significantly between *chup1* KO and FNR plants (Supplementary Fig. S1, K to M; Videos 1 and 2). Altogether, these results indicate that CHUP1 is not essential for chloroplast positioning around haustoria, and the increased disease susceptibility in CHUP1 knockouts is not due to impaired chloroplast positioning or abnormal chloroplast numbers.

### CHUP1 accumulates at the chloroplast–PM and chloroplast–EHM contact sites

**Figure 2.**
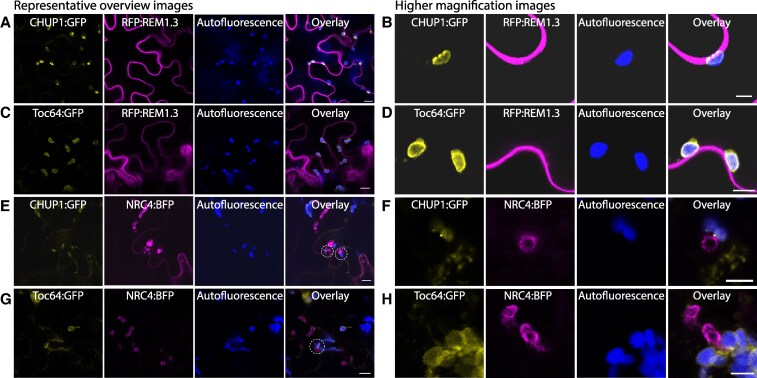
CHUP1 forms punctate accumulation at chloroplast–PM and chloroplast–EHM MCS. **A, B)** CHUP1 forms punctate accumulation at chloroplast–PM MCS (83.8%, *n* = 68 chloroplasts). Confocal micrographs of *N. benthamiana* leaf epidermal cells transiently expressing CHUP1:GFP with RFP:REM1.3. The leaves were not infected. **C, D)** The control Toc64 does not form punctate accumulation at chloroplast–PM MCS (0.0%, *n* = 59 chloroplasts). Confocal micrographs of *N. benthamiana* leaf epidermal cells transiently expressing Toc64:GFP with RFP:REM1.3. The leaves were not infected. **E, F)** CHUP1 forms punctate accumulation at chloroplast-EHM MCS (61.4%, *n* = 70 chloroplasts). Confocal micrographs of *N. benthamiana* leaf epidermal cells transiently expressing CHUP1:GFP with NRC4:BFP. The leaves were infected with WT *P. infestans* spores at 6 hpi. **G, H)** The control Toc64 does not form punctate accumulation at chloroplast–EHM MCS (0.0%, *n* = 49 chloroplasts). Confocal micrographs of *N. benthamiana* leaf epidermal cells transiently expressing Toc64:GFP with NRC4:BFP. The leaves were infected with WT *P. infestans* spores at 6 hpi. All images were taken at 3 dpi. REM1.3 is used as a PM marker. NRC4 is used as an EHM marker. Presented images are single plane images. Scale bars represent 10 *µ*m in representative overview images, and represent 5 *µ*m in higher magnification images.

To build on our findings of enhanced susceptibility in *chup1* KO plants, we next explored the cell biology of CHUP1 to better understand its role. We generated a C-terminal GFP fusion of CHUP1 (CHUP1:GFP) and examined its cellular localization using confocal microscopy. By co-expressing CHUP1:GFP with the PM marker REMORIN1.3 (REM1.3) tagged with red fluorescent protein (RFP), we observed that CHUP1:GFP accumulates at the chloroplast–PM MCS, forming puncta at this interface (Fig. 2, A and B). Notably, 83.8% of the chloroplasts, positioned in close proximity to the PM, exhibited CHUP1 punctate accumulation facing the PM interface. To validate this observation, we used Toc64:GFP as a control since both CHUP1 and Toc64 are chloroplast outer membrane proteins (Sohrt and Soll 2000). Unlike CHUP1:GFP, Toc64:GFP uniformly labeled the chloroplast outer membrane without forming punctate accumulations at chloroplast–PM MCS (Fig. 2, C and D). This contrast highlights the specific punctate localization pattern of CHUP1 at chloroplast–PM MCS.

Compared to the PM, the EHM has different lipid and protein contents, with most typical membrane proteins excluded from it (Koh et al. 2005; Micali et al. 2011; Bozkurt and Kamoun 2020). Therefore, we next examined the localization of CHUP1, focusing on potential chloroplast–EHM MCS, in the context of *P. infestans* infection. Our live cell imaging of infected plant cells revealed that CHUP1:GFP also accumulates at foci where chloroplasts make contacts with the EHM (Fig. 2, E and F and Supplementary Fig. S2A). Among chloroplasts proximal to the EHM, 61.4% exhibited CHUP1 punctate accumulation facing the EHM interface. In contrast, Toc64:GFP, used as a control, continued to show uniform labeling of the chloroplast outer membrane without any punctate accumulations at chloroplast–EHM MCS (Fig. 2, G and H and Supplementary Fig. S2B). These observations reveal the distinctive accumulation of CHUP1 at MCS, both at chloroplast–PM and chloroplast–EHM, pointing to a role in chloroplast attachment to the pathogen interface.

### CHUP1 contributes to callose deposition at haustorium penetration sites

**Figure 3.**
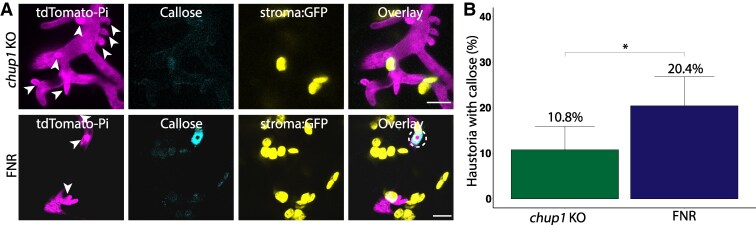
CHUP1 contributes to callose deposition at haustorium penetration sites. **A)** Confocal micrographs of *N. benthamiana* leaf epidermal cells of *chup1* KO plants and control FNR plants. Four-wk-old leaves were infected with *P. infestans* expressing tdTomato, and stained with aniline blue to visualize callose at 3 dpi. Images shown are maximum projection of z-stack images. Chloroplast stroma is visualized via GFP in *chup1* KO and FNR plants. White arrows indicate haustoria, and dashed circles highlight haustoria surrounded by callose deposits. Scale bars represent 10 *μ*m. **B)** Bar graphs showing *chup1* KO plants (10.8%, *n* = 130 haustoria) significantly reduce the frequency of callose deposition around haustoria compared to control FNR plants (20.4%, *N* = 142 haustoria). Statistical differences were analyzed by Fisher's exact test in R. Error bars represent 95% confidence intervals for the proportion of haustoria with callose deposition. Differences in measurements were considered significant when *P* < 0.05 (*). KO, knockout; Pi, *Phytophthora infestans*.

Recognizing that the increased disease susceptibility in *chup1* KO plants is not due to impaired chloroplast positioning around haustoria or abnormal chloroplast numbers (Supplementary Fig. S1, G, H, and K to M), and given that CHUP1 appears to anchor chloroplasts to the EHM (Fig. 2C), we investigated whether focal plant immune responses towards the pathogen are altered in *chup1* KO plants. Accumulation of beta-glucan callose at haustoria penetration sites is a well-established focal immune response typically observed during plant invasion by fungal and oomycete pathogens (Micali et al. 2011; Bozkurt and Kamoun 2020; Yuen et al. 2024a). To determine if callose deposition at the haustorium neck is affected in the absence of CHUP1, we employed aniline blue staining to visualize callose deposition around *P. infestans* haustorium in *chup1* KO and FNR plants (Fig. 3A). Consistent with previous findings on accumulation of callose at the neckband of *P. infestans* haustoria (Bozkurt et al. 2014), approximately 20.4% of haustoria in infected FNR plants exhibited focal callose deposits (Fig. 3B). Notably, we observed a 47.1% reduction in the number of haustoria with a callose neckband band in *chup1* KO plants, with only 10.8% of haustoria showing callose staining (Fig. 3B). These results indicate that CHUP1 is implicated in focal callose deposition at the haustoria of *P. infestans*.

We then investigated whether other core immune pathways were disrupted in *chup1* KO plants. To determine if basal immune responses following pathogen-associated molecular pattern (PAMP) recognition are altered in the absence of CHUP1, we measured MAPK phosphorylation after infiltrating leaves with *P. infestans* (Pi) extract (Yuen et al. 2024a), which serves as a PAMP. Both *chup1* KO and FNR control plants showed a comparable increase in MAPK phosphorylation 24 h post-PAMP treatment (Supplementary Fig. S3A). Next, we examined whether late-stage immune responses, such as defense-related cell death activation, were impaired in *chup1* KO plants. Following the expression of an autoactive variant of a MEK2-like NbMAPKK (MEK2^DD^) (Yang et al. 2001), both *chup1* KO and FNR plants displayed a full tissue necrosis phenotype compared to the EV control (Supplementary Fig. S3, B and C). We also tested whether effector-triggered immunity was impaired in the absence of CHUP1. To assess this, we co-expressed three cell death elicitors with their cognate NLR receptors in plants: *P. infestans* AVR3a with receptor R3a (Bos et al. 2006), *P. infestans* effector AVRblb2 with receptor Rpi-blb2 (Oh et al. 2009), and potato virus X coat protein (PVX-CP) with receptor Rx (Bendahmane et al. 1995). Our cell death analysis showed that both *chup1* KO and FNR plants induced cell death to similar degrees in responses to AVR3a and R3a (Supplementary Fig. S3, D and E), AVRblb2 and Rpi-blb2 (Supplementary Fig. S3, F and G), and PVX-CP and Rx (Supplementary Fig. S3, H and I). Altogether, these findings demonstrate that the activation of PAMP- or effector-triggered immunity is not impaired in the absence of CHUP1. We conclude that the enhanced susceptibility of *chup1* KO plants is most likely due to perturbations of MCS between chloroplasts and the EHM, leading to impaired focal immune responses as indicated by the reduced focal deployment of callose at the haustorium interface.

### KACs contribute to plant immunity against *P. infestans* and callose deposition around the haustoria

The kinesin-like proteins KACs have genetically overlapping and independent functions with CHUP1 in regulating chloroplast photorelocation (Suetsugu et al. 2012, 2016). Along with CHUP1, KACs are indispensable for the polymerization and maintenance of cp-actin filaments (Suetsugu et al. 2010, 2012; Shen et al. 2015). Through cp-actin filaments, these proteins are essential for both the proper movement of chloroplasts and their association with the PM (Suetsugu et al. 2012). While they coordinately mediate cp-actin-mediated chloroplast positioning, the underlying mechanism remains to be elucidated (Suetsugu et al. 2016; Wada and Kong 2018).

**Figure 4.**
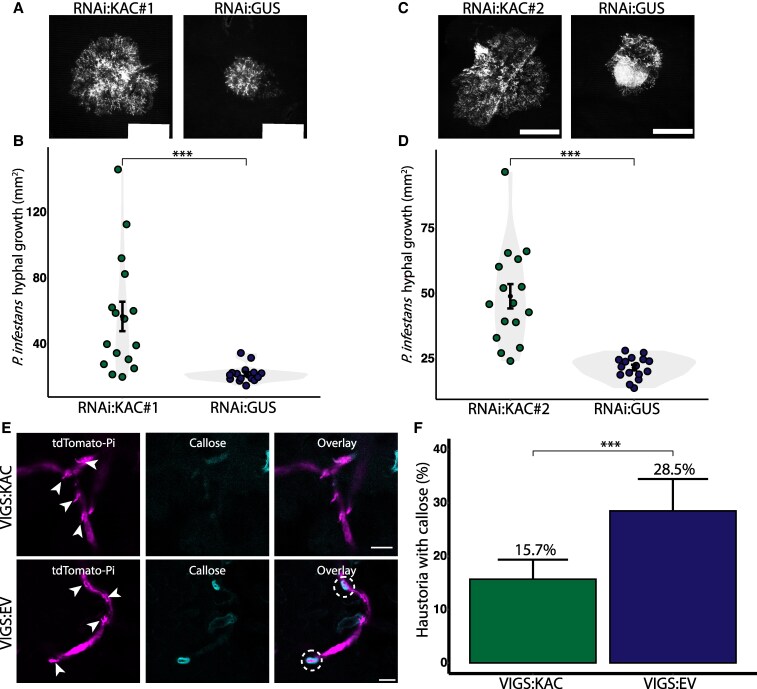
KACs positively contribute to immunity against *Phytophthora infestans* and reduce callose deposition surrounding *P. infestans* haustoria. **A, D)** Silencing *KAC* reduces hyphal growth of *P. infestans*. **A)**  *N. benthamiana* leaves expressing RNAi:KAC#1, or RNAi:GUS control were infected with *P. infestans* expressing tdTomato, and pathogen growth was calculated by measuring hyphal growth using fluorescence stereomicroscope at 5 d post-inoculation. **B)** Violin plot illustrating that RNAi:KAC#1 expression (57.1 mm^2^, *n* = 48 infection spots) significantly increases *P. infestans* hyphal growth compared to RNAi:GUS control (22.1 mm^2^, *n* = 48 infection spots). **C)**  *Nicotiana benthamiana* leaves expressing RNAi:KAC#2, or RNAi:GUS control were infected with *P. infestans* expressing tdTomato, and pathogen growth was calculated by measuring hyphal growth using fluorescence stereomicroscope at 5 d post-inoculation. **D)** Violin plot illustrating that RNAi:KAC#2 expression (49.1 mm^2^, *n* = 48 infection spots) significantly increases *P. infestans* hyphal growth compared to RNAi:GUS control (21.7 mm^2^, *n* = 48 infection spots). Scale bars represent 5 mm. Each dot represents the average of three infection spots on the same leaf. Statistical differences were analyzed by Mann–Whitney U test in R. Error bars represent the mean ± standard error of the mean (SE). Measurements were highly significant when *P* < 0.001 (***). **E, F)** KAC proteins are involved in callose deposition at haustorium penetration sites. **E)** Confocal micrographs of VIGS:KAC and VIGS:EV control *N. benthamiana* leaf epidermal cells. Four-wk-old leaves were infected with *P. infestans* expressing tdTomato, and stained with aniline blue to visualize callose at 3 dpi. Images shown are maximum projection of z-stack images. White arrows indicate haustoria. Scale bars represent 10 *μ*m. **F)** Bar graphs showing VIGS:KAC plants (15.69%, *n* = 376 haustoria) significantly reduce the frequency of callose deposition around haustoria compared to control VIGS:EV plants (28.51%, *N* = 221 haustoria). Statistical differences were analyzed by Fisher's exact test in R. Error bars indicate 95% confidence intervals for the proportion of haustoria with callose deposition. Differences in measurements were considered highly significant when *P* < 0.001 (***).

The *N. benthamiana* genome includes two KAC genes, *KAC1* and *KAC2*. Given that CHUP1 and KACs have overlapping functions, we were intrigued to investigate if silencing KACs could confer a susceptibility phenotype similar to that observed when CHUP1 is knocked out. To explore this, we conducted infection assays upon downregulation of *KAC* expression using two independent hairpin RNAi silencing constructs (Supplementary Fig. S4, A, B, and D). Similar to *CHUP1* silencing (Supplementary Fig. S1, B and C), silencing *KACs* with either RNAi:KAC#1 or RNAi:KAC#2 significantly increased *P. infestans* hyphal growth compared to the control construct RNAi:GUS (Fig. 4, A to D). These results demonstrate that silencing *KACs* lead to plant susceptibility to *P. infestans*.

We next investigated if KACs are also involved in focal immunity like CHUP1 (Fig. 3). To accomplish this, we performed aniline blue staining of infected plant cells upon VIGS of KACs (Supplementary Fig. S4, C and D). We observed a reduction of approximately 44.9% (percentage change of frequency from 28.5% to 12.8%) in the number of haustoria with callose deposits in VIGS:KAC plants compared to VIGS:EV control plants (Fig. 4, E and F). These findings suggest that both CHUP1 and KAC proteins play a role in focal immunity and are involved in proper callose deposition at *P. infestans* haustoria.

### CHUP1 and KAC1 co-operate to tether chloroplasts to the pathogen interface

Having identified that both CHUP1 and KAC proteins play similar positive roles in immunity and are implicated in the focal immune responses against *P. infestans* haustoria, we aimed to explore their potential interplay in membrane tethering of chloroplasts. To address this, we first expressed C-terminally RFP-tagged KAC1 in *N. benthamiana* and investigated its localization pattern using confocal microscopy. Unlike CHUP1, which localizes to the chloroplast outer envelope (Fig. 2, A and C and Supplementary Fig. S2A), KAC1:RFP mainly localizes to the PM and cytosol (Supplementary Fig. S5A). However, KAC1, similar to CHUP1 but not the control EV:RFP, accumulated at MCS between the chloroplasts and the PM (Supplementary Fig. S5, B and C). Remarkably, in *chup1* KO plants, KAC1 did not show any accumulation at the chloroplast–PM contact sites (Supplementary Fig. S5D). This phenotype was restored by the complementation of CHUP1:3xHA co-expression, but not the co-expression of the empty vector control (3xHA:EV) (Supplementary Fig. S5, D and E), demonstrating that KAC1 requires CHUP1 to accumulate at MCS between chloroplasts and the PM. These results indicate that KAC1 might have a direct role in membrane anchoring of chloroplasts in cooperation with CHUP1. This notion is consistent with the previous genetic studies implicating KAC1 and CHUP1 in chloroplast anchoring to the PM in a co-operative manner, as neither mutant was able to show anchorage independently (Suetsugu et al. 2010, 2012). Therefore, we next investigated the potential association between CHUP1 and KAC1. Co-expression of KAC1:RFP and CHUP1:GFP in *N. benthamiana* revealed that both proteins colocalize in punctate structures at MCS between chloroplasts and the PM (Supplementary Fig. S5F). In contrast, the controls, Toc64:GFP and EV:RFP, did not form any punctate structures at these MCS (Supplementary Fig. S5, G and H), showing that not all chloroplast envelope proteins accumulate at the PM contact sites and indicating that CHUP1 and KAC1 cooperate to anchor chloroplasts to membranes.

To expand on these findings, we silenced *KAC1* in *N. benthamiana* using RNAi:KAC#1 and examined CHUP1:GFP localization. Strikingly, in *KAC1*-silenced plants, only 13.3% of chloroplasts near the PM exhibited CHUP1:GFP punctate accumulation, compared to 81.8% in the GUS-silenced control (Supplementary Fig. S6, A and B). These results complement our earlier observation that KAC1 does not accumulate at chloroplast–PM contact sites in chup1 knockout plants (Supplementary Fig. S5, D and E), reinforcing the idea that CHUP1 and KAC1 function together to mediate chloroplast membrane anchoring.

**Figure 5.**
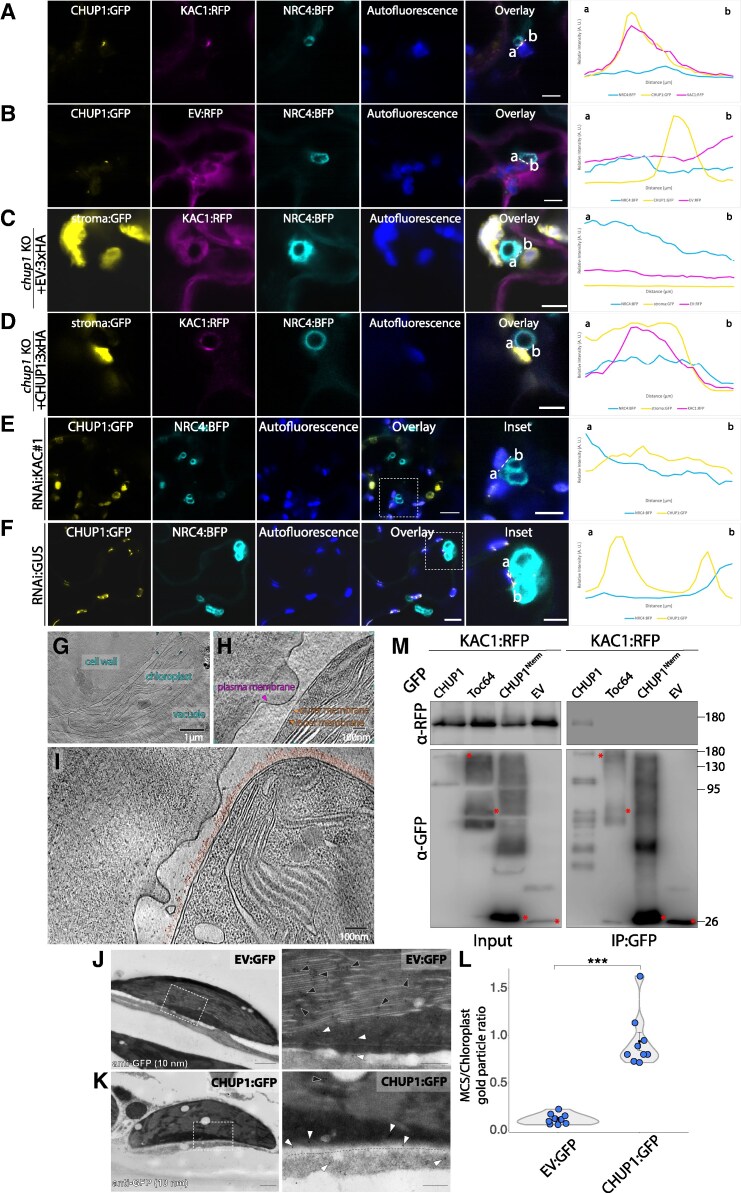
KAC1 interacts with CHUP1 and they colocalize at chloroplast-EHM MCS. **A,B)** CHUP1 colocalizes with KAC1 at punctate structures at chloroplast–EHM MCS. Confocal micrographs of *N. benthamiana* leaf epidermal cells transiently expressing CHUP1:GFP and NRC4:BFP, with **(A)** KAC1:RFP, or **(B)** EV:RFP. Scale bars represent 5 *µ*m. Additional representative images are provided in Supplementary Fig. S7. **C, D)** CHUP1 is required for KAC1 to form punctate structures at chloroplast–EHM MCS. Confocal micrographs of *chup1* KO *N. benthamiana* leaf epidermal cells transiently expressing KAC1:RFP and NRC4:BFP with **(C)** EV:3xHA, or **(D)** CHUP1:3xHA. Chloroplast stroma was visualized with GFP in *chup1* KO plants. Scale bars represent 5 *µ*m. Additional representative images are provided in Supplementary Fig. S7. **E, F)** Silencing KAC1 reduces CHUP1 punctate accumulation at chloroplast-EHM MCS. Confocal micrographs of *N. benthamiana* leaf epidermal cells transiently expressing CHUP1:GFP and NRC4:BFP, with **(E)** RNAi:KAC#1, or **(F)** RNAi:GUS control. Among chloroplasts adjacent to the EHM, KAC1 silencing results in reduced CHUP1 punctate accumulation at chloroplast–EHM MCS (23.0%, *n* = 61 chloroplasts) compared to the GUS-silencing control (72.7%, *n* = 33 chloroplasts). Scale bars represent 10 *µ*m in overlay panels, and 5 *µ*m in inset panels. NRC4:BFP acts as an EHM marker. The leaves were infected with WT *P. infestans* spores at 6 hpi, and imaged at 3 dpi. All presented confocal images are single plane images. Autofluorescence channel depicts chloroplasts. Transects in overlay panels correspond to line intensity plots depicting the relative fluorescence across the marked distance. **G, I)** Membrane contact sites visualized by cryoET. **G)** Slice through a low-magnification tomogram of a chloroplast from a leaf section prepared using serial lift-out Focused Ion Beam milling. Reflections from crystalline ice originating from the cytosol and the vacuole are apparent in the data (see Supplementary Fig. S10). **H, I)** Examples of putative membrane contact sites identified in these data as membrane protrusions. These have not been observed on the PM outside the chloroplast interaction zone. **I)** Slice through a tomogram showing two putative membrane contact sites between the PM and a chloroplast. For visualization, the chloroplast surface was pseudocolored to highlight a ~30 nm thick protein layer, whereas the PM was pseudocolored to highlight fewer visible peripheral membrane proteins. For an overview of the physical slice containing the data shown here and for the correspondence between cryo-fluorescence and cryo-electron microscopy data, see Supplementary Fig. S9. **J, L)** Immuno-transmission electron microscopy (TEM) detection of CHUP1:GFP at chloroplast–PM MCS. *Nicotiana benthamiana* leaves expressing **(J)** EV:GFP or **(K)** CHUP1:GFP were subjected to immunogold labeling using gold particles for GFP antibodies. Right panels show the enlarged TEM images for the insets of dashed-box regions in the left panels. White arrowheads indicate gold particles at chloroplast–PM MCS; black arrowheads indicate gold particles elsewhere on the chloroplast. Dashed lines mark the PM. Scale bars: 600 nm (overview), 200 nm (insets). **L)** Violin plot showing the ratio of gold particles at chloroplast–PM MCS to those elsewhere on the chloroplast. This ratio is significantly higher in CHUP1:GFP samples (0.937, *n* = 9 images) compared to EV:GFP controls (0.119, *n* = 9 images). Statistical differences were analyzed by Mann–Whitney U test in R. Error bars represent the mean ± standard error of the mean (SE). Differences in measurements were considered highly significant when *P* < 0.001 (***). **M)** KAC1 interacts with full length CHUP1, but not with Toc64, CHUP1^Nterm^, or EV:GFP. KAC1:RFP was transiently co-expressed with either CHUP1:GFP, Toc64:GFP, CHUP1^Nterm^:GFP, or EV:GFP. IPs were obtained with anti-GFP antibody. Total protein extracts were immunoblotted. Asterisks indicate band sizes. Numbers on the right indicate kDa values. KO, knockout; EV, empty vector.

Encouraged by these results, we next investigated KAC1–CHUP1 interplay in infected cells. In haustoriated plant cells, we observed that CHUP1 colocalizes with KAC1 at punctate structures at chloroplast–EHM MCS, whereas the empty vector control (EV:RFP) did not exhibit punctate localization (Fig. 5, A and B; Supplementary Fig. S7, A and B). Furthermore, upon infecting *chup1* KO plants expressing KAC1 with *P. infestans*, we found that KAC1 did not form puncta at chloroplast–EHM MCS (Fig. 5C and Supplementary Fig. S7C). Complementing *chup1* KO plants with CHUP1:3xHA reinstated KAC1 punctate formation at chloroplast–EHM MCS (Fig. 5D and Supplementary Fig. S7D). We next assessed the effect of KAC1 silencing on CHUP1 localization in infected cells. Among chloroplasts adjacent to the EHM, KAC1 silencing led to an 83.7% reduction in CHUP1 punctate formation at the chloroplast–EHM interface compared to GUS-silenced control plants (Fig. 5, E and F). These findings collectively suggest that CHUP1 and KAC1 are mutually required to effectively anchor chloroplasts at the plant–pathogen interface, presumably to carry out their immune functions. These results are consistent with our previous findings, which demonstrated that chloroplasts are tightly tethered to the EHM, as revealed by optical tweezer assays (Savage et al. 2021).

Seeking further insight into the molecular architecture of MCS between chloroplasts and the PM, we employed electron cryo-electron tomography (cryoET) in combination with confocal cryo-light microscopy (cryoLM) in plant cells co-expressing CHUP1:GFP and KAC1:RFP. This high-resolution imaging approach revealed well-preserved MCS between the chloroplast outer envelope and the adjacent PM (Fig. 5, G to I; Supplementary Figs. S8 to S10). Notably, we observed distinct membrane protrusions linked by a layer of ∼30 nm long, potentially flexible proteinaceous structure spanning the intermembrane space. These nanoscale in situ structural features suggest the presence of dynamic tethering complexes that bridge the two organelles, reinforcing the proposed roles of CHUP1 and KAC1 in chloroplast anchoring. Moreover, we performed immunogold labeling for transmission electron microscopy (TEM) detection of CHUP1:GFP using samples expressing CHUP1:GFP prepared by high‑pressure‑frozen and freezing substitution methods. Gold particles recognizing GFP were frequently localized to chloroplast–PM MCS in cells expressing CHUP1:GFP, but were randomly distributed in the chloroplast in EV:GFP control cells (Fig. 5, J and K). Quantitative analysis showed a markedly higher MCS‑to‑chloroplast labeling ratio in cells expressing CHUP1:GFP than those in EV:GFP control (Fig. 5L). These findings provide ultrastructural evidence that complements our confocal microscopy and genetic data, showing the existence of the chloroplast–PM MCS, and further implicates CHUP1–KAC1 associations in chloroplast positioning across membrane interfaces.

To corroborate the interplay between CHUP1 and KAC1, we next investigated their *in planta* interaction by performing co-immunoprecipitation (co-IP) assays. We co-expressed KAC1:RFP alongside CHUP1:GFP, or controls Toc64:GFP and CHUP1^Nterm^:GFP, the latter containing the N-terminal region of CHUP1 required for localization to chloroplast outer envelope (Oikawa et al. 2008). Western blot analysis following GFP pulldowns revealed that KAC1:RFP interacts with CHUP1:GFP but not with Toc64:GFP, CHUP1^Nterm^:GFP, or EV:GFP (Fig. 5M), corroborating our confocal microscopy findings that the two proteins associate at chloroplast MCS (Fig. 5A, Supplementary Figs. S5F and S7A).

We next used fluorescence‑lifetime imaging microscopy–Förster resonance energy transfer (FLIM–FRET) to probe molecular proximity *in planta* (Supplementary Fig. S11, A and B). CHUP1:GFP served as the donor and KAC1:RFP as the acceptor, and photon lifetimes were analyzed by average‑arrival‑time FRET (AAT‑FRET) on chloroplasts juxtaposed to the PM (Auer et al. 2023). In CHUP1:GFP‑only controls, the mean weighted arrival time was 2.418 ns, consistent with established GFP lifetimes (Pepperkok et al. 1999). Co-expression of KAC1:RFP shortened the donor lifetime to 2.275 ns. As expected for a maximal FRET pair, a covalent GFP–RFP fusion positive control yielded the shortest lifetime of 2.146 ns. Thus, the observed 143 ps decrease in donor lifetime with KAC1:RFP represents approximately 60% of the maximal FRET efficiency observed with a covalent GFP–RFP fusion in this system (Supplementary Fig. S11, A and B). This 143 ps reduction indicates an estimated donor–acceptor separation of less than 10 nm, consistent with direct or very close molecular association (Forster 1946). Taken together, the complementary co‑IP and FLIM–FRET data provide convergent biochemical and biophysical evidence that CHUP1 and KAC1 form a complex at chloroplast–PM MCS *in planta*, supporting their proposed function as a dynamic tethering module for chloroplast positioning.

CHUP1 is a modular protein that contains an actin-binding domain (ABD) necessary for the formation of chloroplast-actin (cp-actin) filaments, short actin filaments that accumulate at the periphery of chloroplasts and the PM, which are implicated in chloroplast movement and PM anchoring (Oikawa et al. 2003; Kadota et al. 2009). Therefore, we lastly investigated whether the actin-binding function of CHUP1 is necessary for its complex formation with KAC1 and the formation of chloroplast–PM MCS. To address this, we generated GFP fusions of CHUP1ΔABD, a CHUP1 construct lacking the ABD. Confocal microscopy and co-IP assays revealed that the colocalization and interaction of CHUP1 and KAC1 at MCS do not depend on the actin-binding ability of CHUP1 (Supplementary Fig. S12, A and B). However, we noted a slight reduction in the amount of CHUP1 pulled down with KAC1 when the ABD of CHUP1 was omitted (Supplementary Fig. S12B). We conclude that while the ABD of CHUP1 is not essential for CHUP1–KAC1 interaction and their assembly at chloroplast MCS, it could be important for coupling them more tightly to ensure secure docking of chloroplasts at the PM and EHM.

To further examine whether actin contributes to CHUP1–KAC1-mediated MCS formation, we treated *N. benthamiana* leaves with latrunculin A, a toxin that disrupts filamentous actin (F-actin) assembly (Morton et al. 2000). We confirmed actin disruption using Actin:GFP as a marker (Supplementary Fig. S12C). Despite the disruption, CHUP1 and KAC1 continued to colocalize and formed punctate accumulations at chloroplast–PM MCSs in latrunculin A-treated cells, similar to control cells (Supplementary Fig. S12D). These findings indicate that F-actin is not essential for MCS formation. However, we cannot rule out the possibility that specific chloroplast-associated actin (cp-actin) filaments help fine-tune MCS stability or function. Collectively, our results demonstrate that KAC1 interacts with CHUP1 and both proteins colocalize at key membrane contact sites independently of CHUP1's actin-binding ability and intact actin filaments. This interaction likely facilitates chloroplast anchoring at the PM and the plant-pathogen interface, ensuring efficient chloroplast positioning during immune responses.

## Discussion

**Figure 6.**
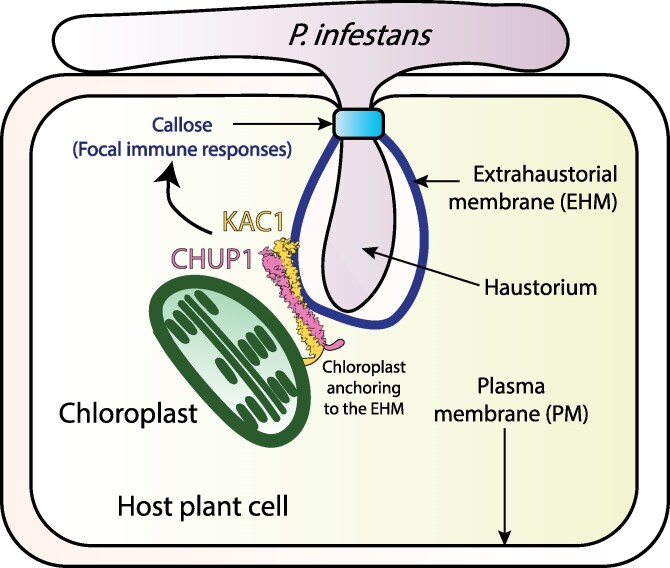
Summary model of CHUP1 and KAC1 coordinating to anchor chloroplasts to the EHM and contribute to plant focal immunity. *P. infestans* penetrates the host plant cell by forming a specialized structure called the haustorium, which is surrounded by a host-derived EHM that acts as the plant–pathogen interface. The chloroplast movement-associated proteins CHUP1 and KAC1 interact to form chloroplast–EHM MCS, thereby anchoring chloroplasts to the EHM. This interaction contributes to plant focal immunity, such as callose deposition and potentially other immune responses at the plant–pathogen interface.

Here, we uncovered MCS between chloroplasts and the EHM in *P. infestans*-infected plant cells. We reveal that these MCS are required for proper immune responses and consist of a membrane anchoring complex comprising the chloroplast outer envelope protein CHUP1 and the PM-associated protein KAC1. Our genetic and cell biology analyses revealed that KAC1 marks these MCS only in the presence of CHUP1. Additionally, our biochemical assays show that CHUP1 and KAC1 interact. We propose a model in which CHUP1 and KAC1 synergistically contribute to plant focal immunity by facilitating MCS at the pathogen penetration sites during infection (Fig. 6).

The role of CHUP1–KAC1 MCS in pathogen resistance is underscored by our findings that depletion of CHUP1 or KAC1 leads to enhanced susceptibility to the adapted pathogen *P. infestans* (Figs. 1 and 4). This susceptibility is accompanied by a reduction in focal immune responses, as evidenced by decreased callose accumulation around the haustorium (Figs. 3 and 4), without affecting other core immune processes. Notably, the *chup1* mutant shows no general defects in chloroplast function, as chloroplast number, chlorophyll content, and core immune signaling (PTI and ETI) remain unaffected (Supplementary Figs. S1 and S3). The shared focal immunity phenotype upon silencing CHUP1 or KAC1, alongside their colocalization at EHM contact sites, suggests a specific role for these MCS in reinforcing immune responses at the pathogen interface. While a direct causal relationship remains to be fully established, our results provide compelling evidence that CHUP1–KAC1-mediated MCS contribute to focal immunity. These results align with findings from other studies showing that *chup1* knockouts in *Arabidopsis thaliana* similarly result in decreased penetration resistance to nonadapted pathogens (Irieda and Takano 2021). Additionally, previous studies have implicated chloroplast outer envelope proteins AtNHRA/B in the proper accumulation of callose (Singh et al. 2018), further supporting our observations that chloroplasts contribute to callose accumulation around the haustorium.

Prior research has also revealed that mitochondria accumulate at pathogen penetration sites and contribute to pathogen penetration resistance, although it remains unknown whether mitochondria can associate with the EHM (Fuchs et al. 2016). Our previous work using optical tweezers showed that chloroplasts can be tightly tethered to the EHM (Savage et al. 2021), but the mechanisms and reasons for this remained unknown. While the precise mechanism by which CHUP1–KAC1 mediates callose deposition around the haustorium remains unknown, their role in tethering chloroplasts to the EHM suggests a significant involvement of chloroplasts in resistance to pathogen penetration. These tethers could ensure secure membrane contacts, facilitating potential short distance material transport through chloroplast–EHM conduits. Indeed, one of the key features of MCS is the direct transfer of lipids, proteins, signaling molecules, ions, and metabolites. It is conceivable that CHUP1–KAC1-labeled MCS facilitate the transport of biochemical precursors necessary for callose synthesis or the transfer of ROS that cross-link and fortify callose deposits (Almagro et al. 2009; Cheval et al. 2020; Castro et al. 2021). These MCS may act as critical hubs for the exchange of materials and signals essential for reinforcing the cell wall at pathogen entry points.

Our current understanding of the mechanisms of chloroplast movement and membrane anchoring originates from studies focusing on chloroplast photorelocation, a process for maximizing photosynthesis and minimizing photodamage based on exposure to light intensities. While dozens of proteins are implicated in chloroplast movement, the detailed molecular interactions and mechanisms remain largely elusive. Previous genetic studies have identified CHUP1 and KAC1 as key players in chloroplast photorelocation (Suetsugu et al. 2012, 2016; Kong et al. 2024). Our findings align with this, showing that CHUP1 accumulates at foci where chloroplasts contact the PM (Fig. 2, A and B). Additionally, we observed that CHUP1 also localizes at EHM contact sites (Fig. 2, E and F), and that KAC1 accumulates at these sites in a CHUP1-dependent manner (Fig. 5, C and D; Supplementary Fig. S5, D and E). Our biochemical assays further support the physical interaction between CHUP1 and KAC1 (Fig. 5M and Supplementary Fig. S12B). These results suggest that the machinery involved in chloroplast photorelocation is co-opted for innate immune responses. Furthermore, blue light receptors, which regulate chloroplast photorelocation, are also implicated in plant immunity (Jeong et al. 2010; Naqvi et al. 2022), underscoring the multifaceted role of these components in both light response and pathogen defense mechanisms.

The exact biochemical and molecular mechanisms through which CHUP1 and KAC1 facilitate MCS tethering chloroplasts to the pathogen interface and contribute to pathogen resistance are yet to be fully elucidated. Future studies are required to determine how these MCS formed by CHUP1 and KAC1 enhance immune responses. Investigating the dynamics of these contact sites and their precise molecular and biochemical functions will be crucial in understanding how they contribute to the targeted deposition of defense components at the pathogen interface. Understanding the intricacies of MCS at the pathogen interface will provide deeper insights into plant defense mechanisms and could inform the development of new strategies to enhance crop disease resistance.

## Materials and methods

### Plant growth details

*Nicotiana benthamiana* plants (WT and transgenics) were cultivated under controlled conditions in a growth chamber maintained at a temperature of 24 °C. They were grown in a substrate mixture comprising organic soil (3:1 ratio of Levington's F2 with sand and Sinclair's 2 to 5 mm vermiculite). The plants were exposed to high-intensity light and subjected to a long-day photoperiod (16 h of light followed by 8 h of darkness). Experiments were conducted using plants aged 4 to 5 wk. Among the transgenic plants utilized were CP plants (Savage et al. 2021), expressing GFP in the chloroplast stroma, and FNR plants (Erickson et al. 2017), expressing FNR:eGFP in the chloroplast. FNR plants served as the parental lines for *chup1* KO plants and were employed as controls in *chup1* KO experiments.

### Pathogen growth details and infection assay

WT and tdTomato-expressing *P. infestans* 88069 isolates were cultured on rye sucrose agar (RSA) medium in darkness at 18 °C for 10 to 15 d before harvesting zoospores. Zoospores were collected by adding cold water at 4 °C to the medium and then incubating it in darkness at 4 °C for 90 min. For the infection assay, 10 *µ*l droplets of zoospore solution containing 50,000 spores/ml were applied to the underside of leaves. The leaves were subsequently maintained in a humid environment. Confocal microscopy was conducted 3 d post-infection (dpi), and fluorescent images were captured at 5 dpi. Hyphal growth was quantified and analyzed using ImageJ.

### Generation of *N. benthamiana* CHUP1 CRISPR knockout plants

To generate CHLOROPLAST UNUSUAL POSITIONING 1 (*chup1*) KO and *chup1* KO#2 plants, the following primer pairs were used to create guide RNA sequences targeting both *NbChup1* alleles: attGCAAGATCAAGGAGTTGCAG & aaacCTGCAACTCCTTGATCTTG; attgTGGACTTCAAGAAAAGGAAG & aaacCTTCCTTTTCTTGAAGTCCA; and attgTCTGTATCATACTTGTCACT & aaacAGTGACAAGTATGATACAGA. The genome editing vector used was pDGE463 (Stuttmann et al. 2021). This vector contains: plant kanamycin resistance for positive selection of transgenic plants, the Bs3 gene from pepper for negative selection, 2xtagRFP expressed by a seed coat-specific promoter for negative selection, a p35S-driven intronized Cas9 with 2xNLS for targeted gene editing, and bacterial spectinomycin resistance for selection in bacteria. The guide RNAs were inserted into pDGE463 to create the new vector, pDGE472. The primers used for genotyping *chup1* KO plants were CHUP1KO_genotype_F and CHUP1KO_genotype_R, while the primers used for genotyping *chup1* KO#2 plants were CHUP1KO2_genotype_F and CHUP1KO2_genotype_R. The parental line transformed was NbFNR:eGFP_7–25 (FNR) for *chup1* KO plants (Schattat et al. 2011), and the parental line transformed was WT for *chup1* KO#2 plants.

### Molecular cloning

The molecular cloning of KINESIN-LIKE PROTEIN FOR ACTIN-BASED CHLOROPLAST MOVEMENT 1 (KAC1), CHUP1, CHUP1^Nterm^, CHUP1ΔABD, MEK2^DD^, GFP:RFP:Rab8a, and NLS was conducted using Gibson Assembly, following the methods described in previous works (Dagdas et al. 2016; Yuen et al. 2024b). Specifically, the vector backbone was a pK7WGF2 derivative domesticated for Gibson Assembly, containing a C-terminal fluorescent GFP, RFP, BFP, or 3xHA tag. The desired sequences for cloning were either manufactured as a synthetic fragment or amplified using designed primers as detailed in Supplementary Tables S1 and S2. CHUP1ΔABD was created using two fragments that were amplified with two sets of primers, excluding the ABD flanked by the fragments. The fragments were then inserted into the vector using Gibson Assembly and transformed into DH5α chemically competent *Escherichia coli* through heat shock. These plasmids were subsequently amplified and extracted using the PureYield Plasmid Miniprep System (Promega), and electroporated into *Agrobacterium tumefaciens* GV3101 electrocompetent cells. Sequencing was done by Eurofins. LB agar containing gentamicin and spectinomycin was used to grow bacteria carrying the pK7WGF2 plasmid. The NLS:BFP construct was created by joining a cut pK7WGF2-derived C-terminal BFP vector with a single-stranded DNA oligo bridge using Gibson assembly. The single-stranded DNA oligo bridge had overhangs complementary to the cut vector ends and included the coding sequence for the SV40 nuclear localization sequence (PKKKRKVEDP). For VIGS, TRV2:CHUP1 was constructed by amplifying a region of the CHUP1 sequence using the primer pair CHUP1_sil_F and CHUP1_sil_R. The amplified fragment was then cloned into a Gateway-compatible pTRV2 vector using Gateway cloning technology (Invitrogen). TRV2:KAC for VIGS was constructed by amplifying a region of the KAC1 sequence using the primer pair KAC1_sil_F and KAC1_sil_R. The amplified fragment was then cloned into a Golden Gate-compatible TRV2-GG vector using Golden Gate cloning (Duggan et al. 2021). LB agar containing gentamicin and kanamycin was used to grow bacteria carrying pTRV2 and TRV2-GG plasmids. For the RNA interference (RNAi) silencing construct RNAi:KAC, an intron-containing hairpin RNA vector for RNA interference in plants (pRNAi-GG) was employed, based on the Golden Gate cloning method described in previous studies (Yan et al. 2012; Yuen et al. 2024b). RNAi:KAC#1 targeted the region between 3,512 and 3,709 bp of *NbKAC1/2*. The target fragment was amplified using KAC1_sil_F and KAC1_sil_R. RNAi:KAC#2 targeted the region between 1,003 and 1,184 bp of *NbKAC1* and 2,191 and 2,427 bp of *NbKAC2*. The target fragment was synthesized. Each target fragment was then inserted into the pRNAi-GG vector in both sense and antisense orientations, utilizing the overhangs generated by BsaI cleavage. This resulted in the expression of a construct that folds back onto itself, forming the silencing hairpin structure. The subsequent steps of *E. coli* transformation, Miniprep, sequencing, and Agrobacterium transformation were the same as those used for the overexpression constructs. LB agar containing gentamicin, kanamycin, and chloramphenicol was used to grow bacteria carrying the pRNAi-GG plasmid. All primers and synthetic fragments used in this study are detailed in Supplementary Tables S1 and S2. All constructs used in this study are detailed in Supplementary Table S3.

### Confocal laser scanning microscopy

The confocal microscopy analyses were conducted 3 d following agroinfiltration. To visualize the leaf tissue, the leaves were excised using a size 4 cork borer, mounted live on glass slides, and submerged in wells of dH_2_O using Carolina observation gel (Carolina Biological). Imaging of the abaxial side of the leaf tissue was performed using either a Leica TCS SP8 inverted confocal microscope equipped with a 40× water immersion objective lens or a Leica STELLARIS 5 inverted confocal microscope equipped with a 63× water immersion objective lens. Laser excitations for GFP, RFP/tdTomato, and BFP tags were set at Argon 488 nm (15%), DPSS 561 nm, and Diode 405 nm, respectively. Collection bandwidths for GFP, RFP/tdTomato, and BFP tags were 495 to 550 nm, 570 to 620 nm, and 402 to 457 nm, respectively. The detector gain is 50.0, and the pinhole size is 1.00 airy unit. To prevent spectral overlap from different fluorescent tags when imaging samples with multiple tags, sequential scanning between lines was employed. Confocal images were analyzed using ImageJ.

### Confocal image analysis

The number of chloroplasts was automatically counted using ImageJ. Maximum intensity z-projections of z-stack images were created, and the channels were split. The autofluorescence channel was then automatically thresholded using the “Li” method, followed by the application of a watershed. Objects were counted using the “Analyze Particles” command with a size filter of 5 to 35.

For quantifying chloroplast–haustoria association, haustoria were first identified in a given z-stack using only the RFP and brightfield channels to ensure that their identification was not influenced by the position of chloroplasts. The number of haustoria associated with a chloroplast, including those connected via a stromule, was then counted. The percentage of haustoria associated with chloroplasts was calculated based on the total number of identified haustoria. To avoid skewing the data with images containing a single haustorium, which could result in extreme percentages (0% or 100%), the overall percentage for the entire data set was used instead of calculating percentages on an image-by-image basis.

Quantifying perinuclear chloroplast clustering was challenging due to the close packing of chloroplasts around the nucleus, making it difficult to resolve individual chloroplasts. To measure perinuclear clustering, the total volume of thresholded chlorophyll autofluorescence around the nuclei was quantified. First, nuclei were automatically identified from maximum intensity z-projections of z-stack images, with channels split. The NLS:BFP channel was automatically thresholded using the “Li” method, followed by the application of a watershed. Nuclei were then counted using the “Analyze Particles” command with a size filter of 100 to 500. The positions of the identified nuclei were saved as regions of interest (ROIs). For each ROI, the original image was duplicated and cropped to a 70 × 70 pixel region around the nucleus, which was then saved separately for further analysis. The quality of these cropped images was manually assessed. If necessary, the following adjustments were made: (i) Slices of the z-stack containing the mesophyll layer were removed. (ii) Crops were resized to exclude chloroplasts from adjacent cells or those not in immediate contact with the nucleus. The chloroplast autofluorescence channel was then isolated and automatically thresholded using the “Li” method. For each slice of the z-stack, pixels with a value of 255 were counted, and the voxel size was determined for each image. This data was compiled into a custom table containing pixel count, voxel size, and image name for all identified nuclei. Using this table, the cubic microns of thresholded chloroplast autofluorescence were calculated for each identified nucleus, providing a metric for perinuclear chloroplast clustering.

### Nonfitting FLIM–FRET

Nonfitting FLIM–FRET was used to detect interactions between CHUP1 and KAC1 by measuring the mean weighted photon arrival time per pixel in regions where chloroplasts contacted the PM, as described before (Auer et al. 2023). Samples were imaged on a Leica Stellaris 5 point-scanning confocal microscope equipped with a HC PL APO CS2 63×/1.20 WATER objective. The scanner speed was set to 200 Hz in unidirectional mode, with images acquired at 128 × 128 pixels and a zoom factor of 6, resulting in an image size of 30.75 *μ*m and a pixel size of 242 nm. Line accumulation was set to 6. For CHUP1:GFP, excitation was performed using a White Light Laser at 485 nm, and emission along with photon arrival times were recorded in TauContrast and TauInteraction modes using a HyD S detector (490 to 520 nm). For KAC1:RFP, excitation was performed at 561 nm, and emission was detected in analog mode using a HyD S detector (569 to 630 nm). Data were analyzed using LAS X software (Leica Microsystems, version 1.4.5.27713).

### Fluorescence microscopy for *N. benthamiana*

Fluorescence microscopy was employed to observe the hyphal growth of *P. infestans* expressing tdTomato. The imaging setup included a Leica MZ 16 F microscope paired with the Leica DFC300 FX Digital Color Camera, specifically designed for fluorescence imaging. Infected leaf samples were placed on a petri dish within the imaging zone of the microscope. The imaging filter utilized was DsRed, with an excitation range ranging from 510 to 560 nm.

### High-pressure freezing for cryoET

*Nicotiana benthamiana* leaves, at 4 dpi with CHUP1:GFP and KAC1:RFP, were infiltrated with 1-hexadecane immediately before freezing in HPM010 (Baltec) using the 200 *µ*m recess of a type-A carrier paired with the flat side of a type-B carrier (Wohlwend).

### Serial lift-out and two step cryogenic correlative light and electron microscopy

Finding regions of interest: Carriers containing high-pressure frozen leaves were loaded into either a Stellaris 5 or Stellaris 8 (Leica) microscope equipped with a cryogenically cooled stage. Screening was performed at relatively low magnification (∼500 nm/pixel) to find chloroplasts with bright GFP and RFP signals. Confocal volumes were acquired with ∼150 nm/pixel and 1.5 *µ*m Z step. The carriers were then transferred to Aquilos 2 (Thermo) dual beam microscope equipped with an integrated fluorescence microscope (iFLM).

Serial lift-out was performed in a similar manner as described previously (Schiøtz et al. 2024). The details of this procedure are highlighted in Supplementary Fig. S8. Following platinum sputtering and coating with an organoplatinum layer using a gas injection system, a block ∼50 × 25 × 15 *µ*m was milled with a 16 nA probe. Attachment (welding) was performed using a single pass regular cross-section pattern (0.3 to 1 nA). Attachment to a lift-out needle was done via a copper block (larger surface area for a more secure attachment), the block was lifted from the carrier and transferred to a copper 400 × 100 mesh grid (Agar Scientific). It was positioned over two grid bars, welded in place and a line cut was used to cut 2 to 3 *µ*m slices. Slices were trimmed to remove material in front of and behind regions of interest (located using iFLM) and milled to ∼500 nm thickness.

On-lamella correlation: The ∼500 nm thick lamella were transferred into Stellaris 8 to acquire data with ∼100 nm/pixel magnification using photon counting and tau gating to remove autofluorescent signal. The imaging and transfer steps here result in the accumulation of ice crystals that obscure features in TEM. The specimen was therefore transferred back to Aquilos 2 and the lamella were polished to ∼50 to 200 nm thickness. Final iFLM images were acquired on the polished lamella, to make sure that features of interest were not removed in the final thinning (via comparison the confocal data).

### Cryo-ET data acquisition and data processing

Transmission electron microscope data were collected using a Titan Krios (Thermo) equipped with K3 and BioQuantum energy filter (Gatan). Tilt series were acquired at 2.648 Å pixel-1 at 3° increments from −66° to 66° with fluence of 3.9e^−^/Å^2^ per tilt using SerialEM and PACE tomo (Mastronarde 1997; Hagen et al. 2017; Eisenstein et al. 2023). A 70 *µ*m objective aperture and a 20 nm energy-selecting slit was used during acquisition. Tomogram reconstruction was done in IMOD (Kremer et al. 1996). Membrane segmentation was done using MemBrain (Lamm et al. 2024). Data visualization was done in ChimeraX (Pettersen et al. 2021), IMOD, and Fiji (Schindelin et al. 2012).

### Immunogold labeling and TEM

*Nicotiana benthamiana* leaves were fixed with 2% paraformaldehyde and 2.5% glutaraldehyde in 0.1 m sodium cacodylate buffer (pH 7.4; Electron Microscopy Sciences, 15960). After washing with 200 mm phosphate buffer, the samples were frozen using a high-pressure freezer (Leica Microsystems, ICE) and freeze-substituted in an automatic freeze-substitution machine (Leica Microsystems, AFS2). Acetone containing 0.25% glutaraldehyde (Electron Microscopy Sciences, 16200) and 0.1% uranyl acetate (Electron Microscopy Sciences, 22400) was used as the substitution medium, and Lowicryl HM20 resin (Electron Microscopy Sciences, 14340) was used for embedding. Ultrathin sections (100 nm) were prepared using an ultramicrotome (Leica UC7). Immunogold labeling was performed as described before (Chung et al. 2024) using anti-GFP antibodies (Rockland, 600-401-215) and 10 nm gold particle-coupled secondary antibodies (Electron Microscopy Sciences, 25108). TEM imaging was performed using a Hitachi H-7650 TEM (Hitachi-High Technologies) with a CCD (Charged Coupled Device) camera operating at 80 kV operated at 80 kV.

### Agrobacterium-mediated transient gene expression in *N. benthamiana*

Agrobacterium-mediated transient gene expression was carried out via agroinfiltration, following a well-established protocol (Bozkurt et al. 2011). *Agrobacterium tumefaciens* containing the desired plasmid was rinsed with water and then suspended in agroinfiltration buffer (10 mm MES, 10 mm MgCl_2_, pH 5.7). The optical density (OD_600_) of the bacterial suspension was measured using the BioPhotometer spectrophotometer (Eppendorf). Subsequently, the suspension was adjusted to the required OD_600_ according to the specific construct and experimental conditions. The adjusted bacterial suspension was then infiltrated into *N. benthamiana* leaf tissue aged 3 to 4 wk using a needleless 1 ml Plastipak syringe.

### RNA isolation, cDNA synthesis, and RT-PCR

To perform RNA extraction, 56 to 70 mg of leaf tissue was promptly frozen in liquid nitrogen. The RNA extraction process employed the Absolutely Total RNA Purification Kits (Agilent Technologies). Subsequently, RNA concentration was quantified using NanoDrop Lite Spectrophotometer (Thermo Scientific). Two milligram of the extracted RNA underwent treatment with RQ1 RNase-Free DNAse (Promega) before being used for cDNA synthesis with SuperScript IV Reverse Transcriptase (Invitrogen). The resulting cDNA was then amplified using Phusion High-Fidelity DNA Polymerase (New England Biolabs) or DreamTaq DNA polymerase (Thermo Scientific). *GAPDH* transcription level was utilized as the transcriptional control. The RT-PCR for *GAPDH* was performed using the primers GAPDH_RTPCR_F and GAPDH_RTPCR_R. The RT-PCR confirming VIGS:CHUP1 was performed using the primers NbCHUP1a_RTPCR_F and NbCHUP1a_RTPCR_R for *NbCHUP1a* and NbCHUP1b_RTPCR_F and NbCHUP1b_RTPCR_R for *NbCHUP1b*. The RT-PCR confirming RNAi:KAC and VIGS:KAC was performed using the primers KAC1_RTPCR_F and KAC1_RTPCR_R for *NbKAC1* and KAC2_RTPCR_F and KAC2_RTPCR_R for *NbKAC2*. All primers used in this study are detailed in Supplementary Table S1.

### Chlorophyll extraction

Three leaf discs, obtained with a size 4 cork borer, were collected from three distinct leaves of each plant. Each leaf disc was then ground in 10 ml of methanol for 1 min and centrifuged at 385 × *g* for 5 min. Absorbance readings at 666 and 653 nm were recorded, and the total chlorophyll concentration was determined following a well-established method detailed by Wellburn (1994).

### Co-immunoprecipitation and immunoblot analyses

Proteins were transiently expressed in *N. benthamiana* leaves through agroinfiltration, and the harvest took place 3 d after agroinfiltration. Co-immunoprecipitation experiments utilized 2 g of leaf tissues. The procedures for protein extraction, purification, and immunoblot analysis followed the previously described protocols (Bozkurt et al. 2011). The primary antibodies used included monoclonal anti-RFP produced in mouse (Chromotek), monoclonal anti-GFP produced in rat (Chromotek), polyclonal anti-phospho-MAPK produced in rabbit (Cell Signaling Technology), and monoclonal HRP-conjugated antibeta actin produced in mouse (Proteintech). As for secondary antibodies, antirabbit antibody (Sigma–Aldrich), antirat antibody (Sigma–Aldrich), and antimouse antibody (Sigma–Aldrich) for HRP detection were employed. Comprehensive information regarding the antibodies used is detailed in Supplementary Table S4.

### Cell death assay

Cell death inducers were introduced into the underside of *N. benthamiana* leaves via agroinfiltration. Afterward, at 3 dpi, the leaves were detached and examined under both natural light conditions. The degree of cell death was assessed using an established seven-tiered cell death scale (Wu et al. 2017).

### *Phytophthora infestans* extract (Pi extract) preparation and injection

Mycelia harvested from *P. infestans* RSA plates were gathered and suspended in 5 ml of water per petri dish. The suspension underwent vortexing for 1 min and was then subjected to heating at 95 °C for 20 min. Following this, the mixture was filtered through filter paper with a pore size ranging from 5 to 13 *µ*m. The resulting filtrate underwent an additional filtration step using a syringe filter with a pore size of 0.45 *µ*m. This resulting solution was then administered to plants to function as a PAMP cocktail, and is stored at −20 °C.

### Latrunculin A treatment

Latrunculin A treatment was performed as described before (Savage et al. 2021). Latrunculin A (abcam, ab144290) was dissolved in 100% DMSO to a stock concentration of 100 *μ*m. The water control contained an equivalent final concentration of DMSO (v/v). Both latrunculin A (1.5 *μ*l working concentration) and the water control were infiltrated into leaf tissue using needleless syringes 24 h before confocal microscopy.

### Virus-induced gene silencing

Agrobacterium was prepared as described above, carrying TRV1 and the respective TRV2 construct, and mixed to achieve final OD_600_ values of 0.4 or 0.2, respectively, in agroinfiltration buffer supplemented with 100 *µ*m acetosyringone (Sigma–Aldrich). The mixture was then kept in the dark for 2 h prior to infiltration to enhance virulence. Fourteen-day-old *N. benthamiana* seedlings were infiltrated in both cotyledons and any emerged true leaves. For CHUP1-silencing, *N. benthamiana* plants were infiltrated with TRV1 and TRV2:CHUP1, while for KAC1-silencing, TRV1 and TRV2:KAC were used. The empty vector control was achieved by infiltrating TRV1 and TRV2:EV. Plants were allowed to grow under standard conditions until experiments could be conducted 3 wk later.

### Image processing and data analysis

The confocal microscopy images were processed using Leica LAS X software and ImageJ. ImageJ was employed for analyzing and quantifying the infection assay experiments. Data were presented using violin plots, box plots, and bar graphs created with R. Statistical differences were evaluated using appropriate tests that consider normality and variance. Significance levels were denoted as follows: * (*P* < 0.05), ** (*P* < 0.01), and *** (*P* < 0.001). Detailed information regarding the statistical tests utilized can be found in the figure captions, and extensive statistical calculations are available in Supplementary Table S5.

### Accession numbers

CHUP1a (Sol Genomics Network: Niben101Scf00570g03008/9.1; NbenBase: Nbe.v1.1.chr08g25910); CHUP1b (Sol Genomics Network: Niben101Scf01338g03019.1); KAC1 (NbenBase: Nbe.v1.1.chr13g42800); KAC2 (NbenBase: Nbe.v1.1.chr05g36640); Toc64 (GenBank: At3g17970); REM1.3 (GenBank: P93788.1); NRC4 (GenBank: MK692737).

## Supplementary Material

koaf214_Supplementary_Data

## Data Availability

The data underlying this article are available and included in the article, and in the Supplementary Materials files.

## Supplementary Data

The following materials are available in the online version of this article.

**Supplementary Figure S1.** CHUP1 promotes plant immunity and the increased disease susceptibility observed in *chup1* KO plants is not attributed to impaired chloroplast positioning or abnormal chloroplast numbers.

**Supplementary Figure S2.** CHUP1 forms punctate accumulation at chloroplast–EHM MCS, but the control Toc64 does not.

**Supplementary Figure S3.** The activation of PAMP- or effector-triggered immunity is not impaired in the absence of CHUP1.

**Supplementary Figure S4.** Validation of KACs silencing using the RNAi:KAC#1, RNAi:KAC#2 constructs and VIGS:KAC plants.

**Supplementary Figure S5.** KAC1 localizes to PM and requires CHUP1 to accumulate at MCS between chloroplasts and the PM.

**Supplementary Figure S6.** Silencing KAC1 reduces CHUP1 punctate accumulation at chloroplast–PM MCS.

**Supplementary Figure S7.** KAC1 colocalizes with CHUP1 at chloroplast–EHM MCS.

**Supplementary Figure S8.** Serial lift-out of high-pressure frozen leaves and two-step on-lamella correlative light and electron microscopy.

**Supplementary Figure S9.** Correlative serial lift-out cryoET visualizes molecular detail inside intact vitreous chloroplasts.

**Supplementary Figure S10.** Incomplete vitrification observed in the high-pressure frozen specimen.

**Supplementary Figure S11.** Nonfitting FLIM–FRET analysis shows CHUP1 and KAC1 interact *in planta*.

**Supplementary Figure S12.** CHUP1 colocalizes and interacts with KAC1 independent of the ABD of CHUP1 and under latrunculin A treatment.

**Supplementary Table S1.** List of primers used in this study.

**Supplementary Table S2.** List of synthetic fragments used in this study.

**Supplementary Table S3.** List of constructs used in this study.

**Supplementary Table S4.** List of antibodies used in this study.

**Supplementary Table S5.** Summary of Statistics.

## Dive Curated Terms

The following phenotypic, genotypic, and functional terms are of significance to the work described in this paper:

CHUP1 Gramene: AT3G25690
CHUP1 Araport: AT3G25690
