# Supplementary material for: Membrane contact sites between chloroplasts and the pathogen interface underpin plant focal immune responses
Source: Plant Cell. 2025 Sep 5;37(9):koaf214. doi: 10.1093/plcell/koaf214 (PMC12481158; doi:10.1093/plcell/koaf214)
Supplement: koaf214_Supplementary_Data [file koaf214_supplementary_data.zip › TPC-2025-0380 Supplemental Figures and Tables.pdf]

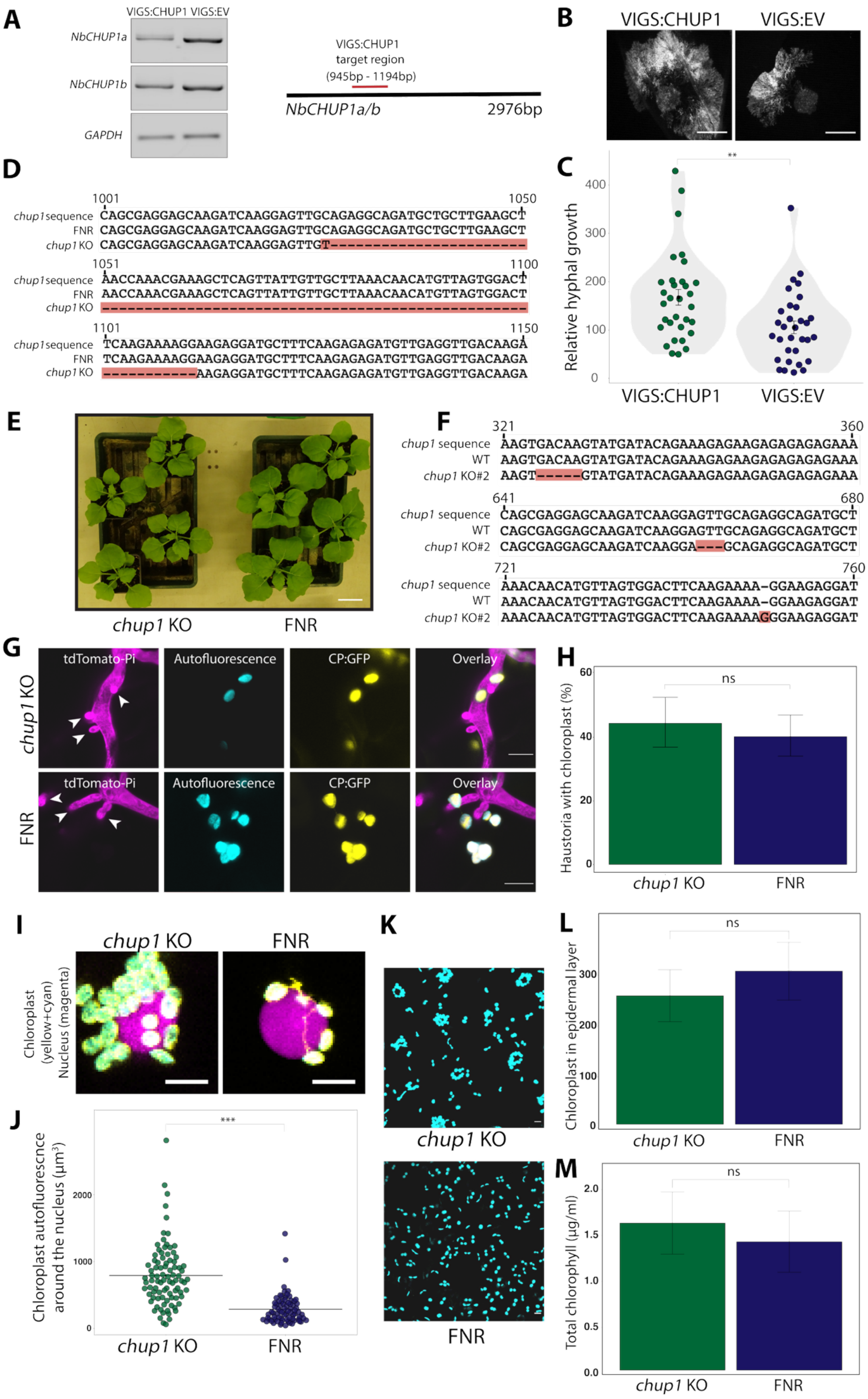
A
VIGS:CHUP1 VIGS:EV
NbCHUP1a
NbCHUP1b
GAPDH
VIGS:CHUP1
target region
(945bp - 1194bp)
NbCHUP1a/b
2976bp
B
VIGS:CHUP1
VIGS:EV
C
**
Relative hyphal growth
400
300
200
100
0
VIGS:CHUP1
VIGS:EV
D
1001
1050
chup1 sequence CAGCGAGGAGCAAGATCAAGGAGTTGCAGAGGCAGATGCTGCTTGAAGCT
FNR CAGCGAGGAGCAAGATCAAGGAGTTGCAGAGGCAGATGCTGCTTGAAGCT
chup1 KO CAGCGAGGAGCAAGATCAAGGAGTTGT-----------------------
1051
1100
chup1 sequence AACCAAACGAAAGCTCAGTTATTGTTGCTTAAACAACATGTTAGTGGACT
FNR AACCAAACGAAAGCTCAGTTATTGTTGCTTAAACAACATGTTAGTGGACT
chup1 KO --
1101
1150
chup1 sequence TCAAGAAAAGGAAGAGGATGCTTTCAAGAGAGATGTTGAGGTTGACAAGA
FNR TCAAGAAAAGGAAGAGGATGCTTTCAAGAGAGATGTTGAGGTTGACAAGA
chup1 KO -----------AAGAGGATGCTTTCAAGAGAGATGTTGAGGTTGACAAGA
E
chup1 KO
FNR
F
321
360
chup1 sequence AAGTGACAAGTATGATACAGAAAGAGAAGAGAGAGAGAAA
WT AAGTGACAAGTATGATACAGAAAGAGAAGAGAGAGAGAAA
chup1 KO#2 AAGT------GTATGATACAGAAAGAGAAGAGAGAGAGAAA
641
680
chup1 sequence CAGCGAGGAGCAAGATCAAGGAGTTGCAGAGGCAGATGCT
WT CAGCGAGGAGCAAGATCAAGGAGTTGCAGAGGCAGATGCT
chup1 KO#2 CAGCGAGGAGCAAGATCAAGGA---GCAGAGGCAGATGCT
721
760
chup1 sequence AAACAACATGTTAGTGGACTTCAAGAAAA-GGAAGAGGAT
WT AAACAACATGTTAGTGGACTTCAAGAAAA-GGAAGAGGAT
chup1 KO#2 AAACAACATGTTAGTGGACTTCAAGAAAAGGGAAGAGGAT
G
chup1 KO
FNR
tdTomato-Pi
Autofluorescence
CP:GFP
Overlay
H
ns
Haustoria with chloroplast (%)
60
40
20
0
chup1 KO
FNR
I
chup1 KO
FNR
Chloroplast (yellow+cyan)
Nucleus (magenta)
J
***
Chloroplast autofluorescnce around the nucleus (µm³)
2000
1000
0
chup1 KO
FNR
K
chup1 KO
FNR
L
ns
Chloroplast in epidermal layer
300
200
100
0
chup1 KO
FNR
M
ns
Total chlorophyll (µg/ml)
2.0
1.5
1.0
0.5
0.0
chup1 KO
FNR

Supplementary Figure S1. CHUP1 positively contributes to plant immunity and the increased disease susceptibility observed in *chup1* KO plants is not attributed to impaired chloroplast positioning or abnormal chloroplast numbers. (A) Validation of *CHUP1* silencing of VIGS:CHUP1 plants. Constructs carrying TRV1 with TRV2-GG targeting *NbCHUP1a and NbCHUP1b* or the control TRV2:EV were infiltrated to *N. benthamiana* leaves. The expression levels of the targeted genes were assessed via RT-PCR at 3 weeks post VIGS. The RT-PCR employs primers NbCHUP1a_RTPCR_F and NbCHUP1a_RTPCR_R for *NbCHUP1a*, and NbCHUP1b_RTPCR_F and NbCHUP1b_RTPCR_R for *NbCHUP1b*. RT-PCR results confirmed gene silencing of *NbCHUP1a* and *NbCHUP1b* in the VIGS:CHUP1 plants. Glyceraldehyde 3-phosphate dehydrogenase (GAPDH) served as the internal control, using primers GAPDH_RTPCR_F and GAPDH_RTPCR_R for assessment. The cDNA was synthesized using total RNA. (B) WT *N. benthamiana* leaves were infected with tdTomato-expressing *P. infestans* 3 weeks post VIGS, and pathogen growth was calculated by measuring hyphal growth using fluorescence stereomicroscope at 5 days post-inoculation. Scale bars represent 5 mm. (C) Violin plot illustrating that plants treated with VIGS:CHUP1 construct (167.7, n = 192 infection spots) show a significant increase in *P. infestans* hyphal growth compared to plants treated with control VIGS:EV construct (105.6, n = 192 infection spots). Each dot represents the average of 6 infection spots on the same leaf. Statistical differences were analyzed by Mann-Whitney U test in R. Error bars represent the mean ± standard error of the mean (SE). Measurements were significant when $p<0.01$. (**). (D) Generation of *N. benthamiana chup1* CRISPR knockout mutant, designated as *chup1* KO plants, in an FNR:eGFP background. *chup1* KO plants contain a 84 nt deletion that introduces premature stop codons in the *chup1* gene. (E) Daylight photographs of representative four-week-old *chup1* KO and FNR plants used for experiments. Scale bar represents 5 cm. (F) Generation of *N. benthamiana chup1* CRISPR knockout mutant, designated as *chup1* KO#2 plants, in a WT background. *chup1* KO#2 plants contain three editing sites in the *chup1* gene. First site contains a 5 nt deletion and causes a frameshift with stop codons downstream. Second site contains a 3 nt deletion. Third site contains a 1 nt insertion. These mutations do not reconstitute the reading frame. (G-H) Chloroplast positioning at haustoria is unaffected by the loss of CHUP1. (G) Confocal micrographs depict *chup1* KO and FNR control *N. benthamiana* leaf epidermal cells. Four-week-old leaves were infected with tdTomato-expressing *P. infestans*. Imaging was performed 3 days post-inoculation. White arrows indicate haustoria. Yellow and cyan show chloroplast stroma and chloroplast autofluorescence respectively. Scale bars represent 10 µm. (H) Bar graphs demonstrate that the differences in chloroplast-haustoria associations between *chup1* KO plants (44.38%, n = 160 haustoria) and FNR plants (40.17%, n = 229 haustoria) are not significant. Error bars represent standard deviation. Statistical significance was assessed using Fisher's exact test in R. (I-J) Chloroplasts in *chup1* KO plants are clustered around the nucleus. (I) Confocal micrographs depicting nuclei in *chup1* KO and FNR plants transiently expressing NLS:BFP (magenta). Yellow and cyan show chloroplast stroma and chloroplast autofluorescence respectively. Scale bars represent 10 µm. (J) Quantification of chloroplast autofluorescence volume surrounding nuclei, marked by transiently expressed NLS:BFP, in *chup1* KO and FNR plants. *chup1* KO plants (791, n = 89 nuclei) exhibit a significant increase in chloroplast clustering around the nucleus compared to control FNR plants (286, n = 74 nuclei). Crossbars represent the mean. Statistical significance was determined using the Mann-Whitney U test in R. Each data point represents a measurement from a single isolated nucleus. (K) Confocal micrographs depict *chup1* KO and FNR plants where the entire depth of the epidermal layer is shown. Chloroplasts are depicted in cyan colour. Images shown are maximum projection of z-stack images. Scale bars

represent 10 µm. 3D projections can be found as Supplementary Movies S1 and S2. (L) Bar graphs demonstrate that the differences in the number of chloroplasts in the epidermal layer between *chup1* KO plants (253, n = 9 micrographs) and FNR plants (301, n = 9 micrographs) are not significant. Error bars represent standard deviation. Statistical significance was assessed using Student's t-test in R. (M) Bar graphs demonstrate that the differences in the total chlorophyll concentration between *chup1* KO plants (1.6 µg/ml, n = 3 plants) and FNR plants (1.4 µg/ml, n = 3 plants) are not significant. Error bars represent standard deviation. Statistical significance was assessed using Mann-Whitney U test in R. Measurements were highly significant when $p < 0.001$ (***), significant when $p < 0.01$ (**), and not significant (ns) when $p \geq 0.05$. VIGS: virus-induced gene silencing; EV: empty vector; KO: knockout. Supports Figure 1.

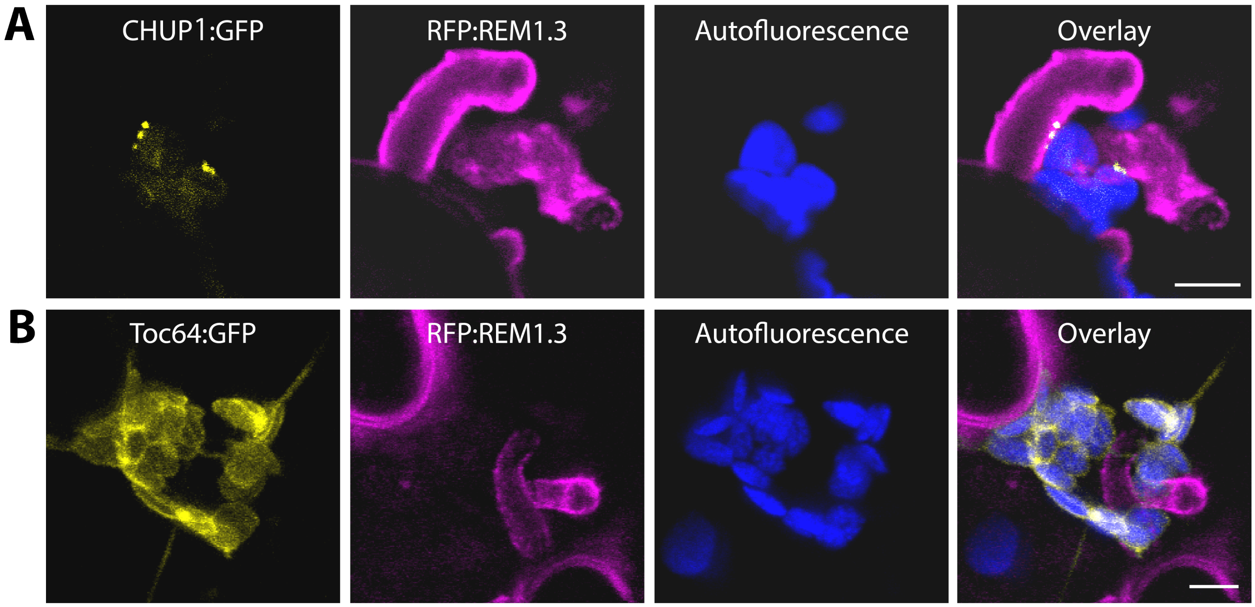

Supplementary Figure S2. CHUP1 forms punctate accumulation at chloroplast-EHM MCS, but the control Toc64 does not. Additional representative images for Figures 2C and 2D. Confocal micrographs of *N. benthamiana* leaf epidermal cells transiently expressing either (A) CHUP1:GFP, or (B) Toc64:GFP, with RFP:REM1.3. The leaves were infected with WT *P. infestans* spores at 6 hpi. Images were taken at 3 dpi. REM1.3 is used as an EHM marker. Presented images are single plane images. Scale bars represent 5 µm. Supports Figure 2.

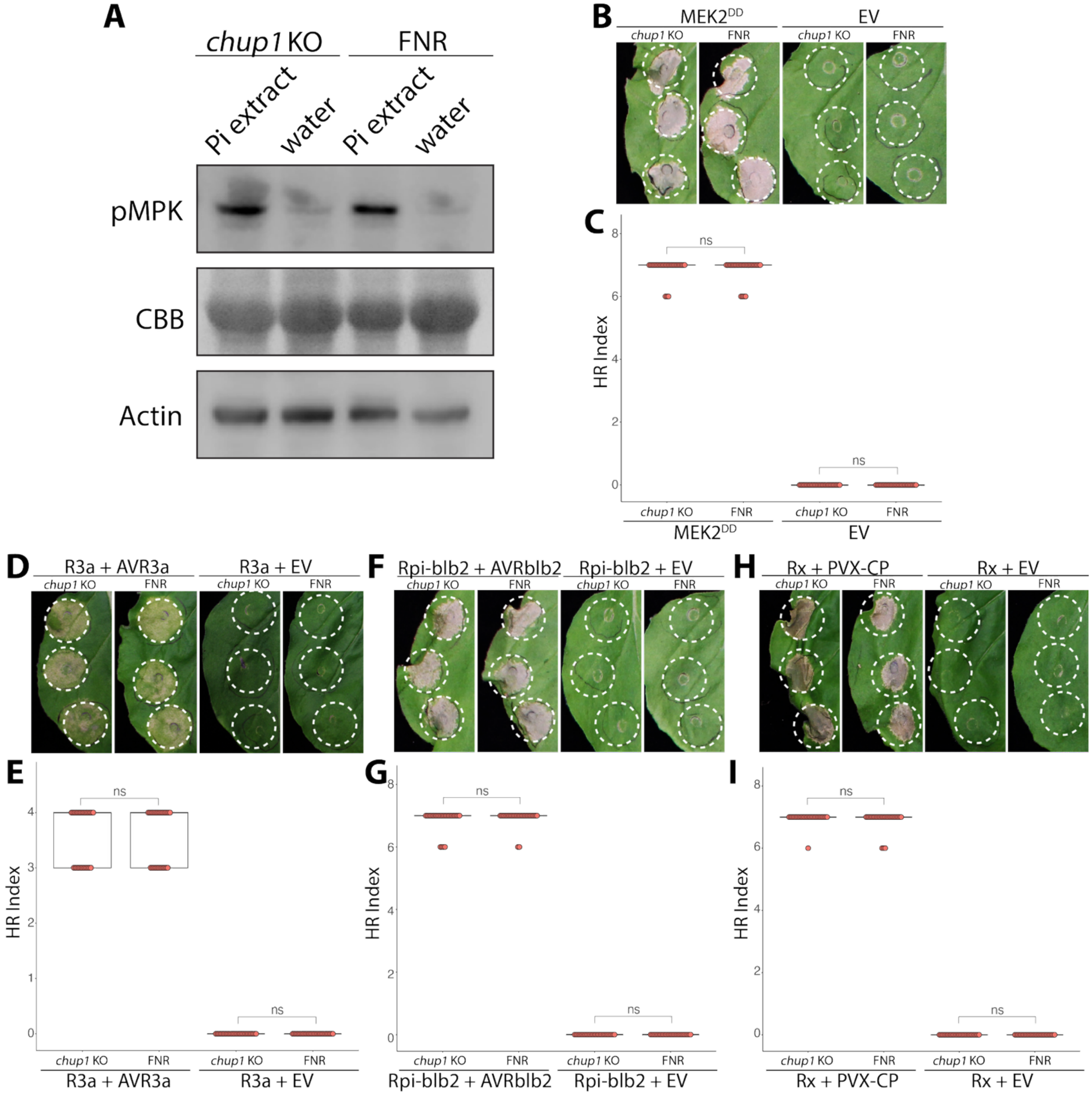

Supplementary Figure S3. The activation of PAMP- or effector-triggered immunity is not impaired in the absence of CHUP1. (A) Western blot analysis of phosphorylated MAPKs in *chup1* KO and FNR plants after a 24-hour treatment with either Pi extract or water. Coomassie brilliant blue staining (CBB) of the gel post protein transfer and actin detection were performed for loading controls. (B-I) For all cell death assays, daylight images were taken, and cell death was scored at 3 dpi. Statistical significance was assessed using Mann-Whitney U test in R. ns = not significant. (B) Representative *chup1* KO and FNR *N. benthamiana* leaves infiltrated with MEK2^{DD} or EV control. (C) Dot plot illustrating that the differences in cell death induced by MEK2^{DD} in *chup1* KO (7, n = 27 spots) and FNR plants (7, n = 27 spots) are not significant. (D) Representative *chup1* KO and FNR *N. benthamiana* leaves infiltrated with R3a and AVR3a or EV

control. (E) Dot plot illustrating that the differences in cell death induced by R3a with AVR3a in *chup1* KO (4, n = 27 spots) and FNR plants (4, n = 27 spots) are not significant. (F) Representative *chup1* KO and FNR *N. benthamiana* leaves infiltrated with Rpi-blb2 and AVRblb2 or EV control. (G) Dot plot illustrating that the differences in cell death induced by Rpi-blb2 with AVRblb2 in *chup1* KO (7, n = 27 spots) and FNR plants (7, n = 27 spots) are not significant. (H) Representative *chup1* KO and FNR *N. benthamiana* leaves infiltrated with Rx and PVX-CP or EV control. (I) Dot plot illustrating that the differences in cell death induced by Rx with PVX-CP in *chup1* KO (7, n = 27 spots) and FNR plants (7, n = 27 spots) are not significant. In the dot plots, the center line denotes the median HR index, and boxes span the interquartile range (25th–75th percentiles). Measurements were not significant (ns) when $p \geq 0.05$. EV: empty vector; KO: knockout; HR: hypersensitive response; Pi: *Phytophthora infestans*. Supports Figure 3.

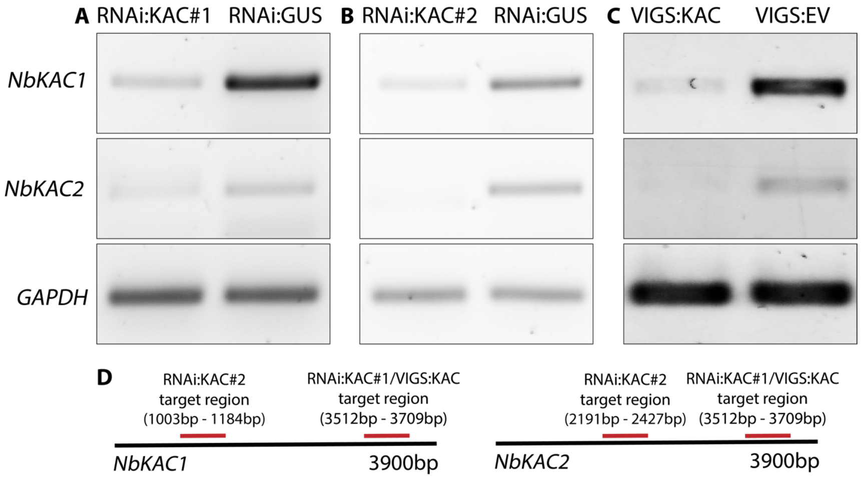

Supplementary Figure S4. Validation of KACs silencing using the RNAi:KAC#1, RNAi:KAC#2 constructs and VIGS:KAC plants. (A-B) Two independent hairpin plasmids (pRNAi-GG) targeting both KAC1 and KAC2 (RNAi:KAC#1 and RNAi:KAC#2) or the GUS reporter gene were infiltrated into *N. benthamiana* leaves. The expression levels of the targeted genes were evaluated via RT-PCR at 3 days post-silencing. (C) Constructs carrying TRV1 with TRV2-GG targeting *NbKAC1* and *NbKAC2* or the control TRV2:EV were infiltrated into *N. benthamiana leaves*, and the expression levels of the targeted genes were assessed via RT-PCR at 3 weeks post-VIGS. RT-PCR analysis utilized primers KAC1_RTPCR_F and KAC1_RTPCR_R for *NbKAC1*, and KAC2_RTPCR_F and KAC2_RTPCR_R for *NbKAC2*. The results confirmed gene silencing of *NbKAC1* and *NbKAC2* using the (A) RNAi:KAC#1 construct, (B) RNAi:KAC#2 construct, and (C) of the VIGS:KAC plants. Glyceraldehyde 3-phosphate dehydrogenase (GAPDH) served as the internal control, using primers GAPDH_RTPCR_F and GAPDH_RTPCR_R for assessment. The cDNA was synthesized from total RNA. (D) Graphical representation of the *NbKAC1* and *NbKAC2* target regions targeted by the RNAi:KAC#1, RNAi:KAC#2 constructs and VIGS:KAC plants. VIGS: virus-induced gene silencing; EV: empty vector. Supports Figure 4.

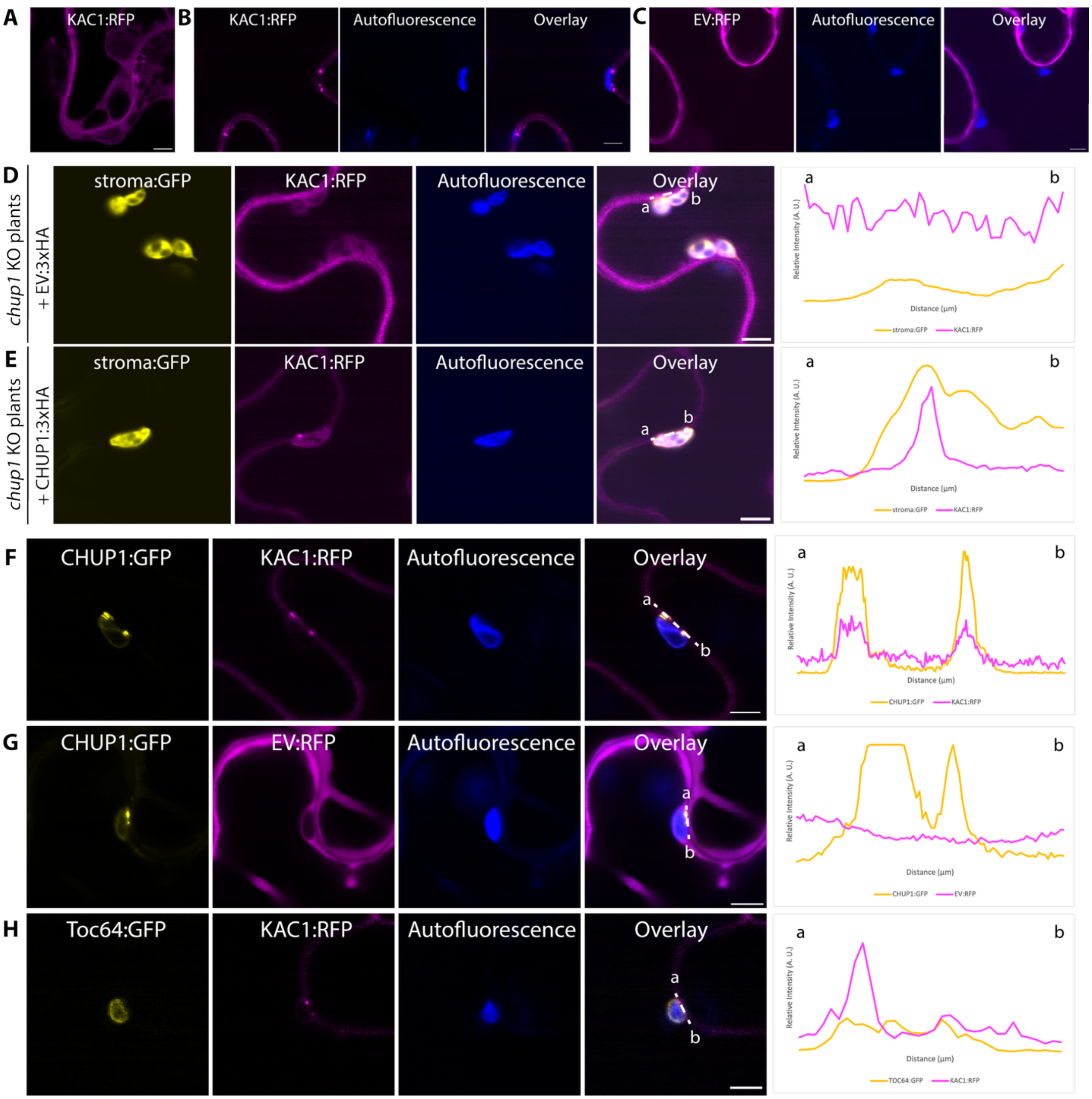

Supplementary Figure S5. KAC1 localizes to PM and requires CHUP1 to accumulate at MCS between chloroplasts and the PM. (A) KAC1:RFP localizes to the PM and cytosol. Confocal micrograph of *N. benthamiana* leaf epidermal cells transiently expressing KAC1:RFP. (B-C) KAC1:RFP forms punctate accumulation at chloroplast-PM MCS, but the control EV:RFP does not. Confocal micrographs of *N. benthamiana* leaf epidermal cells transiently expressing either (B) KAC1:RFP or (C) EV:RFP. (D-E) CHUP1 is required for KAC1 to form punctate structures at chloroplast-PM MCS. Confocal micrographs of *chup1* KO *N. benthamiana* leaf epidermal cells transiently expressing KAC1:RFP with (D) EV:3xHA, or (E) CHUP1:3xHA. GFP channel depicts chloroplast stroma in *chup1* KO plants. (F-H) CHUP1, but not Toc64, colocalizes with KAC1 at punctate structures at chloroplast-PM MCS. Confocal micrographs of *N. benthamiana* leaf epidermal cells transiently expressing (F) CHUP1:GFP and KAC1:RFP, (G)

CHUP1:GFP and EV:RFP, and (H) Toc64:GFP and KAC1:RFP. All presented confocal images are single plane images. Images were taken at 3 dpi. Autofluorescence channel depicts chloroplasts. Transects in overlay panels correspond to line intensity plots depicting the relative fluorescence across the marked distance. Scale bars represent 5 µm. EV: empty vector; KO: knockout; AU: arbitrary unit. Supports Figure 5.

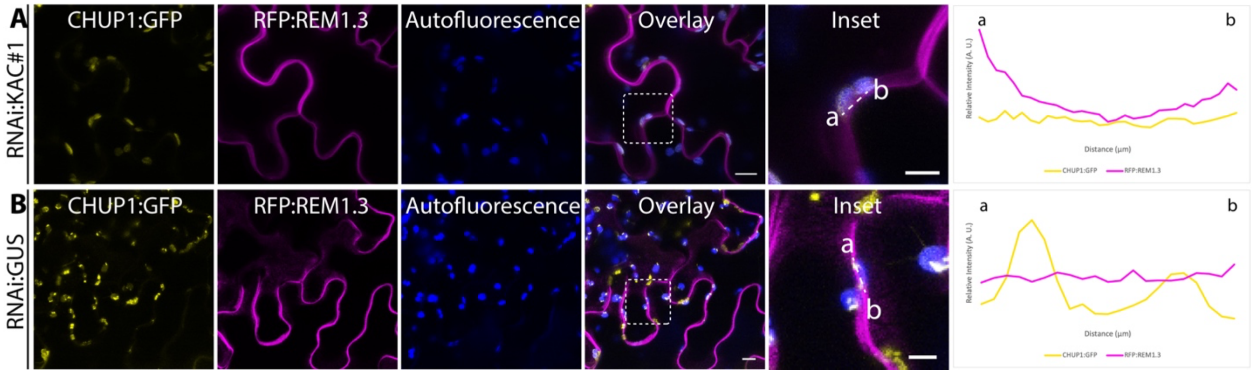

Supplementary Figure S6. Silencing KAC1 reduces CHUP1 punctate accumulation at chloroplast-PM MCS. Confocal micrographs of *N. benthamiana* leaf epidermal cells transiently expressing CHUP1:GFP and RFP:REM1.3, with (A) RNAi:KAC#1 , or (B) RNAi:GUS control. Among chloroplasts adjacent to the PM, KAC1 silencing results in reduced CHUP1 punctate accumulation at chloroplast-PM MCS (13.3%, n = 240 chloroplasts) compared to the GUS-silencing control (81.8%, n = 187 chloroplasts). Scale bars represent 10 µm in overlay panels, and 5 µm in inset panels. RFP:REM1.3 acts as an PM marker. The leaves were imaged at 3 dpi. Presented confocal images are Z-stack images. Autofluorescence channel depicts chloroplasts. Transects in overlay panels correspond to line intensity plots depicting the relative fluorescence across the marked distance. AU: arbitrary unit. Supports Figure 5.

274

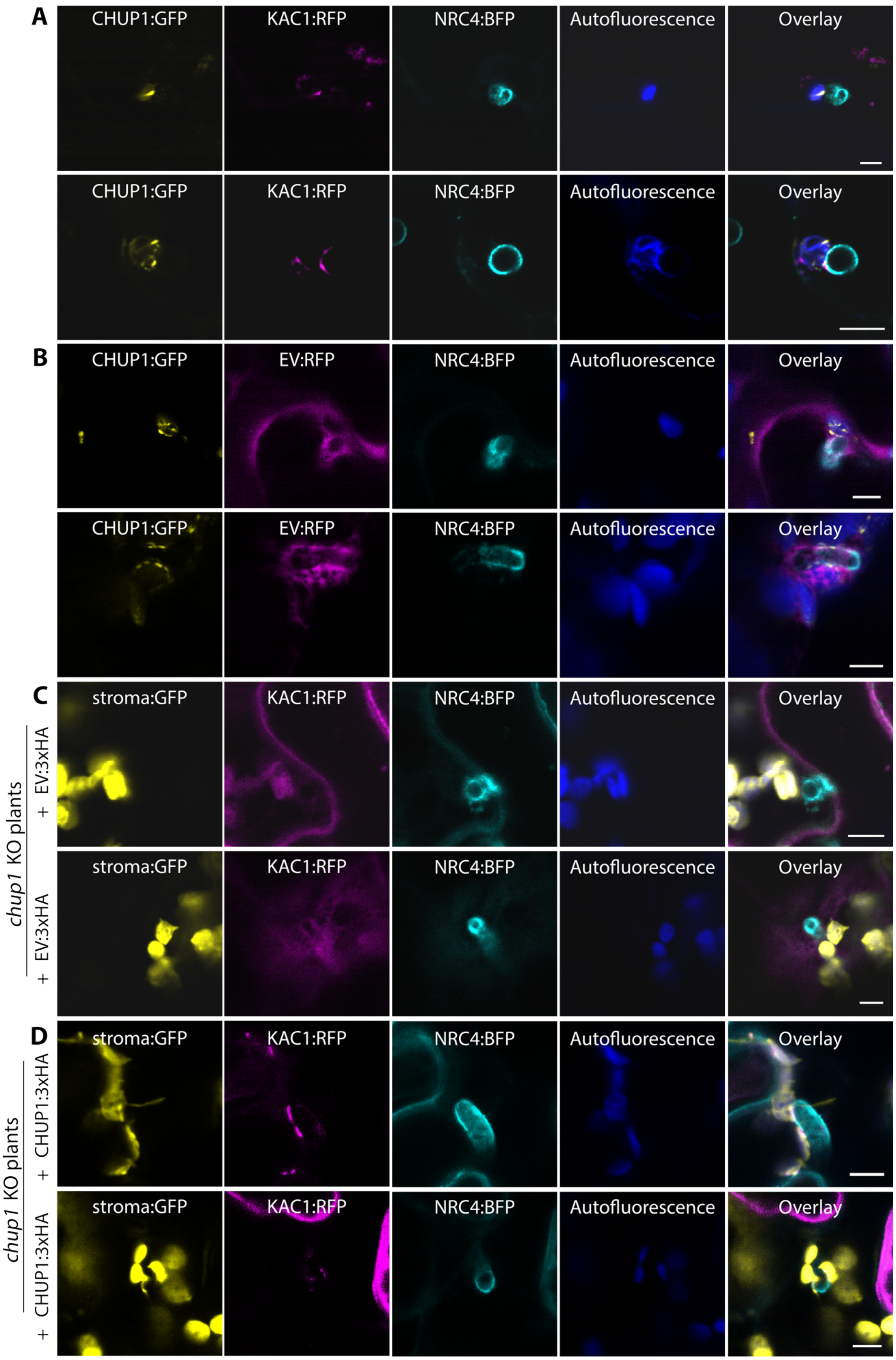
A
B
C
D
CHUP1:GFP
KAC1:RFP
NRC4:BFP
Autofluorescence
Overlay
EV:RFP
stroma:GFP
chup1 KO plants
+ EV:3xHA
+ CHUP1:3xHA

Supplementary Figure S7. KAC1 colocalizes with CHUP1 at chloroplast-EHM MCS. Additional representative images for Figures 5A – 5D. (A-B) Additional representative images for Figures 5A and 5B. CHUP1 colocalizes with KAC1 at punctate structures at chloroplast-EHM MCS. Confocal micrographs of *N. benthamiana* leaf epidermal cells transiently expressing CHUP1:GFP and NRC4:BFP, with (A) KAC1:RFP, or (B) EV:RFP. NRC4:BFP acts as an EHM marker. The leaves were infected with WT *P. infestans* spores at 6 hpi, and imaged at 3 dpi. (C-D) Additional representative images for Figures 5C and 5D. CHUP1 is required for KAC1 to form punctate structures at chloroplast-EHM MCS. Confocal micrographs of *chup1* KO *N. benthamiana* leaf epidermal cells transiently expressing KAC1:RFP and NRC4:BFP with (C) EV:3xHA, or (D) CHUP1:3xHA. NRC4:BFP acts as an EHM marker. The leaves were infected with WT *P. infestans* spores at 6 hpi, and imaged at 3 dpi. All presented confocal images are single plane images. Autofluorescence channel depicts chloroplasts. GFP channel depicts chloroplast stroma in *chup1* KO plants. Scale bars represent 5 μm. EV: empty vector; KO: knockout. Supports Figure 5.

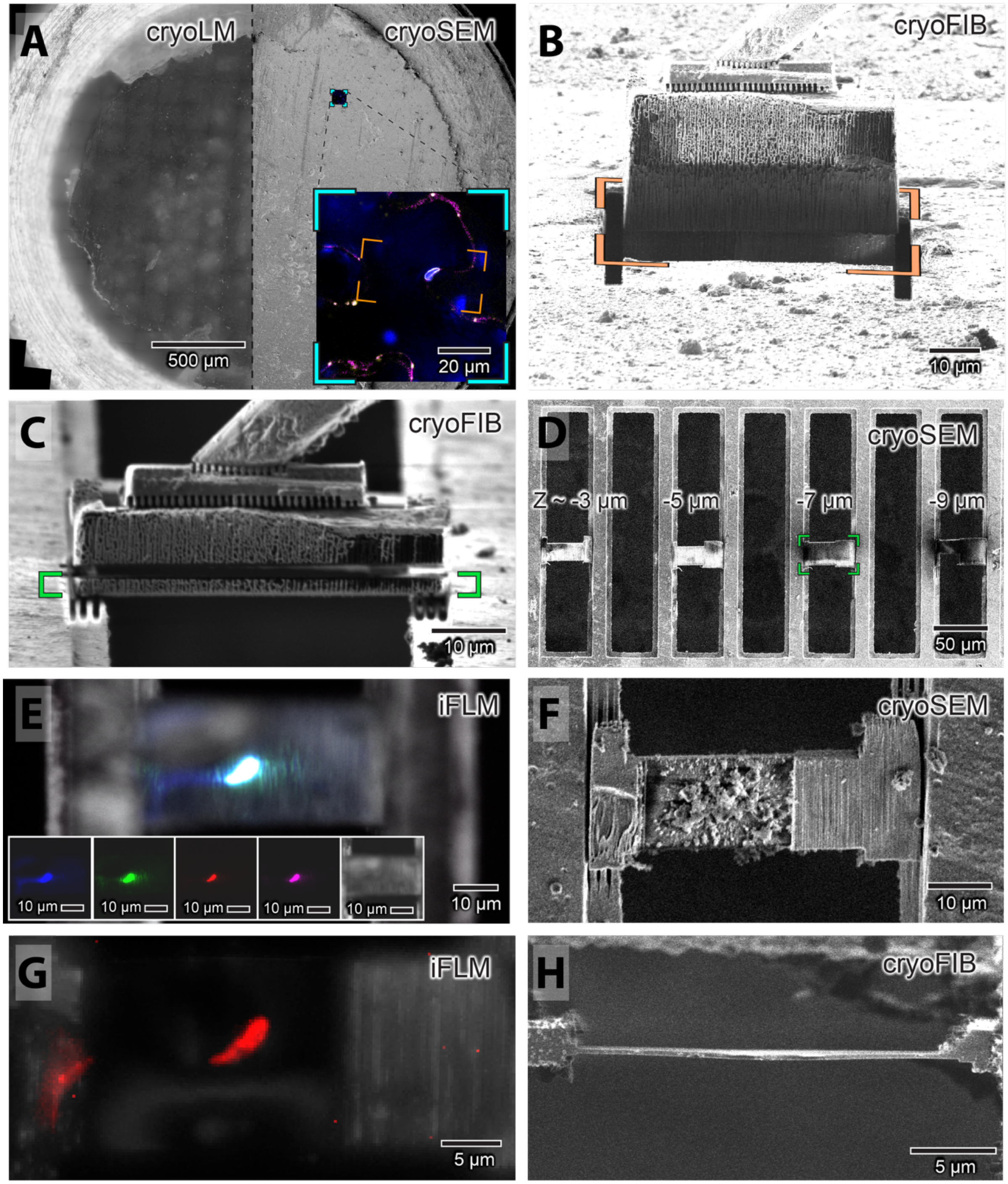

Supplementary Figure S8. Serial lift-out of high-pressure frozen leaves and two-step on-lamella correlative light and electron microscopy. Steps of the workflow are shown. (A) Confocal cryogenic light microscopy (cryoLM) and subsequent correlation with cryogenic scanning electron microscopy (cryoSEM) to locate the chloroplast of interest. The inset shows a single slice through the confocal volume, with the approximate position of the lift-out block (shown in B) highlighted in orange. (B) Cryogenic focused ion beam (cryoFIB) image of a lift-out

block attached to a needle. (C) The block was transferred to an empty copper grid and cut into ~2 µm slices. An in-column fluorescent microscope (iFLM) was used to locate regions of interest within each slice (see E). (D) The slices were trimmed to reduce the length of subsequent thinning steps and then thinned to ~500 nm thickness. (E) While the iFLM can nominally acquire different fluorescent wavelengths, the resulting channels contain nearly identical signals, with chlorophyll fluorescence overpowering almost all other signals. However, it is still useful for verifying which blocks contain the fluorescent signal of interest. The inset shows individual acquisition channels (385 nm, 470 nm, 565 nm, 625 nm, and reflected light). (F) To acquire single-channel fluorescence data (see Figure S9), the specimen was transferred to a cryo-confocal microscope. However, the Leica cryogenic transfer system causes significant ice contamination, particularly on the lamella surfaces. To mitigate this, the grid was transferred back into the cryoFIB-SEM, where the ice contamination was milled away, and the lamella was further thinned to its final thickness (50–200 nm). Although this removes ice crystals from the lamella, the two-step correlation process results in lamellae with higher overall ice contamination, as ice buildup on other parts of the grid cannot be completely removed. (G) The in-line fluorescent microscope was used to confirm the presence of fluorescent features of interest. (H) Edge-on view of the final polished lamella. Supports Figure 5.

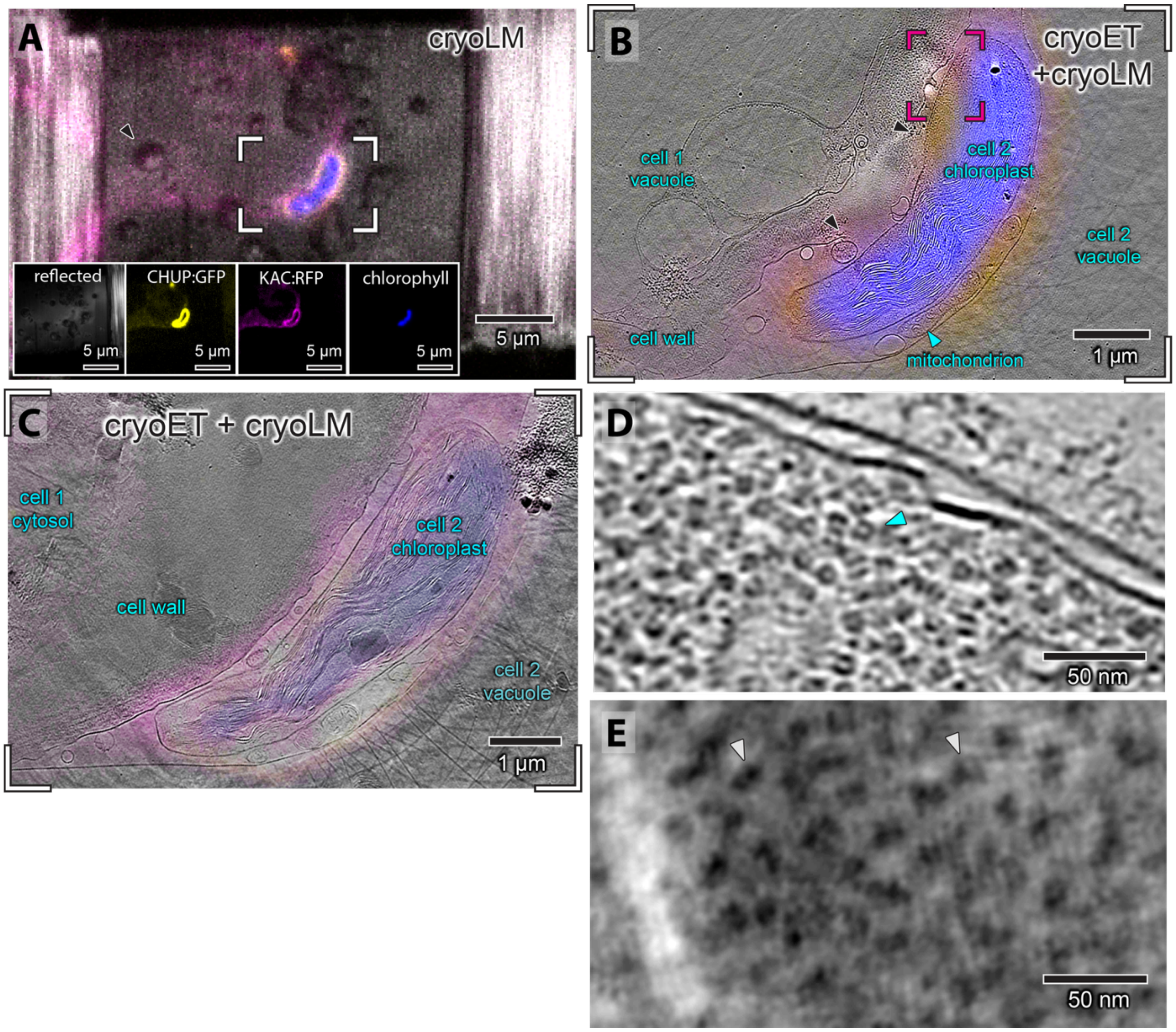

Supplementary Figure S9. Correlative serial lift-out cryoET visualizes molecular detail inside intact vitreous chloroplasts. (A) Confocal cryogenic light microscopy (cryoLM) image of a serial lift-out lamella. The inset shows individual fluorescent channels. Some features observed in the fluorescent signal are caused by crystalline ice on the lamella surface. (B) Slice through a low-magnification tomogram overlaid with the fluorescent data shown in panel A (CHUP1:GFP in yellow, KAC1:RFP in magenta, and native chloroplast fluorescence in blue). The magenta box highlights the location of the tomogram shown in Figure 5I. Black arrowheads indicate the two ends of a ruptured PM, likely resulting from compression during specimen preparation for high-pressure freezing. Serial lift-out slices above and below (panel C) and other observed areas showed no evidence of lysis. Bragg reflections are visible in the data due to incomplete vitrification. (c) Low-magnification overview of a physical slice (same as Figure 5G) located ~2 µm below (towards the upper cuticle), overlaid with correlated cryoLM. (D-E) Molecular-level details observed in Figure 5I, including (D) individual RuBisCO complexes (indicated by blue arrow) and (E) photosystem components on the thylakoid membranes (indicated by white arrows). Supports Figure 5.

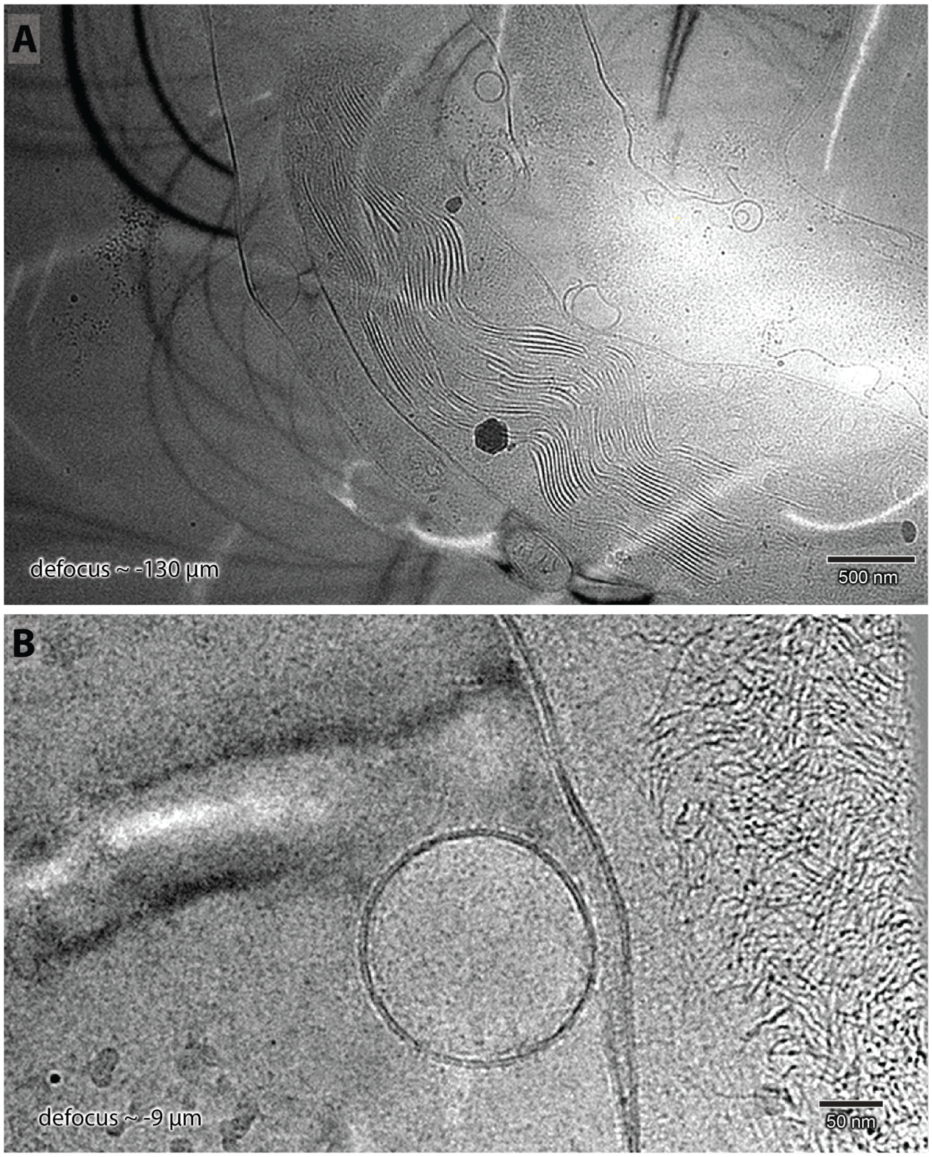

Supplementary Figure S10. Incomplete vitrification observed in the high-pressure frozen specimen. (A) Low-magnification projection image showing abundant Bragg reflections caused by crystalline ice within the lamella. (B) Single projection image from a tomogram tilt series, showing crystalline water reflections in the cytosol. While the vacuolar content and cytosol were crystalline, the lumen of chloroplasts, mitochondria, and the cell wall remained vitreous, as determined from the acquired tilt series. Suboptimal vitrification can lead to specimen damage and introduce artifacts that may result in erroneous interpretation of observed densities. However, we have included these data for the following reasons: 1) Structural preservation is acceptable, as indicated by the integrity of membranes. Specimens exhibiting signs of solute segregation artifacts were excluded; 2) We refrain from drawing conclusions from features that could plausibly be affected by poor vitrification. Supports Figure 5.

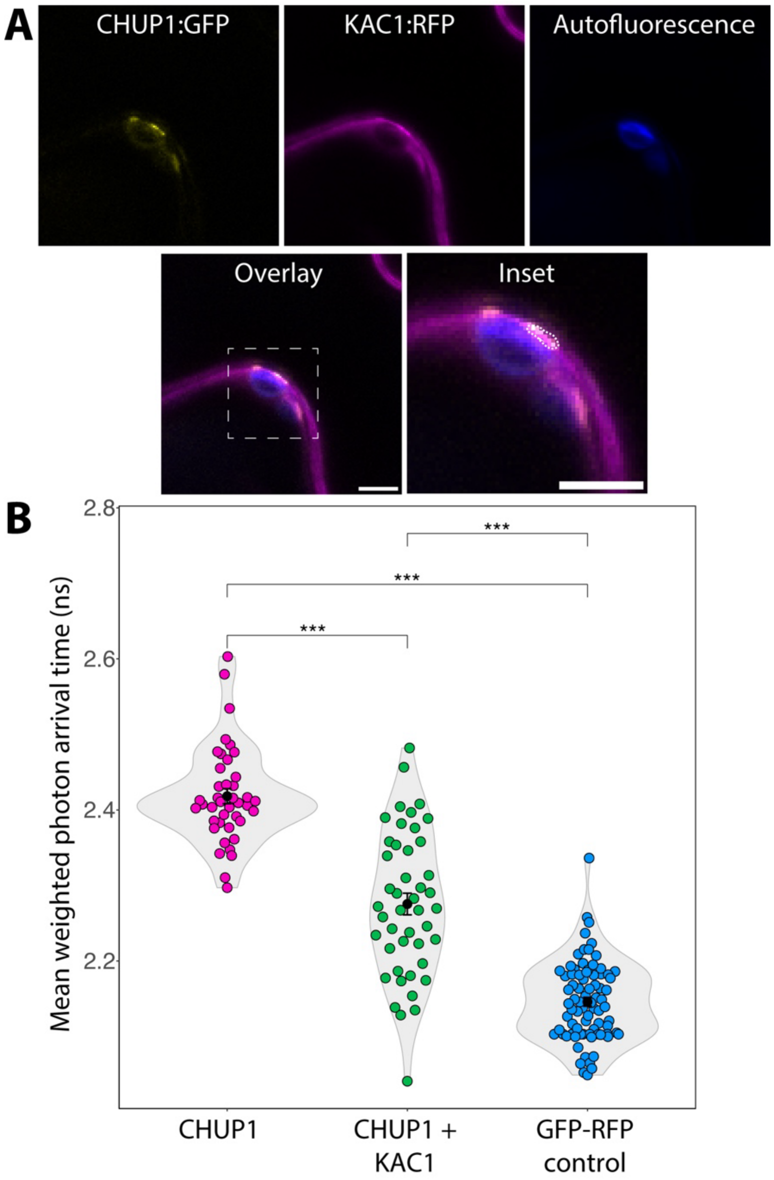

Supplementary Figure S11. Non-fitting FLIM-FRET analysis shows CHUP1 and KAC1 interact *in planta*. (A) Confocal micrographs of *N. benthamiana* leaf epidermal cells transiently co-expressing CHUP1:GFP and KAC1:RFP. The dashed square in the overlay panel corresponds to the zoomed inset. The dashed region in the inset indicates an example of a segmented region with high signal accumulation, used for FRET analysis. (B) Violin plot of mean weighted photon arrival time under three conditions: CHUP1:GFP (donor only), CHUP1:GFP + KAC1:RFP, and GFP-RFP:Rab8a (positive FRET control). Co-expression of CHUP1:GFP + KAC1:RFP resulted in a significantly reduced mean photon arrival time (2.275 ns, n = 45 regions), compared to CHUP1:GFP donor-only controls (2.418 ns, n = 41 regions). The positive control GFP-RFP:Rab8a exhibited an even lower arrival time (2.146 ns, n = 79 regions), confirming assay performance. Statistical differences were analyzed by Mann-Whitney U test in R. Error bars represent the mean ± standard error of the mean (SE). Measurements were highly significant when $p < 0.001$ (***). Supports Figure 5.

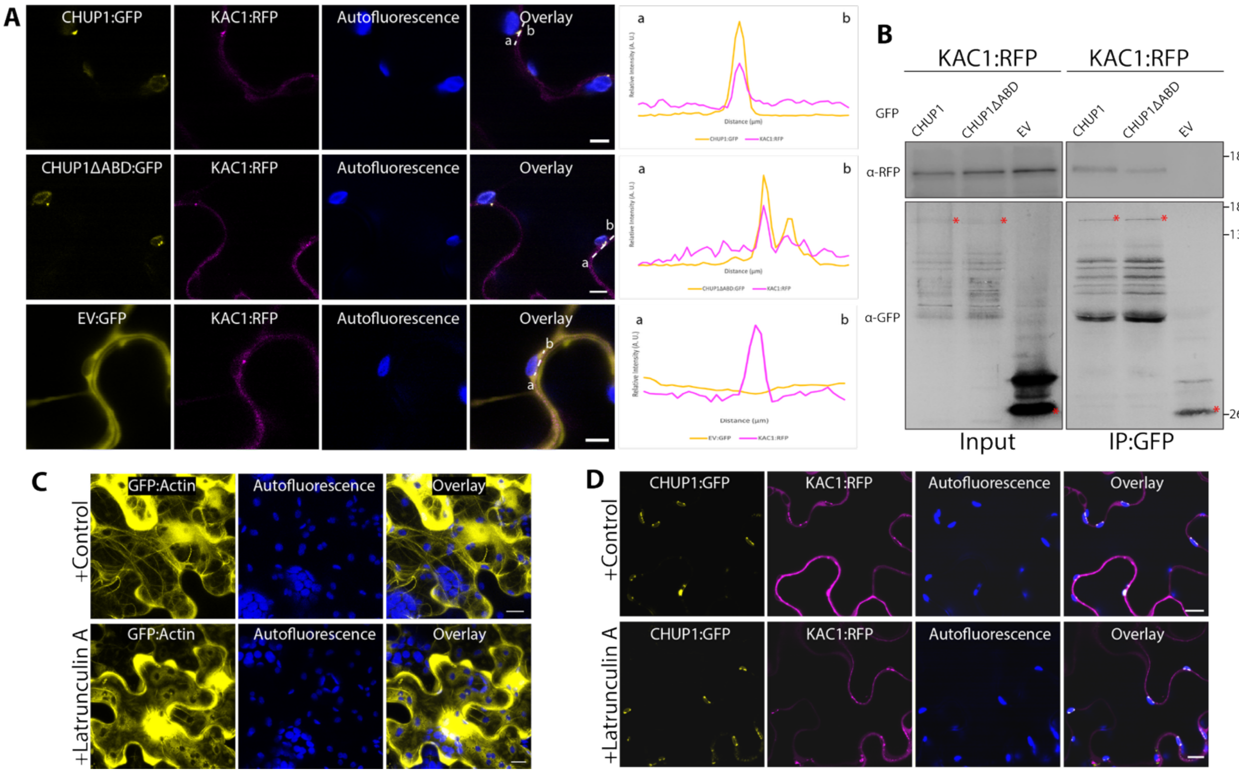

Supplementary Figure S12. CHUP1 colocalizes and interacts with KAC1 independent of the actin-binding domain of CHUP1 and under latrunculin A treatment. (A) CHUP1 colocalizes with KAC1 at punctate structures at chloroplast-PM MCS independent of its actin-binding domain. Confocal micrographs of *N. benthamiana* leaf epidermal cells transiently expressing CHUP1:GFP and KAC1:RFP (1st row), CHUP1ΔABD:GFP and KAC1:RFP (2nd row), and EV:GFP and KAC1:RFP (3rd row). All presented confocal images are single plane images. Images were taken at 3 dpi. Autofluorescence channel depicts chloroplasts. Transects in overlay panels correspond to line intensity plots depicting the relative fluorescence across the marked distance. Scale bars represent 5 µm. (B) CHUP1 interacts with KAC1 independent of its actin-binding domain. KAC1:RFP was transiently co-expressed with either CHUP1:GFP, CHUP1ΔABD:GFP, or EV:GFP. IPs were obtained with anti-GFP antibody. Total protein extracts were immunoblotted. Red asterisks indicate band sizes. Numbers on the right indicate kDa values. (C) Validation of actin disruption by latrunculin A *in planta*. Confocal micrographs of *N. benthamiana* leaf epidermal cells transiently expressing Actin:GFP, treated for 24 hours with either water control or 1.5 µM latrunculin A. Images shown are maximum projection of z-stack images. Images were taken at 3 dpi. Scale bars represent 10 µm. (D) CHUP1 colocalizes with KAC1 at punctate structures at chloroplast-PM MCS under latrunculin A treatment. Confocal micrographs of *N. benthamiana* leaf epidermal cells transiently expressing CHUP1:GFP and KAC1:RFP, treated for 24 hours with either water control (84.6%, n = 39 chloroplasts) or 1.5 µM latrunculin A (86.8%, n = 53 chloroplasts). Images shown are single plane images. Images were taken at 3 dpi. Scale bars represent 10 µm. ABD: actin-binding domain; KO: knockout; AU: arbitrary unit. Supports Figure 5.

Supplementary Table S1. List of primers used in this study.

Primer	Sequence
CHUP1_F	CAGGCGGCCGCACTAGTGATGATCGTCAGGGTAGGTTTAGTGG
CHUP1_R	GCAGATCCAGCAGATCCGATTGTTTCTTGTGTATTCTCTTCTCC
CHUP1_sil_F	AGAATGAAATGGTTGCCC
CHUP1_sil_R	GATCCTGCGTATTCTAACATC
NbCHUP1a_RTPCR_F	ATGATCGTCAGGGTAGGTTTAGTGGTTGC
NbCHUP1a_RTPCR_R	TGTTTCTTGTGTATTCTCTTCTCCTGTTTGT
NbCHUP1b_RTPCR_F	TGATAGTCAGGGTAGGTTTAGTGGTTGC
NbCHUP1b_RTPCR_R	TGTTTCTTGTGTATTCTCTTCTCCTGTTTGA
CHUP1Nterm_R	GCAGATCCAGCAGATCCGATGGGTTTTACATTTATCTGCTTAACTGC
CHUP1KO_genotype_F	AGCTTGATCTTGATGATGATCTTTTC
CHUP1KO_genotype_R	TCTTTGTTCTTCCTCTTAAGCTCCA
CHUP1KO2_genotype_F	GTGTGCTTTAGAAACAGTAATTTAAGTC
CHUP1KO2_genotype_R	TCTTTGTTCTTCCTCTTAAGCTCC
CHUP1ΔABD_Frag1_R	CGAAGTTCAAATCTTAAGCATTCAACTTCACTGAATCTGT
CHUP1ΔABD_Frag2_F	ACAGATTCAGTGAAGTTGAATGCTTAAGATTTGAACTTCG
KAC1_F	CAGGCGGCCGCACTAGTGATATGGCGGAGCAGAAGAGC
KAC1_R	GCAGATCCAGCAGATCCGATTGATTTGACATCCTTCAGCAC
KAC1_sil_F	ACCAGGTCTCAGGAGGATTAGGGGGTGAAACTGAC
KAC1_sil_R	ACCAGGTCTCATCGTAGCTCATATTTTCAAGAACG
KAC1_RTPCR_F	GCTCTAGCCGTGGTAGTAGC
KAC1_RTPCR_R	AACAGATTCTAAAGCTGAACGTAGC
KAC2_RTPCR_F	GATATCGTTCCATATGCAAATTCAGT
KAC2_RTPCR_R	GTTGAGCTACTTGATTTCTAGTTTGT
GAPDH_RTPCR_F	ATGGCTTCTCATGCAGCTTT
GAPDH_RTPCR_R	ATCCTGTGGTCTTGGGAGTG
GFPRFP_Rab8a_F	CTGGATCTGGAGAATTTGATATGGCCTCCTCCGAGGACG

GFPRFP_Rab8a_R	TAGCATGGCCGCGGGATTTAAGAACCACAGCAAGCTGATTTTTG
NLS	GGCGGCCGCACTAGTGATGCCTAAAAAGAAGCGTAAGGTTGAGGACCCTGATATCGGATCTGCTGGATCT

Supplementary Table S2. List of synthetic fragments used in this study.

**Synthetic fragment**	**Sequence**
RNAi:KAC#2	ATTAGGTCTCAGGAGGATACGTCAAAGTTTAGAGTTTCTCATCTAATCATCACAGTACATATACATTATACCAACTTGATCACTGGAGAGACTTCATACAGCAAGCTCTCTCTTGTTGATTTGGCTGTAAGTGAAAGTACTGTTGAAGAGGATAGGGGGGAGCATGCAACAGAGTTGCTTCATGTCATGAAATCACTGCTGGCGAATATCTTACTTCTGCATTGAACGAATTTGATCCTGATCAATTTGATAGTCTTGCTGCAATCTCTGATGGAGCAAACAAGCTTTTGATGCTGGTTTTGGCAGCAGTCATCAAAGCAGGTGCCTCTAGGGAGCATGAAATACTTGCTGAAATTCGAGATGCCGTCTTTGCTTTCATTCGTAAAATGGAGCCAAAGAGAGTGATGGATACCATGCTTGTTTCCCGTGTTAGGACGATGAGACCTAAT
MEK2^{DD}	CAGGCGGCCGCACTAGTGATGCGACCTCTTCAACCACCCCCACCAGCCGCCGCCGCCACCACTACCTCCTCTTCCACCACCGCATCACCCATGCCCCCTCCTCCTTCACGCAACCGTCCCCGTCGTCGTACCAATTTAACTCTCCCCCTTCCCCAACGTGACCCAGCTCTCGCCGTACCCCTCCCCCTCCCCCCTACTTCCGCCCCTTCTTCCTCGTCGTCTTCCTCTTCCTCCCCACTCCCCACCCCCTTAAACTTCTCCGAACTTGAGCGCATCAATCGCATCGGCAGCGGCGCTGGCGGTACGGTTTACAAAGTCCTATATCGCCCCACCGGAAGACTCTACGCTCTCAAAGTCATCTACGGTAACCACGAGGACTCCGTTCGCCTTCAGATGTGCCGTGAGATCGAGATTCTCCGTGACGTCGACAACCCTAACGTCGTTAGATGTCACGATATGTTCGATCACAACGGTGAAATCCAAGTCCTCCTTGAATTCATGGATAAAGGCTCTCTTGAAGGGATCCACATCCCTAAAGAGTCAGCTCTTTCGGATCTAACCCGACAAGTCCTCTCGGGACTCTATTATCTCCACAGGCGTAAGATTGTGCACAGAGATATCAAGCCCTCGAATTTACTAATCAACTCGAGGCGTGAGGTGAAAATTGCTGACTTTGGGGTGTCGAGAGTGCTGGCACAAGACATGGATCCTTGTAATGATTCAGTTGGGACAATTGCCTATATGAGTCCAGAGAGAATCAACACAGATCTGAATCATGGACAGTACGATGGGTATGCTGGAGATATATGGAGTCTTGGTGTTAGCATATTGGAGTTTTATTTGGGAAGGTTTCCGTTTTCTGTTGGGAGGTCAGGTGATTGGGCTAGTCTTATGTGCGCCATTTGTATGTCGCAGCCGCCGGAGGCTCCGGCGAATGCTTCTAGAGAGTTCAGGGACTTTATTGCTTGCTGTTTGCAGAGGGATCCTGCGCGGCGGTGGACGGCGGTGCAGCTGTTGCGTCATCCATTTATTACCCAGAATAGACCAGCCACTACCACCACCGGTAATATGATGCCACTTCCTAATCAAGTTCAT

	CAGCCAGCACATCAATTGTTACCCCCGCCTCATTTTTCTTC TATCGGATCTGCTGGATCTGC

Supplementary Table S3. List of constructs used in this study.

Construct	Reference
Vector (pK7WGF2 domesticated for Gibson assembly)	(Dagdas et al., 2018)
Vector (pRNAI-GG)	(Yan et al., 2012)
Vector (TRV2-GG)	(Duggan et al., 2021)
TRV1	(Ratcliff et al., 2001)
TRV2:EV	(Duggan et al., 2021)
TRV2:KAC	This work (primers: KAC1_sil_F and KAC1_sil_R)
TRV2:CHUP1	This work (primers: CHUP1_sil_F and CHUP1_sil_R)
RNAi:KAC#1	This work (primers: KAC1_sil_F and KAC1_sil_R)
RNAi:KAC#2	This work (synthetic fragment: RNAi:KAC#2)
RNAi:GUS	(Yuen et al., 2024b)
KAC1:RFP	This work (primers: KAC1_F and KAC1_R)
CHUP1:GFP	This work (primers: CHUP1_F and CHUP1_R)
CHUP1:3xHA	This work (primers: CHUP1_F and CHUP1_R)
CHUP1^{Nterm}:GFP	This work (primers: CHUP1_F and CHUP1Nterm_R)
CHUP1ΔABD:GFP	This work (primers: CHUP1_F and CHUP1ΔABD_Frag1_R, CHUP1ΔABD_Frag2_F and CHUP1_R)
Toc64:GFP	(Breuers et al., 2012)
RFP:REM1.3	(Bozkurt et al., 2014)
NRC4:BFP	(Duggan et al., 2021)
GFP:Actin	(Rocchetti et al., 2014; Savage et al., 2021)
EV:GFP	(Duggan et al., 2021)
EV:RFP	(Dagdas et al., 2016)
3xHA:EV	(Pandey et al., 2021)
GFP:RFP:Rab8a	This work (primers: GFPRFP_Rab8a_F and GFPRFP_Rab8a_R)
NLS:BFP	This work (primer: NLS)
MEK2^{DD}:3xHA	This work (synthetic fragment: MEK2^{DD})

AVR3a	Provided by TSLSynBio
R3a	(Chaparro-Garcia et al., 2015)
GFP:AVRblb2	(Bozkurt et al., 2011)
RFP:Rpi-blb2	(Bozkurt et al., 2011)
PVX-CP	(Contreras et al., 2023c)
Rx	(Contreras et al., 2023c)

Supplementary Table S4. List of antibodies used in this study.

Primary/Secondary	Detail	Company	Product Code
Primary	Anti-GFP (Rat monoclonal)	Chromotek	3H9
Primary	Anti-RFP (Mouse monoclonal)	Chromotek	6G6
Primary	Anti-phospho-MAPK (Rabbit polyclonal)	Cell Signaling Technology	9101
Secondary	Anti-rabbit (HRP)	Sigma-Aldrich	A9169
Secondary	Anti-rat (HRP)	Sigma-Aldrich	A9037
Secondary	Anti-mouse (HRP)	Sigma-Aldrich	SAB3700986
Primary/Secondary	HRP-conjugated anti-beta actin (Mouse monoclonal)	Proteintech	HRP-60008

References

Agaisse, H., and Derre, I. (2014). Expression of the effector protein IncD in Chlamydia trachomatis mediates recruitment of the lipid transfer protein CERT and the endoplasmic reticulum-resident protein VAPB to the inclusion membrane. Infect Immun **82,** 2037-2047.

Almagro, L., Gomez Ros, L.V., Belchi-Navarro, S., Bru, R., Ros Barcelo, A., and Pedreno, M.A. (2009). Class III peroxidases in plant defence reactions. J Exp Bot **60,** 377-390.

Auer, J.M.T., Murphy, L.C., Xiao, D., Li, D.U., and Wheeler, A.P. (2023). Non-fitting FLIM-FRET facilitates analysis of protein interactions in live zebrafish embryos. J Microsc **291,** 43-56.

Bendahmane, A., Kohn, B.A., Dedi, C., and Baulcombe, D.C. (1995). The coat protein of potato virus X is a strain-specific elicitor of Rx1-mediated virus resistance in potato. Plant J **8,** 933-941.

Bos, J.I., Kanneganti, T.D., Young, C., Cakir, C., Huitema, E., Win, J., Armstrong, M.R., Birch, P.R., and Kamoun, S. (2006). The C-terminal half of Phytophthora infestans RXLR effector AVR3a is sufficient to trigger R3a-mediated hypersensitivity and suppress INF1-induced cell death in Nicotiana benthamiana. Plant J **48,** 165-176.

Bozkurt, T.O., and Kamoun, S. (2020). The plant-pathogen haustorial interface at a glance. J Cell Sci **133.**

Bozkurt, T.O., Richardson, A., Dagdas, Y.F., Mongrand, S., Kamoun, S., and Raffaele, S. (2014). The Plant Membrane-Associated REMORIN1.3 Accumulates in Discrete Perihaustorial Domains and Enhances Susceptibility to Phytophthora infestans. Plant Physiol **165,** 1005-1018.

Bozkurt, T.O., Schornack, S., Win, J., Shindo, T., Ilyas, M., Oliva, R., Cano, L.M., Jones, A.M., Huitema, E., van der Hoorn, R.A., and Kamoun, S. (2011). Phytophthora infestans effector AVRblb2 prevents secretion of a plant immune protease at the haustorial interface. Proc Natl Acad Sci U S A **108,** 20832-20837.

Breuers, F.K., Brautigam, A., Geimer, S., Welzel, U.Y., Stefano, G., Renna, L., Brandizzi, F., and Weber, A.P. (2012). Dynamic Remodeling of the Plastid Envelope Membranes - A Tool for Chloroplast Envelope in vivo Localizations. Front Plant Sci **3,** 7.

Caplan, J.L., Kumar, A.S., Park, E., Padmanabhan, M.S., Hoban, K., Modla, S., Czymmek, K., and Dinesh-Kumar, S.P. (2015). Chloroplast Stromules Function during Innate Immunity. Dev Cell **34,** 45-57.

Castro, B., Citterico, M., Kimura, S., Stevens, D.M., Wrzaczek, M., and Coaker, G. (2021). Stress-induced reactive oxygen species compartmentalization, perception and signalling. Nat Plants **7,** 403-412.

Chaparro-Garcia, A., Schwizer, S., Sklenar, J., Yoshida, K., Petre, B., Bos, J.I., Schornack, S., Jones, A.M., Bozkurt, T.O., and Kamoun, S. (2015). Phytophthora infestans RXLR-WY Effector AVR3a Associates with Dynamin-Related Protein 2 Required for Endocytosis of the Plant Pattern Recognition Receptor FLS2. PLoS One **10,** e0137071.

Cheval, C., Samwald, S., Johnston, M.G., de Keijzer, J., Breakspear, A., Liu, X., Bellandi, A., Kadota, Y., Zipfel, C., and Faulkner, C. (2020). Chitin perception in plasmodesmata characterizes submembrane immune-signaling specificity in plants. Proc Natl Acad Sci U S A **117,** 9621-9629.

Chung, J., Torta, F., Masai, K., Lucast, L., Czapla, H., Tanner, L.B., Narayanaswamy, P., Wenk, M.R., Nakatsu, F., and De Camilli, P. (2015). INTRACELLULAR

TRANSPORT. PI4P/phosphatidylserine countertransport at ORP5- and ORP8-mediated ER-plasma membrane contacts. Science **349,** 428-432.
Chung, K.K., Zhao, Z., Law, K.C., Ma, J., Chiang, C.H., Leung, K.H., Shrestha, R., Wu, Y., Li, C., Lee, K.M., Feng, L., Li, X., Wong, K.B., Xu, S.L., Gao, C., and Zhuang, X. (2024). Biomolecular condensation of ERC1 recruits ATG8 and NBR1 to drive autophagosome formation for plant heat tolerance. bioRxiv.
Contreras, M.P., Ludke, D., Pai, H., Toghani, A., and Kamoun, S. (2023a). NLR receptors in plant immunity: making sense of the alphabet soup. EMBO Rep **24,** e57495.
Contreras, M.P., Pai, H., Tumtas, Y., Duggan, C., Yuen, E.L.H., Cruces, A.V., Kourelis, J., Ahn, H.K., Lee, K.T., Wu, C.H., Bozkurt, T.O., Derevnina, L., and Kamoun, S. (2023b). Sensor NLR immune proteins activate oligomerization of their NRC helpers in response to plant pathogens. EMBO J **42,** e111519.
Contreras, M.P., Pai, H., Selvaraj, M., Toghani, A., Lawson, D.M., Tumtas, Y., Duggan, C., Yuen, E.L.H., Stevenson, C.E.M., Harant, A., Maqbool, A., Wu, C.H., Bozkurt, T.O., Kamoun, S., and Derevnina, L. (2023c). Resurrection of plant disease resistance proteins via helper NLR bioengineering. Sci Adv **9,** eadg3861.
Dagdas, Y.F., Pandey, P., Tumtas, Y., Sanguankiattichai, N., Belhaj, K., Duggan, C., Leary, A.Y., Segretin, M.E., Contreras, M.P., Savage, Z., Khandare, V.S., Kamoun, S., and Bozkurt, T.O. (2018). Host autophagy machinery is diverted to the pathogen interface to mediate focal defense responses against the Irish potato famine pathogen. Elife **7**.
Dagdas, Y.F., Belhaj, K., Maqbool, A., Chaparro-Garcia, A., Pandey, P., Petre, B., Tabassum, N., Cruz-Mireles, N., Hughes, R.K., Sklenar, J., Win, J., Menke, F., Findlay, K., Banfield, M.J., Kamoun, S., and Bozkurt, T.O. (2016). An effector of the Irish potato famine pathogen antagonizes a host autophagy cargo receptor. Elife **5**.
de Torres Zabala, M., Littlejohn, G., Jayaraman, S., Studholme, D., Bailey, T., Lawson, T., Tillich, M., Licht, D., Bolter, B., Delfino, L., Truman, W., Mansfield, J., Smirnoff, N., and Grant, M. (2015). Chloroplasts play a central role in plant defence and are targeted by pathogen effectors. Nat Plants **1,** 15074.
Ding, X., Jimenez-Gongora, T., Krenz, B., and Lozano-Duran, R. (2019). Chloroplast clustering around the nucleus is a general response to pathogen perception in Nicotiana benthamiana. Mol Plant Pathol **20,** 1298-1306.
Duggan, C., Moratto, E., Savage, Z., Hamilton, E., Adachi, H., Wu, C.H., Leary, A.Y., Tumtas, Y., Rothery, S.M., Maqbool, A., Nohut, S., Martin, T.R., Kamoun, S., and Bozkurt, T.O. (2021). Dynamic localization of a helper NLR at the plant-pathogen interface underpins pathogen recognition. Proc Natl Acad Sci U S A **118**.
Eisenstein, F., Yanagisawa, H., Kashihara, H., Kikkawa, M., Tsukita, S., and Danev, R. (2023). Parallel cryo electron tomography on in situ lamellae. Nat Methods **20,** 131-138.
Erickson, J.L., Kantek, M., and Schattat, M.H. (2017). Plastid-Nucleus Distance Alters the Behavior of Stromules. Front Plant Sci **8,** 1135.
Forster, T. (1946). Energiewanderung und Fluoreszenz. Naturwissenschaften **33,** 166-175.
Fuchs, R., Kopischke, M., Klapprodt, C., Hause, G., Meyer, A.J., Schwarzlander, M., Fricker, M.D., and Lipka, V. (2016). Immobilized Subpopulations of Leaf Epidermal Mitochondria Mediate PENETRATION2-Dependent Pathogen Entry Control in Arabidopsis. Plant Cell **28,** 130-145.
Gao, C., Xu, H., Huang, J., Sun, B., Zhang, F., Savage, Z., Duggan, C., Yan, T., Wu, C.H., Wang, Y., Vleeshouwers, V., Kamoun, S., Bozkurt, T.O., and Dong, S. (2020).

Pathogen manipulation of chloroplast function triggers a light-dependent immune recognition. Proc Natl Acad Sci U S A **117,** 9613-9620.
Hagen, W.J.H., Wan, W., and Briggs, J.A.G. (2017). Implementation of a cryo-electron tomography tilt-scheme optimized for high resolution subtomogram averaging. J Struct Biol **197,** 191-198.
Heath, M.C., Nimchuk, Z.L., and Xu, H. (1997). Plant nuclear migrations as indicators of critical interactions between resistant or susceptible cowpea epidermal cells and invasion hyphae of the cowpea rust fungus. New Phytologist **135,** 689-700.
Hubber, A., Arasaki, K., Nakatsu, F., Hardiman, C., Lambright, D., De Camilli, P., Nagai, H., and Roy, C.R. (2014). The machinery at endoplasmic reticulum-plasma membrane contact sites contributes to spatial regulation of multiple Legionella effector proteins. PLoS Pathog **10,** e1004222.
Ibrahim, T., Yuen, E.L.H., Wang, H.-Y., King, F.J., Toghani, A., Kourelis, J., Vuolo, C., Adamkova, V., Castel, B., Jones, J.D., Wu, C.-H., Kamoun, S., and Bozkurt, T.O. (2024). A helper NLR targets organellar membranes to trigger immunity. bioRxiv, 2024.2009.2019.613839.
Irieda, H., and Takano, Y. (2021). Epidermal chloroplasts are defense-related motile organelles equipped with plant immune components. Nat Commun **12,** 2739.
Jelenska, J., van Hal, J.A., and Greenberg, J.T. (2010). Pseudomonas syringae hijacks plant stress chaperone machinery for virulence. Proc Natl Acad Sci U S A **107,** 13177-13182.
Jelenska, J., Yao, N., Vinatzer, B.A., Wright, C.M., Brodsky, J.L., and Greenberg, J.T. (2007). A J domain virulence effector of Pseudomonas syringae remodels host chloroplasts and suppresses defenses. Curr Biol **17,** 499-508.
Jeong, R.D., Kachroo, A., and Kachroo, P. (2010). Blue light photoreceptors are required for the stability and function of a resistance protein mediating viral defense in Arabidopsis. Plant Signal Behav **5,** 1504-1509.
Jung, S., Woo, J., and Park, E. (2024). Talk to your neighbors in an emergency: Stromule-mediated chloroplast-nucleus communication in plant immunity. Curr Opin Plant Biol **79,** 102529.
Kadota, A., Yamada, N., Suetsugu, N., Hirose, M., Saito, C., Shoda, K., Ichikawa, S., Kagawa, T., Nakano, A., and Wada, M. (2009). Short actin-based mechanism for light-directed chloroplast movement in Arabidopsis. Proc Natl Acad Sci U S A **106,** 13106-13111.
King, F.J., Yuen, E.L.H., and Bozkurt, T.O. (2024). Border Control: Manipulation of the Host-Pathogen Interface by Perihaustorial Oomycete Effectors. Mol Plant Microbe Interact **37,** 220-226.
Koh, S., Andre, A., Edwards, H., Ehrhardt, D., and Somerville, S. (2005). Arabidopsis thaliana subcellular responses to compatible Erysiphe cichoracearum infections. Plant J **44,** 516-529.
Kong, S.G., Yamazaki, Y., Shimada, A., Kijima, S.T., Hirose, K., Katoh, K., Ahn, J., Song, H.G., Han, J.W., Higa, T., Takano, A., Nakamura, Y., Suetsugu, N., Kohda, D., Uyeda, T.Q.P., and Wada, M. (2024). CHLOROPLAST UNUSUAL POSITIONING 1 is a plant-specific actin polymerization factor regulating chloroplast movement. Plant Cell **36,** 1159-1181.
Kremer, J.R., Mastronarde, D.N., and McIntosh, J.R. (1996). Computer visualization of three-dimensional image data using IMOD. J Struct Biol **116,** 71-76.

Kwon, C., Neu, C., Pajonk, S., Yun, H.S., Lipka, U., Humphry, M., Bau, S., Straus, M., Kwaaitaal, M., Rampelt, H., El Kasmi, F., Jurgens, G., Parker, J., Panstruga, R., Lipka, V., and Schulze-Lefert, P. (2008). Co-option of a default secretory pathway for plant immune responses. Nature **451,** 835-840.

Lamm, L., Zufferey, S., Righetto, R.D., Wietrzynski, W., Yamauchi, K.A., Burt, A., Liu, Y., Zhang, H., Martinez-Sanchez, A., Ziegler, S., Isensee, F., Schnabel, J.A., Engel, B.D., and Peng, T. (2024). MemBrain v2: an end-to-end tool for the analysis of membranes in cryo-electron tomography. bioRxiv, 2024.2001.2005.574336.

Littlejohn, G.R., Breen, S., Smirnoff, N., and Grant, M. (2021). Chloroplast immunity illuminated. New Phytol **229,** 3088-3107.

Mastronarde, D.N. (1997). Dual-axis tomography: an approach with alignment methods that preserve resolution. J Struct Biol **120,** 343-352.

Medina-Puche, L., Tan, H., Dogra, V., Wu, M., Rosas-Diaz, T., Wang, L., Ding, X., Zhang, D., Fu, X., Kim, C., and Lozano-Duran, R. (2020). A Defense Pathway Linking Plasma Membrane and Chloroplasts and Co-opted by Pathogens. Cell **182,** 1109-1124 e1125.

Micali, C.O., Neumann, U., Grunewald, D., Panstruga, R., and O'Connell, R. (2011). Biogenesis of a specialized plant-fungal interface during host cell internalization of Golovinomyces orontii haustoria. Cell Microbiol **13,** 210-226.

Morton, W.M., Ayscough, K.R., and McLaughlin, P.J. (2000). Latrunculin alters the actin-monomer subunit interface to prevent polymerization. Nat Cell Biol **2,** 376-378.

Naqvi, S., He, Q., Trusch, F., Qiu, H., Pham, J., Sun, Q., Christie, J.M., Gilroy, E.M., and Birch, P.R.J. (2022). Blue-light receptor phototropin 1 suppresses immunity to promote Phytophthora infestans infection. New Phytol **233,** 2282-2293.

Nomura, H., Komori, T., Uemura, S., Kanda, Y., Shimotani, K., Nakai, K., Furuichi, T., Takebayashi, K., Sugimoto, T., Sano, S., Suwastika, I.N., Fukusaki, E., Yoshioka, H., Nakahira, Y., and Shiina, T. (2012). Chloroplast-mediated activation of plant immune signalling in Arabidopsis. Nat Commun **3,** 926.

Oh, S.K., Young, C., Lee, M., Oliva, R., Bozkurt, T.O., Cano, L.M., Win, J., Bos, J.I., Liu, H.Y., van Damme, M., Morgan, W., Choi, D., Van der Vossen, E.A., Vleeshouwers, V.G., and Kamoun, S. (2009). In planta expression screens of Phytophthora infestans RXLR effectors reveal diverse phenotypes, including activation of the Solanum bulbocastanum disease resistance protein Rpi-blb2. Plant Cell **21,** 2928-2947.

Oikawa, K., Yamasato, A., Kong, S.G., Kasahara, M., Nakai, M., Takahashi, F., Ogura, Y., Kagawa, T., and Wada, M. (2008). Chloroplast outer envelope protein CHUP1 is essential for chloroplast anchorage to the plasma membrane and chloroplast movement. Plant Physiol **148,** 829-842.

Oikawa, K., Kasahara, M., Kiyosue, T., Kagawa, T., Suetsugu, N., Takahashi, F., Kanegae, T., Niwa, Y., Kadota, A., and Wada, M. (2003). Chloroplast unusual positioning1 is essential for proper chloroplast positioning. Plant Cell **15,** 2805-2815.

Pandey, P., Leary, A.Y., Tumtas, Y., Savage, Z., Dagvadorj, B., Duggan, C., Yuen, E.L., Sanguankiattichai, N., Tan, E., Khandare, V., Connerton, A.J., Yunusov, T., Madalinski, M., Mirkin, F.G., Schornack, S., Dagdas, Y., Kamoun, S., and Bozkurt, T.O. (2021). An oomycete effector subverts host vesicle trafficking to channel starvation-induced autophagy to the pathogen interface. Elife **10**.

Pepperkok, R., Squire, A., Geley, S., and Bastiaens, P.I. (1999). Simultaneous detection of multiple green fluorescent proteins in live cells by fluorescence lifetime imaging microscopy. Curr Biol **9,** 269-272.

Perez-Sancho, J., Tilsner, J., Samuels, A.L., Botella, M.A., Bayer, E.M., and Rosado, A. (2016). Stitching Organelles: Organization and Function of Specialized Membrane Contact Sites in Plants. Trends Cell Biol **26,** 705-717.

Petre, B., Lorrain, C., Saunders, D.G., Win, J., Sklenar, J., Duplessis, S., and Kamoun, S. (2016). Rust fungal effectors mimic host transit peptides to translocate into chloroplasts. Cell Microbiol **18,** 453-465.

Pettersen, E.F., Goddard, T.D., Huang, C.C., Meng, E.C., Couch, G.S., Croll, T.I., Morris, J.H., and Ferrin, T.E. (2021). UCSF ChimeraX: Structure visualization for researchers, educators, and developers. Protein Sci **30,** 70-82.

Prinz, W.A., Toulmay, A., and Balla, T. (2020). The functional universe of membrane contact sites. Nat Rev Mol Cell Biol **21,** 7-24.

Ratcliff, F., Martin-Hernandez, A.M., and Baulcombe, D.C. (2001). Technical Advance. Tobacco rattle virus as a vector for analysis of gene function by silencing. Plant J **25,** 237-245.

Rocchetti, A., Hawes, C., and Kriechbaumer, V. (2014). Fluorescent labelling of the actin cytoskeleton in plants using a cameloid antibody. Plant Methods **10,** 12.

Rodriguez-Herva, J.J., Gonzalez-Melendi, P., Cuartas-Lanza, R., Antunez-Lamas, M., Rio-Alvarez, I., Li, Z., Lopez-Torrejon, G., Diaz, I., Del Pozo, J.C., Chakravarthy, S., Collmer, A., Rodriguez-Palenzuela, P., and Lopez-Solanilla, E. (2012). A bacterial cysteine protease effector protein interferes with photosynthesis to suppress plant innate immune responses. Cell Microbiol **14,** 669-681.

Savage, Z., Duggan, C., Toufexi, A., Pandey, P., Liang, Y., Segretin, M.E., Yuen, L.H., Gaboriau, D.C.A., Leary, A.Y., Tumtas, Y., Khandare, V., Ward, A.D., Botchway, S.W., Bateman, B.C., Pan, I., Schattat, M., Sparkes, I., and Bozkurt, T.O. (2021). Chloroplasts alter their morphology and accumulate at the pathogen interface during infection by Phytophthora infestans. Plant J **107,** 1771-1787.

Schattat, M., Barton, K., Baudisch, B., Klosgen, R.B., and Mathur, J. (2011). Plastid stromule branching coincides with contiguous endoplasmic reticulum dynamics. Plant Physiol **155,** 1667-1677.

Schindelin, J., Arganda-Carreras, I., Frise, E., Kaynig, V., Longair, M., Pietzsch, T., Preibisch, S., Rueden, C., Saalfeld, S., Schmid, B., Tinevez, J.Y., White, D.J., Hartenstein, V., Eliceiri, K., Tomancak, P., and Cardona, A. (2012). Fiji: an open-source platform for biological-image analysis. Nat Methods **9,** 676-682.

Schiotz, O.H., Kaiser, C.J.O., Klumpe, S., Morado, D.R., Poege, M., Schneider, J., Beck, F., Klebl, D.P., Thompson, C., and Plitzko, J.M. (2024). Serial Lift-Out: sampling the molecular anatomy of whole organisms. Nat Methods **21,** 1684-1692.

Schmidt von Braun, S., and Schleiff, E. (2008). The chloroplast outer membrane protein CHUP1 interacts with actin and profilin. Planta **227,** 1151-1159.

Selvaraj, M., Toghani, A., Pai, H., Sugihara, Y., Kourelis, J., Yuen, E.L.H., Ibrahim, T., Zhao, H., Xie, R., Maqbool, A., De la Concepcion, J.C., Banfield, M.J., Derevnina, L., Petre, B., Lawson, D.M., Bozkurt, T.O., Wu, C.H., Kamoun, S., and Contreras, M.P. (2024). Activation of plant immunity through conversion of a helper NLR homodimer into a resistosome. PLoS Biol **22,** e3002868.

Shen, Z., Liu, Y.C., Bibeau, J.P., Lemoi, K.P., Tuzel, E., and Vidali, L. (2015). The kinesin-like proteins, KAC1/2, regulate actin dynamics underlying chloroplast light-avoidance in Physcomitrella patens. J Integr Plant Biol **57,** 106-119.

Shepherd, S., Yuen, E.L.H., Carella, P., and Bozkurt, T.O. (2023). The wheels of destruction: Plant NLR immune receptors are mobile and structurally dynamic disease resistance proteins. Curr Opin Plant Biol **74,** 102372.

Singh, R., Lee, S., Ortega, L., Ramu, V.S., Senthil-Kumar, M., Blancaflor, E.B., Rojas, C.M., and Mysore, K.S. (2018). Two Chloroplast-Localized Proteins: AtNHR2A and AtNHR2B, Contribute to Callose Deposition During Nonhost Disease Resistance in Arabidopsis. Mol Plant Microbe Interact **31,** 1280-1290.

Sohrt, K., and Soll, J. (2000). Toc64, a new component of the protein translocon of chloroplasts. J Cell Biol **148,** 1213-1221.

Stuttmann, J., Barthel, K., Martin, P., Ordon, J., Erickson, J.L., Herr, R., Ferik, F., Kretschmer, C., Berner, T., Keilwagen, J., Marillonnet, S., and Bonas, U. (2021). Highly efficient multiplex editing: one-shot generation of 8x Nicotiana benthamiana and 12x Arabidopsis mutants. Plant J **106,** 8-22.

Su, J., Yang, L., Zhu, Q., Wu, H., He, Y., Liu, Y., Xu, J., Jiang, D., and Zhang, S. (2018). Active photosynthetic inhibition mediated by MPK3/MPK6 is critical to effector-triggered immunity. PLoS Biol **16,** e2004122.

Suetsugu, N., Higa, T., Gotoh, E., and Wada, M. (2016). Light-Induced Movements of Chloroplasts and Nuclei Are Regulated in Both Cp-Actin-Filament-Dependent and -Independent Manners in Arabidopsis thaliana. PLoS One **11,** e0157429.

Suetsugu, N., Yamada, N., Kagawa, T., Yonekura, H., Uyeda, T.Q., Kadota, A., and Wada, M. (2010). Two kinesin-like proteins mediate actin-based chloroplast movement in Arabidopsis thaliana. Proc Natl Acad Sci U S A **107,** 8860-8865.

Suetsugu, N., Sato, Y., Tsuboi, H., Kasahara, M., Imaizumi, T., Kagawa, T., Hiwatashi, Y., Hasebe, M., and Wada, M. (2012). The KAC family of kinesin-like proteins is essential for the association of chloroplasts with the plasma membrane in land plants. Plant Cell Physiol **53,** 1854-1865.

Vormittag, S., Ende, R.J., Derre, I., and Hilbi, H. (2023). Pathogen vacuole membrane contact sites - close encounters of the fifth kind. Microlife **4,** uqad018.

Wada, M., and Kong, S.G. (2018). Actin-mediated movement of chloroplasts. J Cell Sci **131**.

Wang, S., Welsh, L., Thorpe, P., Whisson, S.C., Boevink, P.C., and Birch, P.R.J. (2018). The Phytophthora infestans Haustorium Is a Site for Secretion of Diverse Classes of Infection-Associated Proteins. mBio **9**.

Wellburn, A.R. (1994). The Spectral Determination of Chlorophylls a and b, as well as Total Carotenoids, Using Various Solvents with Spectrophotometers of Different Resolution. Journal of Plant Physiology **144,** 307-313.

Whisson, S.C., Boevink, P.C., Moleleki, L., Avrova, A.O., Morales, J.G., Gilroy, E.M., Armstrong, M.R., Grouffaud, S., van West, P., Chapman, S., Hein, I., Toth, I.K., Pritchard, L., and Birch, P.R. (2007). A translocation signal for delivery of oomycete effector proteins into host plant cells. Nature **450,** 115-118.

Wu, C.H., Abd-El-Haliem, A., Bozkurt, T.O., Belhaj, K., Terauchi, R., Vossen, J.H., and Kamoun, S. (2017). NLR network mediates immunity to diverse plant pathogens. Proc Natl Acad Sci U S A **114,** 8113-8118.

Xu, Q., Tang, C., Wang, X., Sun, S., Zhao, J., Kang, Z., and Wang, X. (2019). An effector protein of the wheat stripe rust fungus targets chloroplasts and suppresses chloroplast function. Nat Commun **10,** 5571.

Yan, P., Shen, W., Gao, X., Li, X., Zhou, P., and Duan, J. (2012). High-throughput construction of intron-containing hairpin RNA vectors for RNAi in plants. PLoS One **7,** e38186.

Yang, K.Y., Liu, Y., and Zhang, S. (2001). Activation of a mitogen-activated protein kinase pathway is involved in disease resistance in tobacco. Proc Natl Acad Sci U S A **98,** 741-746.

Yuen, E.L.H., Shepherd, S., and Bozkurt, T.O. (2023). Traffic Control: Subversion of Plant Membrane Trafficking by Pathogens. Annu Rev Phytopathol **61,** 325-350.

Yuen, E.L.H., Leary, A.Y., Clavel, M., Tumtas, Y., Mohseni, A., Zhao, J., Picchianti, L., Jamshidiha, M., Pandey, P., Duggan, C., Cota, E., Dagdas, Y., and Bozkurt, T.O. (2024a). A RabGAP negatively regulates plant autophagy and immune trafficking. Curr Biol **34,** 2049-2065 e2046.

Yuen, E.L.H., Tumtas, Y., King, F., Ibrahim, T., Chan, L.I., Evangelisti, E., Tulin, F., Sklenar, J., Menke, F.L.H., Kamoun, S., Bubeck, D., Schornack, S., and Bozkurt, T.O. (2024b). A pathogen effector co-opts a host RabGAP protein to remodel pathogen interface and subvert defense-related secretion. Sci Adv **10,** eado9516.
