# Supplementary material for: Membrane contact sites between chloroplasts and the pathogen interface underpin plant focal immune responses
Source: Plant Cell. 2025 Sep 5;37(9):koaf214. doi: 10.1093/plcell/koaf214 (PMC12481158; doi:10.1093/plcell/koaf214)
Supplement: koaf214_Supplementary_Data [file koaf214_supplementary_data.zip › TPC-2025-0380R1_Supplementary Video Legends.pdf]

Supplementary Video Legends

Supplementary Movie S1. 3D projection of chup1 KO plants in Figure S1K. Chloroplasts are shown in cyan. The movie was generated from confocal z-stacks of the entire epidermal layer using the “3D Project” function in Fiji, with the “Brightest Point” projection method. Rotation was performed around the Y-axis with a 1° angle increment.

Supplementary Movie S2. 3D projection of FNR plants in Figure S1K. Chloroplasts are shown in cyan. The movie was generated from confocal z-stacks of the entire epidermal layer using the “3D Project” function in Fiji, with the “Brightest Point” projection method. Rotation was performed around the Y-axis with a 1° angle increment.
