# Supplementary material for: Membrane contact sites between chloroplasts and the pathogen interface underpin plant focal immune responses
Source: Plant Cell. 2025 Sep 5;37(9):koaf214. doi: 10.1093/plcell/koaf214 (PMC12481158; doi:10.1093/plcell/koaf214)
Supplement: koaf214_Supplementary_Data [file koaf214_supplementary_data.zip › Supplement References.docx]

**References**

**Breuers, F.K., Brautigam, A., Geimer, S., Welzel, U.Y., Stefano, G., Renna, L., Brandizzi, F., and Weber, A.P.** (2012). Dynamic Remodeling of the Plastid Envelope Membranes - A Tool for Chloroplast Envelope in vivo Localizations. Front Plant Sci **3,** 7.

**Chaparro-Garcia, A., Schwizer, S., Sklenar, J., Yoshida, K., Petre, B., Bos, J.I., Schornack, S., Jones, A.M., Bozkurt, T.O., and Kamoun, S.** (2015). Phytophthora infestans RXLR-WY Effector AVR3a Associates with Dynamin-Related Protein 2 Required for Endocytosis of the Plant Pattern Recognition Receptor FLS2. PLoS One **10,** e0137071.

**Contreras, M.P., Pai, H., Tumtas, Y., Duggan, C., Yuen, E.L.H., Cruces, A.V., Kourelis, J., Ahn, H.K., Lee, K.T., Wu, C.H., Bozkurt, T.O., Derevnina, L., and Kamoun, S.** (2023b). Sensor NLR immune proteins activate oligomerization of their NRC helpers in response to plant pathogens. EMBO J **42,** e111519.

**Dagdas, Y.F., Pandey, P., Tumtas, Y., Sanguankiattichai, N., Belhaj, K., Duggan, C., Leary, A.Y., Segretin, M.E., Contreras, M.P., Savage, Z., Khandare, V.S., Kamoun, S., and Bozkurt, T.O.** (2018). Host autophagy machinery is diverted to the pathogen interface to mediate focal defense responses against the Irish potato famine pathogen. Elife **7**.

**Pandey, P., Leary, A.Y., Tumtas, Y., Savage, Z., Dagvadorj, B., Duggan, C., Yuen, E.L., Sanguankiattichai, N., Tan, E., Khandare, V., Connerton, A.J., Yunusov, T., Madalinski, M., Mirkin, F.G., Schornack, S., Dagdas, Y., Kamoun, S., and Bozkurt, T.O.** (2021). An oomycete effector subverts host vesicle trafficking to channel starvation-induced autophagy to the pathogen interface. Elife **10**.

**Ratcliff, F., Martin-Hernandez, A.M., and Baulcombe, D.C.** (2001). Technical Advance. Tobacco rattle virus as a vector for analysis of gene function by silencing. Plant J **25,** 237-245.

**Rocchetti, A., Hawes, C., and Kriechbaumer, V.** (2014). Fluorescent labelling of the actin cytoskeleton in plants using a cameloid antibody. Plant Methods **10,** 12.
