# Supplementary material for: Membrane contact sites between chloroplasts and the pathogen interface underpin plant focal immune responses
Source: Plant Cell. 2025 Sep 5;37(9):koaf214. doi: 10.1093/plcell/koaf214 (PMC12481158; doi:10.1093/plcell/koaf214)
Supplement: koaf214_Supplementary_Data [file TPC-2025-0380 PEER REVIEW REPORT.pdf › TPC-2025-0380 PEER REVIEW REPORT.pdf]

Email

**Manuscript #**	TPC2024-RA-11158
**Title**	Membrane Contact Sites Between Chloroplasts and Pathogen Interface Underpin Plant Focal Immune Responses
**Corresponding Author**	Dr. Tolga Osman Bozkurt (Imperial College London)
**Date:**	09-Sep-2024 10:16:53
**Last Sent:**	09-Sep-2024 10:16:53
**Created By:**	Wenbo Ma
**From:**	bcmeyers@ucdavis.edu
**To:**	o.bozkurt@imperial.ac.uk
**CC:**	wenbo.ma@tsl.ac.uk
**BCC:**	wenbo.ma@tsl.ac.uk
**Subject:**	TPC2024-RA-11158 Decision Letter
**Email**	Dr. Tolga Osman Bozkurt<br>Imperial College London<br>London SW7 2AZ<br>United Kingdom<br><br><br>Dear Dr. Bozkurt (Dear Tolga):<br><br>We have received reviews of your manuscript entitled "Membrane Contact Sites Between Chloroplasts and Pathogen Interface Underpin Plant Focal Immune Responses." Thank you for submitting your best work to The Plant Cell. The editorial board agrees that the work you describe is substantive and falls within the scope of the journal. Based on the reviewers' comments, this work may become acceptable for publication, pending revision and re-review.<br><br>As you will see from the detailed external reviewer comments below, although they all agree that this work represents novel biology and the observations are intriguing, each reviewer also pointed out weaknesses that need to be strengthened. We ask you to pay attention to the following points, which are shared by more than one reviewers:<br><br>1. Higher resolution imagining, such as TEM or 3D electron tomography, should be used to clearly define the MCS between the chloroplast membrane and the PM or EHM, and the localization of CHUP1 and KAC1 at these MCS.<br>2. protein-protein interaction of CHUP1-KAC1 should be substantiated. The interaction interface should be characterised and mutagenesis should be used to 1) confirm the interaction; 2) study the consequence of the interaction, such as contribution of chloroplasts-EHM MCS and focal immunity.<br><br>I should stress that we are reluctant to see manuscripts undergoing multiple rounds of revision and would be unlikely to offer you more than one chance to satisfy the reviewers. Therefore, it is essential to fully address the reviewers' concerns. Please contact us if there are ambiguous comments or if you wish to discuss the revision.<br><br>Given the nature of the comments, we are offering you 60 days from when we have issued

this decision to complete the revision. If a revision is not returned within this time frame, and if you have not been granted an extension, we will withdraw the manuscript, which will leave you free to submit the work elsewhere. If you need an extension, we encourage you to contact us at any point before the 60 days have passed.

When you are ready to submit the revised version, please upload a highlighted copy that indicates all changes made in response to the editor and reviewer recommendations. Include an itemized list of all changes made in response to each of the reviewer's suggestions in the "Response to Reviewers" section; please note that reviewers do not have access to your cover letter, nor was this decision letter shared with them.

Thank you for the privilege of reviewing your work. We look forward to receiving your revised manuscript.

On behalf of the editorial board,

Wenbo Ma, Board of Reviewing Editors
Wenbo Ma, Senior Editor
Blake Meyers, Editor-in-Chief

The Plant Cell

<B>Please note the following:</>

-The Plant Cell now requires authors to complete and submit an author revisions checklist upon submission of a revised manuscript. The aim of the checklist is to aid authors in preparing a high-quality manuscript, facilitate the review and assessment of revised manuscripts, and help to ensure that journal standards are maintained across the board. If your manuscript is accepted, the completed checklist will be published as supplemental material attached to the article online. Please download a copy of the checklist (pdf fillable form) at this link, for submission with your revised manuscript: https://tpc.msubmit.net/html/Author_Revisions_Checklist.pdf.

-Supplemental materials should be restricted to large datasets and tables, presentation of replicates, and validation of reagents, methods, or genotypes. Any data that are used to support the major claims must be in the main manuscript. Supplemental figure legends must indicate what figure in the main manuscript is supported by the supplemental data presented. Please justify how each of the supplemental figures meet the criteria.

-Sampling methods and nature of "biological replicates" should be described precisely (i.e. different plants, parts of plants, pooled tissue, independent pools of tissue, sampled at different times, etc), along with a clear description of and rationale for any statistical analyses conducted. The reader should know exactly what was sampled; what forms the basis of the calculation of any means and statistical parameters reported. This is also necessary to ensure that proper statistical analysis was conducted.

-Want to add this revision deadline to your calendar? Click below!
[ical: The Plant Cell TPC2024-RA-11158 Revision Due Date Summary|The Plant Cell TPC2024-RA-11158 Revision Due Date Description|08-Nov-2024]

--- Reviewer comments:

Reviewer #1 (Comments for the Author):

Yuen and co-workers investigate the role of membrane contact sites (MCS) in plant immunity, specifically those established between chloroplasts and the extrahaustorial membrane (EHM) that forms around the haustoria of the pathogen Phytophthora infestans. The authors identify the molecular elements required for the establishment of such structures and their role in the immune response. The formation of these foci between organelles (chloroplasts and EHM) results in a positive regulation of plant defense mediated by the deposition of callose. This work represents a clear advancement in understanding the importance of interorganelle MCS during plant-pathogen interactions and opens up new avenues for future research. The presented work is well-executed, the experiments are well-designed, and the rationale and conclusions-adequately supported by the results-are presented clearly and concisely.

Next, I would like to highlight a few points that could be considered by the authors for the improvement of the manuscript.

Major Revisions:

In the introduction, there may be a lack of citations related to the manipulation of chloroplasts by effectors from various pathogens-fungi, bacteria, and viruses. While it is true that there has been a considerable increase in publications in this specific area in recent years, this does not diminish the importance of citing the most current and notable references.

The microscopy presented by the authors shows a clear co-localization of CHUP1 and KAC1, as well as the positive role of both elements in the immune response. However, in my humble opinion and based on the general standards, the confirmation of the interaction between both proteins supported by only one method (co-IP) does not seem sufficient to me. Could the authors confirm the PPI using another method (e.g., Y2H, pull-down, ...)?

In line with this suggestion, and in order to delve deeper into the intimate relationship between both proteins, I propose that the authors could provide more detailed information on the CHUP1-KAC1 PPI interface; for example, by using truncated versions of both proteins (Yuen and co-workers only deleted the actin-binding domain present in CHUP1 - CHUP1ΔABD), followed by confocal-based subcellular localization confirmation along with co-IP and Y2H? Alphafold-based predictions too?

Although figure S5 contains supplementary information to figure 5, figure S5 itself does not provide results that support the title regarding the interaction between KAC1 and CHUP1. Therefore, it would be appropriate if the title of figure S5 were worded differently.

Minor Revisions:

Do NHRA/B localize to MCS? Do they co-localize in foci with CHUP1 and KAC1 around the EHM?

Reviewer #2 (Comments for the Author):

In this study, the authors observed that in tobacco leaves treated with Phytophthora infestans, the chloroplast outer membrane protein CHUP1 formed punctate structures at the interface between the chloroplast membrane and either the PM or the EHM. The loss of

CHUP1 led to impaired callose deposition at the pathogen penetration site, thereby compromising host resistance to fungal infection. The authors also identified that KAC1, a protein localized to the PM and cytosol, plays a similar role to CHUP1 in plant focal immune responses. Upon fungal infection, KAC1 exhibited punctate structures that colocalized with CHUP1. Since KAC1 and CHUP1 interact, the authors concluded that these two proteins form membrane contact sites (MCS) between chloroplasts and the PM or EHM surrounding the pathogen haustorium.

This study is intriguing, as identifying new types of MCS and their underlying molecular components is crucial for understanding the biological significance of intraorganellar communication under biotic stress. However, the evidence provided in the current work does not fully substantiate these ambitious claims. My primary concerns are outlined below:

The existence of MCS between the chloroplast membrane and the PM or EHM needs to be validated using super-resolution imaging techniques such as transmission electron microscopy (TEM) or 3D electron tomography, if possible. Since MCS are typically around 30 nm in diameter without direct membrane fusion, this is already below the resolution limit of confocal microscopy. Furthermore, I recommend using immuno-electron microscopy to confirm the localization of CHUP1 and KAC1 at these MCS.

In Figures 2 and 5, it is unclear where in the cells these images were taken. Were panels A and B acquired from different cellular locations compared to panels C and D? Please stated in the method. If so, why do chloroplasts also form MCS with the rest of the PM in addition to the EHM? Only one chloroplast is shown in these images; how frequently do the authors detect these membrane contacts in cells? Please provide lower magnification fluorescence images to offer an overview of the spatial relationships between chloroplasts, the PM, and the EHM around the pathogen penetration site. Additionally, two-color images showing CHUP1-GFP and RFP-REM1.3 before pathogen infection should be included.

It would be helpful to see time-lapse images demonstrating the process of MCS formation among CHUP1, the PM, and the EHM during pathogen infection. Are the MCS between the chloroplast and the PM or EHM stable or transient?

In lines 268-271, the conclusions drawn are not adequately supported by the data. In Figures S1G-H and I-K, chloroplast accumulation around the EHM in chup1 mutants showed no significant differences from the WT, suggesting that MCS formed by CHUP1 are dispensable for this process. Are you certain that these MCS, or even chloroplasts surrounding the EHM, are physiologically relevant to the focal development of callose at the haustorium interface?

Additional genetic evidence is required to establish the biological importance of the CHUP1/KAC1 complex in the context of MCS and focal immune responses. If possible, identifying and mutating key interacting domains or amino acids in CHUP1/KAC1 would provide strong supporting evidence.

It is well-established that cortical actin is required for the focal response to fungal infection. How does the CHUP1/KAC1-mediated MCS form upon pathogen infection? Is cortical actin or cp-actin involved in this process, and if so, how? Moreover, what are the upstream signaling molecules that directly lead to the accumulation of CHUP1 at the chloroplast-PM or chloroplast-EHM interface?

Reviewer #3 (Comments for the Author):

The manuscript by Yuen et al. reports defence-related membrane contact sites comprising a membrane tethering complex between chloroplasts and EHM. The association between these two kinds of membranes may depend on the interaction between the chloroplast outer membrane protein CHUP1 and plasma membrane protein KAC1. The loss of function of CHUP1 or KAC1 reduces the focal deposition of callose, thereby affecting plant defence against pathogens. The concept is exciting, but the mechanism needs further study, especially the MCS and immune response results. The current data demonstrate that the interaction between CHUP1 and KAC1 is required for plant immunity, but it is speculative to interpret this phenotype as MCS-related. Further experimentation is needed to show the direct contribution of chloroplasts-EHM MCS to immune responses.

Major issues:

1. The authors suggested that MCS between chloroplast and EHM plays an essential role in plant immune response. However, it is unclear whether CHUP or KAC's loss of function affects MCS's formation. It is essential that the authors provide further data to clarify this. What happens to the Ch-EHM MCS if the interaction between CHUP1 and KAC1 is inhibited? Providing more direct evidence on MCS formation and plant immunity is necessary.
2. In addition, is the immunity phenotype in the chup1 mutant related to EHM-chloroplast MCS formation? We know chloroplasts are implicated in the immune response. Are we expecting an immunity-related phenotype when chloroplast functions are affected in general? Does the chup1 mutant exhibit a general defect in chloroplast function? In other words, the causal relationship between the chup1 phenotype and MCS establishment is unclear. It is not convincing to say "chloroplast-EHM MCS positively regulates plant focal immunity."
3. The authors show that the loss of function of CHUP or KAC affects the accumulation of callose around the haustoria, but the underlying mechanism is unclear. Further data are necessary to show how these two proteins are responsible for this phenotype.
4. Most subcellular structures are recruited to the EHM region after infection in tobacco leaf epidermal cells. So, is the CHUP1-KAC1 regulated pathway specific to chloroplast-EHM MCS? Could it affect the enrichment of other membrane or cytoskeletal structures at the EHM?
5. Since this is likely the first time Ch-EHM MCS has been discovered, I think the authors should better define this type of MCS. Some additional TEM studies will be helpful, as the resolution of light microscopy is insufficient to resolve MCS.
6. Figure 4: It is necessary to use more than one RNAi line to study the phenotype.
7. Figure 5: Please use multiple methods to confirm the interaction between CHUP and KAC.

Minor issues:

1. 1. Figure S1K shows the chloroplast distribution in FNR and chup1 mutants. It seems that these two images show different layers of the cell. Please also label the cell margin.
2. Figure S4, the expression level of housekeeping gene GAPDH is different on the left panel. Please ensure the amount of the cDNA template used for each reaction is identical.

Reviewing Editor comments:

Click here to access/download;Response to Reviewer;Response to Editors and Reviewers with Performed

IMPERIAL

31st March 2025

Dear Prof. Ma and Prof. Meyers,

We sincerely appreciate the time and effort the Plant Cell editorial board and the three reviewers dedicated to meticulously reviewing our manuscript entitled **"Membrane Contact Sites Between Chloroplasts and Pathogen Interface Underpin Plant Focal Immune Responses" (Paper #: TPC2024-RA-11158).** The thoughtful and constructive feedback we received was invaluable in enhancing the overall quality and clarity of our research. In response to the insights and concerns raised, we have diligently worked to refine our manuscript, incorporating additional experimental data where deemed necessary to fortify our findings. **For ease of review, we have indicated all revisions in the combined manuscript file using red text.**

Enclosed below is a detailed, **point-by-point response to the editors' and reviewers' comments**, outlining the specific actions we have taken to address their concerns.

Sincerely,

Dr. Tolga Bozkurt

Editors' Comments

1. Higher resolution imaging, such as TEM or 3D electron tomography, should be used to clearly define the MCS between the chloroplast membrane and the PM or EHM, and the localization of CHUP1 and KAC1 at these MCS.

In response to suggestions, we have employed both cryogenic electron tomography (CryoET) and transmission electron microscopy (TEM) with immunogold labeling to investigate membrane contact sites (MCS) between the chloroplast and the plasma membrane (PM) and to assess the localization of CHUP1.

CryoET: Data from cryoET revealed clear contact points between the chloroplast outer membrane and plasma membrane. These take the form of membrane protrusions linked by a layer of ~30 nm long and potentially highly flexible protein. The thickness of this protein layer is consistent with the predicted size of the putative CHUP1:KAC1 complex. The high quality of the data is apparent, as we have been able to resolve RuBisCo complexes in the chloroplast stroma and even individual cellulose fibres in the cell wall.

Importantly, to obtain these data we had to develop a new and highly technically challenging workflow for cryogenic imaging of plant tissues (protocol described in Methods). This would be the first published example of a similar approach used to study plant cells.

Immunogold TEM labeling: We performed immunogold labeling using anti-GFP antibodies to localize CHUP1:GFP. However, the results were inconclusive due to non-specific labeling of both CHUP1:GFP and EV:GFP. Although we observed instances where gold particles labeled CHUP1:GFP at the chloroplast-PM MCS, these findings are not definitively conclusive, as we also observed widespread non-specific immunogold labeling across the chloroplast in both experimental and control samples (see figure below). This is consistent with the known tendency of immunogold labelling for non-specific binding, especially with specimens containing plastids. Given these limitations, we conclude that TEM-based immunogold labeling is not a reliable method for confirming CHUP1 localization at MCS in our system. Reassuringly, in the immunogold TEM data (which was prepared through high pressure freezing and freeze-substitution), we have noticed membrane protrusions with those observed in cryoET.

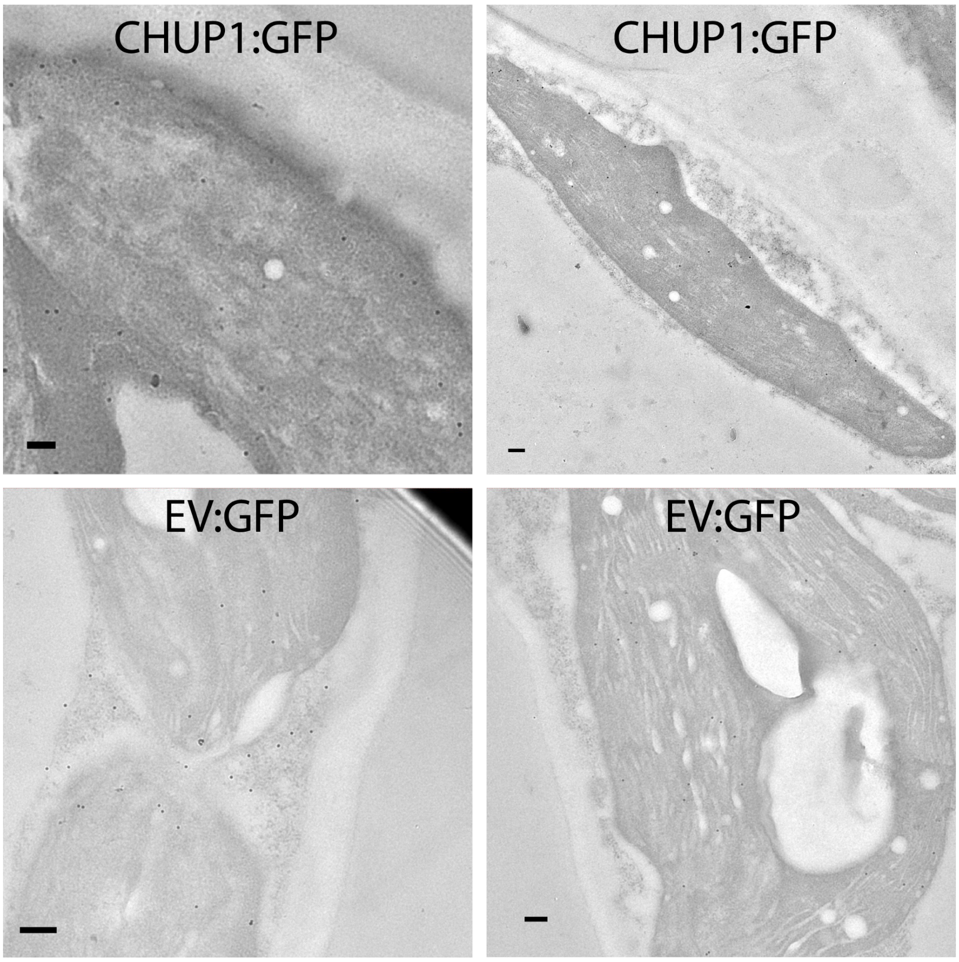

This figure shows immunogold labelling of CHUP1:GFP and control EV:GFP samples. Scale bar represents 200 nm. Specimens have been prepared by high pressure freezing and freeze-substitution.

Together, these complementary high-resolution imaging approaches provide strong support for the presence of MCS between the chloroplast and the PM. However, due to the limitations of immunogold TEM, we cannot conclusively determine the localization of CHUP1 at these sites. Future studies will be needed to investigate the precise localization and the structure of CHUP1 at chloroplast-PM contact sites.

2. The protein-protein interaction of CHUP1-KAC1 should be substantiated. The interaction interface should be characterised and mutagenesis should be used to a) confirm the interaction; b) study the consequence of the interaction, such as contribution of chloroplasts-EHM MCS and focal immunity.

We appreciate the suggestions regarding further characterization of the CHUP1-KAC1 interaction interface. While we agree that such detailed mechanistic studies would be valuable, we believe they represent a significant undertaking beyond the scope of the current manuscript:

Firstly, our computational analysis using AlphaFold3 predicts a complex, multidimensional interaction interface between CHUP1 and KAC1 (Figure A below). This complexity suggests that a comprehensive mutagenesis approach would require a systematic analysis of multiple potential interaction domains. To explore this further, we conducted multiple independent preliminary truncation experiments. Consistent with the AlphaFold3 predictions, even with extensive truncations of CHUP1 on both n-terminal side and c-terminal side, the two proteins still exhibited robust interaction *in planta* (Figure B below). These results confirm and indicate that CHUP1 and KAC1 interact extensively.

Secondly, this manuscript presents the novel discovery that CHUP1 and KAC1 play crucial roles in chloroplast-mediated immunity. Our co-immunoprecipitation data, subcellular localization studies, and genetic analyses provide strong evidence for their functional interaction in this context. It is worth noting that the functions of CHUP1 and KAC1 in anchoring chloroplasts to the plasma membrane and mediating chloroplast photorelocation have been well-established in previous genetic studies (Suetsugu et al., 2010; Oikawa et al., 2008). Our work builds upon this, establishing protein-protein interaction between CHUP1 and KAC1, as well as showing their punctate colocalization at the chloroplast-PM interface and demonstrating their novel role in pathogen defense contexts, specifically at the extrahaustorial membrane interface. Importantly, while mapping the precise interaction interface would provide additional mechanistic insights, it would not alter the conclusions and discoveries of our study.

Lastly, the detailed molecular characterization suggested here represents a natural progression of this work but would require extensive additional experiments, including: systematic domain mapping through truncation and point mutations; functional validation of these mutants *in planta*; assessment of their impact on chloroplast-EHM MCS formation; and evaluation of their contribution to focal immunity. Such comprehensive analyses would conservatively require several years of dedicated work by a postdoctoral researcher or PhD student, with associated funding requirements. We propose that our current findings establish the foundation for these future studies, which we are eager to pursue in subsequent work. The present manuscript focuses on the discovery and initial characterization of this novel immune-related roles of CHUP1 and KAC1, which we believe represents a significant advance in understanding organelle-mediated immunity, even without the detailed interaction interface mapping.

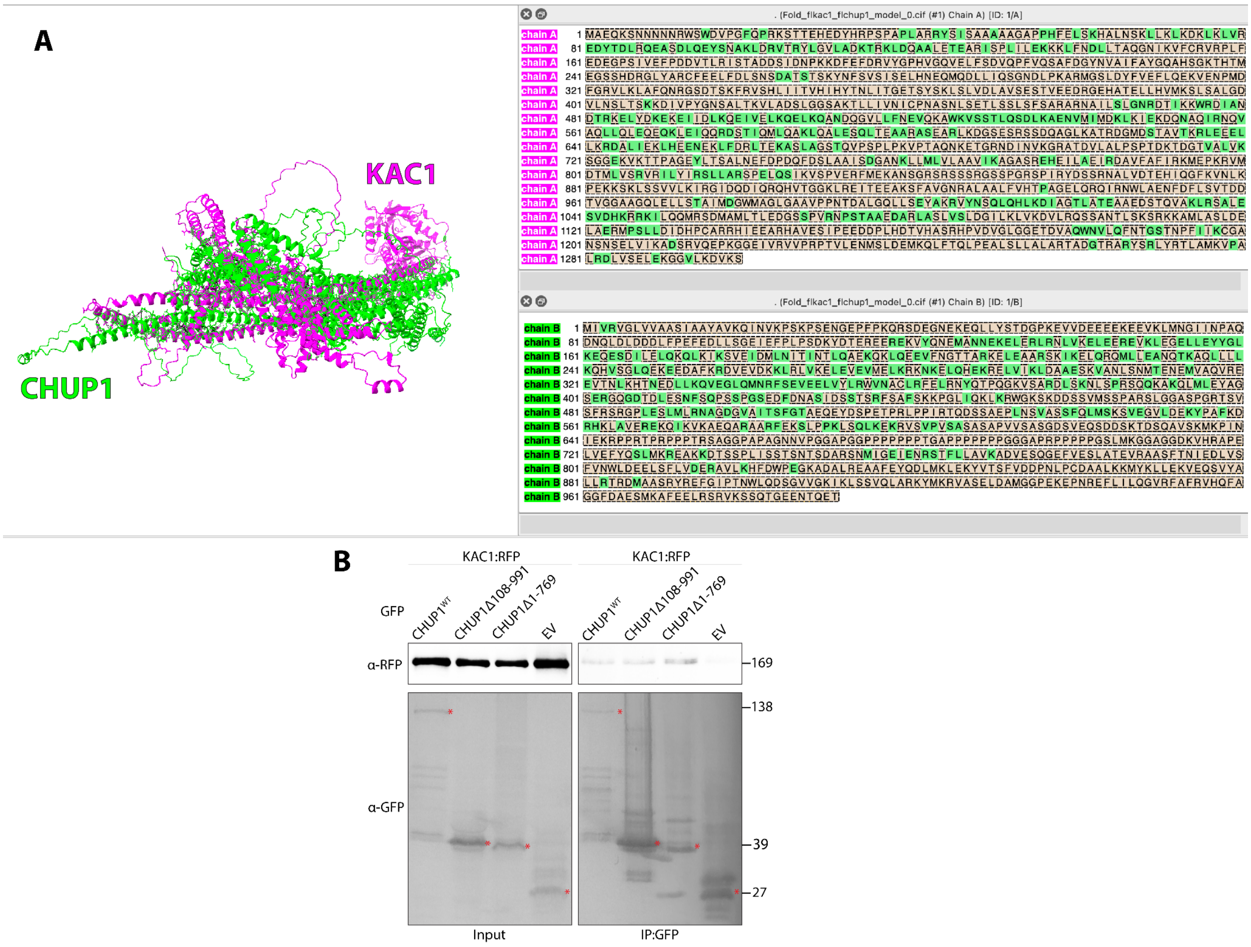

(A) AlphaFold3 predicted model of CHUP1 and KAC1 interaction. CHUP1 is depicted in lime, and KAC1 is depicted in magenta. The putative interacting residues between CHUP1 and KAC1 are predicted by ChimeraX with a stringent distance of 4 Å.

(B) KAC1 interacts with full length and the truncates CHUP1, but not with EV control. KAC1:RFP was transiently co-expressed with CHUP1(WT):GFP, CHUP1Δ108-991:GFP (truncated c-terminal), CHUP1Δ1-769:GFP (truncated n-terminal), or EV:GFP. IPs were obtained with anti-GFP antibody. Total protein extracts were immunoblotted. Red asterisks indicate expected band sizes. Numbers on the right indicate kDa values.

Reviewer #1 (Comments for the Author):

Yuen and co-workers investigate the role of membrane contact sites (MCS) in plant immunity, specifically those established between chloroplasts and the extrahaustorial membrane (EHM) that forms around the haustoria of the pathogen Phytophthora infestans. The authors identify the molecular elements required for the establishment of such structures and their role in the immune response. The formation of these foci between organelles (chloroplasts and EHM) results in a positive regulation of plant defense mediated by the deposition of callose. This work represents a clear advancement in understanding the importance of interorganelle MCS during plant-pathogen interactions and opens up new avenues for future research. The presented work is well-executed, the experiments are well-designed, and the rationale and conclusions-adequately supported by the results-are presented clearly and concisely.

Next, I would like to highlight a few points that could be considered by the authors for the improvement of the manuscript.

Major Revisions:

1. In the introduction, there may be a lack of citations related to the manipulation of chloroplasts by effectors from various pathogens-fungi, bacteria, and viruses. While it is true that there has been a considerable increase in publications in this specific area in recent years, this does not diminish the importance of citing the most current and notable references.

Action: We thank the reviewer for noticing this. We have incorporated and cited examples that address the manipulation of chloroplasts by effectors. We have added the following paragraph into the manuscript introduction with citations:

"For instance, effectors from a diverse range of pathogens target chloroplasts to interfere with immune signaling, photosynthetic activity, stromule formation, and salicylic acid biosynthesis, reinforcing the emerging role of chloroplasts in plant immunity (Jelenska et al., 2007; Jelenska et al., 2010; Rodriguez-Herva et al., 2012; Gao et al., 2020; Medina-Puche et al., 2020; Savage et al., 2021)."

2. The microscopy presented by the authors shows a clear co-localization of CHUP1 and KAC1, as well as the positive role of both elements in the immune response. However, in my humble opinion and based on the general standards, the confirmation of the interaction between both proteins supported by only one method (co-IP) does not seem sufficient to me. Could the authors confirm the PPI using another method (e.g., Y2H, pull-down, ...)?

Response: Co-IP has been the gold standard for detecting protein-protein interactions under native conditions, and we have carefully optimized its use in our assays. Given that CHUP1 and KAC1 interact at the membrane interface, alternative approaches such as yeast two-hybrid (Y2H), which rely on nuclear localization, may not be suitable for confirming this interaction. Additionally, our confocal microscopy data showing the specific co-localization of both

proteins in punctate structures complement the co-IP results, providing further support for the CHUP1-KAC1 interaction. To ensure the robustness of our findings, we included multiple controls in our co-IP experiments. For example, Toc64, another chloroplast outer membrane protein, was used as a control, while the N-terminal region of CHUP1, which is required for its localization to the chloroplast outer envelope, served as a negative control.

Moreover, our findings align with previous studies suggesting that CHUP1 and KAC1 function together in cp-actin-mediated chloroplast movement and positioning (Suetsugu et al., 2012), further reinforcing the validity of this interaction. We have also successfully applied these methodologies in prior studies published in high-impact journals (Duggan et al., 2021; Pandey et al., 2018), which we believe provide a robust framework for validating protein-protein interactions. While we appreciate your suggestion, we believe that co-IP pull-down assays remain the most appropriate approach in this case.

3. In line with this suggestion, and in order to delve deeper into the intimate relationship between both proteins, I propose that the authors could provide more detailed information on the CHUP1-KAC1 PPI interface; for example, by using truncated versions of both proteins (Yuen and co-workers only deleted the actin-binding domain present in CHUP1 - CHUP1ΔABD), followed by confocal-based subcellular localization confirmation along with co-IP and Y2H? Alphafold-based predictions too?

Response: We appreciate the suggestions regarding further characterization of the CHUP1-KAC1 interaction interface. While we agree that such detailed mechanistic studies would be valuable, we believe they represent a significant undertaking beyond the scope of the current manuscript:

Firstly, our computational analysis using AlphaFold3 predicts a complex, multidimensional interaction interface between CHUP1 and KAC1 (Figure A below). This complexity suggests that a comprehensive mutagenesis approach would require a systematic analysis of multiple potential interaction domains. To explore this further, we conducted multiple independent preliminary truncation experiments. Consistent with the AlphaFold3 predictions, even with extensive truncations of CHUP1 on both n-terminal side and c-terminal side, the two proteins still exhibited robust interaction *in planta* (Figure B below). These results confirm and indicate that CHUP1 and KAC1 interact extensively.

Secondly, this manuscript presents the novel discovery that CHUP1 and KAC1 play crucial roles in chloroplast-mediated immunity. Our co-immunoprecipitation data, subcellular localization studies, and genetic analyses provide strong evidence for their functional interaction in this context. It is worth noting that the functions of CHUP1 and KAC1 in anchoring chloroplasts to the plasma membrane and mediating chloroplast photorelocation have been well-established in previous genetic studies (Suetsugu et al., 2010; Oikawa et al., 2008). Our work builds upon this, establishing protein-protein interaction between CHUP1 and KAC1, as well as showing their punctate colocalization at the chloroplast-PM interface and demonstrating their novel role in pathogen defense contexts, specifically at the extrahaustorial

membrane interface. Importantly, while mapping the precise interaction interface would provide additional mechanistic insights, it would not alter the conclusions and discoveries of our study.

Lastly, the detailed molecular characterization suggested here represents a natural progression of this work but would require extensive additional experiments, including: systematic domain mapping through truncation and point mutations; functional validation of these mutants *in planta*; assessment of their impact on chloroplast-EHM MCS formation; and evaluation of their contribution to focal immunity. Such comprehensive analyses would conservatively require several years of dedicated work by a postdoctoral researcher or PhD student, with associated funding requirements. We propose that our current findings establish the foundation for these future studies, which we are eager to pursue in subsequent work. The present manuscript focuses on the discovery and initial characterization of this novel immune-related roles of CHUP1 and KAC1, which we believe represents a significant advance in understanding organelle-mediated immunity, even without the detailed interaction interface mapping.

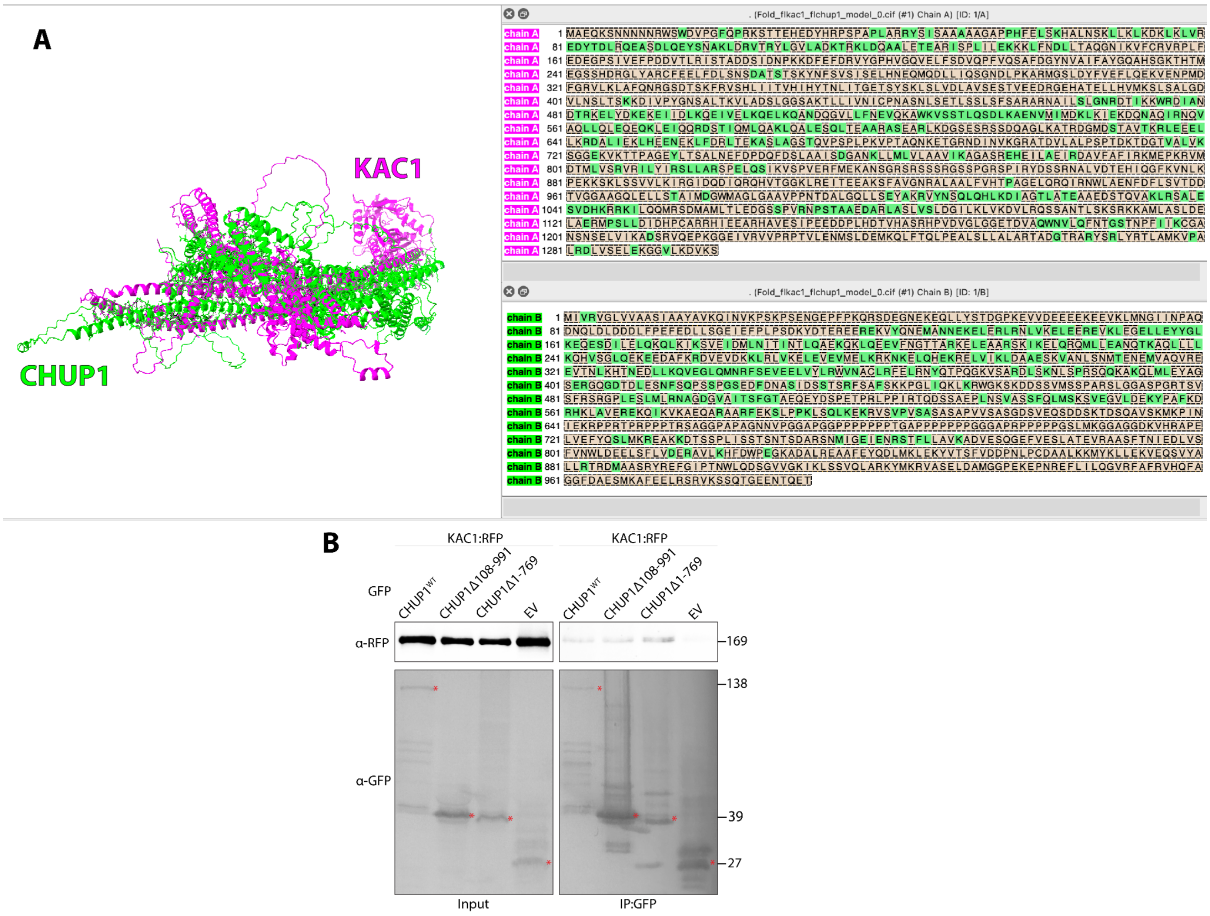

(A) AlphaFold3 predicted model of CHUP1 and KAC1 interaction. CHUP1 is depicted in lime, and KAC1 is depicted in magenta. The putative interacting residues between CHUP1 and KAC1 are predicted by ChimeraX with a stringent distance of 4 Å.

(B) KAC1 interacts with full length and the truncates CHUP1, but not with EV control. KAC1:RFP was transiently co-expressed with CHUP1(WT):GFP, CHUP1Δ108-991:GFP (truncated c-terminal), CHUP1Δ1-769:GFP (truncated n-terminal), or EV:GFP. IPs were

obtained with anti-GFP antibody. Total protein extracts were immunoblotted. Red asterisks indicate expected band sizes. Numbers on the right indicate kDa values.

4. Although figure S5 contains supplementary information to figure 5, figure S5 itself does not provide results that support the title regarding the interaction between KAC1 and CHUP1. Therefore, it would be appropriate if the title of figure S5 were worded differently.

Action: We thank the reviewer for pointing this out. The title of figure S5 has been changed to "KAC1 localizes to PM and requires CHUP1 to accumulate at MCS between chloroplasts and the PM."

Minor Revisions:

1. Do NHRA/B localize to MCS? Do they co-localize in foci with CHUP1 and KAC1 around the EHM?

Response: Thank you for this suggestion. While we have not investigated this aspect in the current study, it would be an intriguing direction for future research, which we may explore in a follow-up publication.

Reviewer #2 (Comments for the Author):

In this study, the authors observed that in tobacco leaves treated with Phytophthora infestans, the chloroplast outer membrane protein CHUP1 formed punctate structures at the interface between the chloroplast membrane and either the PM or the EHM. The loss of CHUP1 led to impaired callose deposition at the pathogen penetration site, thereby compromising host resistance to fungal infection. The authors also identified that KAC1, a protein localized to the PM and cytosol, plays a similar role to CHUP1 in plant focal immune responses. Upon fungal infection, KAC1 exhibited punctate structures that colocalized with CHUP1. Since KAC1 and CHUP1 interact, the authors concluded that these two proteins form membrane contact sites (MCS) between chloroplasts and the PM or EHM surrounding the pathogen haustorium.

This study is intriguing, as identifying new types of MCS and their underlying molecular components is crucial for understanding the biological significance of intraorganellar communication under biotic stress. However, the evidence provided in the current work does not fully substantiate these ambitious claims. My primary concerns are outlined below:

1. The existence of MCS between the chloroplast membrane and the PM or EHM needs to be validated using super-resolution imaging techniques such as transmission electron microscopy (TEM) or 3D electron tomography, if possible. Since MCS are typically around 30 nm in diameter without direct membrane fusion, this is already below the resolution limit of confocal microscopy. Furthermore, I recommend using immuno-electron microscopy to confirm the localization of CHUP1 and KAC1 at these MCS.

To address the suggestions, we have employed both cryogenic electron tomography (CryoET) and transmission electron microscopy (TEM) with immunogold labeling to investigate membrane contact sites (MCS) between the chloroplast and the plasma membrane (PM) and to assess the localization of CHUP1.

CryoET: Data from cryoET revealed clear contact points between the chloroplast outer membrane and plasma membrane. These take the form of membrane protrusions linked by a layer of ~30 nm long and potentially highly flexible protein. The thickness of this protein layer is consistent with the predicted size of the putative CHUP1:KAC1 complex. The high quality of the data is apparent, as we have been able to resolve RuBisCo complexes in the chloroplast stroma and even individual cellulose fibres in the cell wall.

Importantly, to obtain these data we had to develop a new and highly technically challenging workflow for cryogenic imaging of plant tissues (protocol described in Methods). This would be the first published example of a similar approach used to study plant cells.

Immunogold TEM labeling: We performed immunogold labeling using anti-GFP antibodies to localize CHUP1:GFP. However, the results were inconclusive due to non-specific labeling of both CHUP1:GFP and EV:GFP. Although we observed instances where gold particles labeled CHUP1:GFP at the chloroplast-PM MCS, these findings are not definitively

conclusive, as we also observed widespread non-specific immunogold labeling across the chloroplast in both experimental and control samples (see figure below). This is consistent with the known tendency of immunogold labelling for non-specific binding, especially with specimens containing plastids. Given these limitations, TEM-based immunogold labeling is not a reliable method for confirming CHUP1 localization at MCS in our system. Reassuringly, in the immunogold TEM data (which was prepared through high pressure freezing and freeze-substitution), we have notice membrane protrusions with those observed in cryoET.

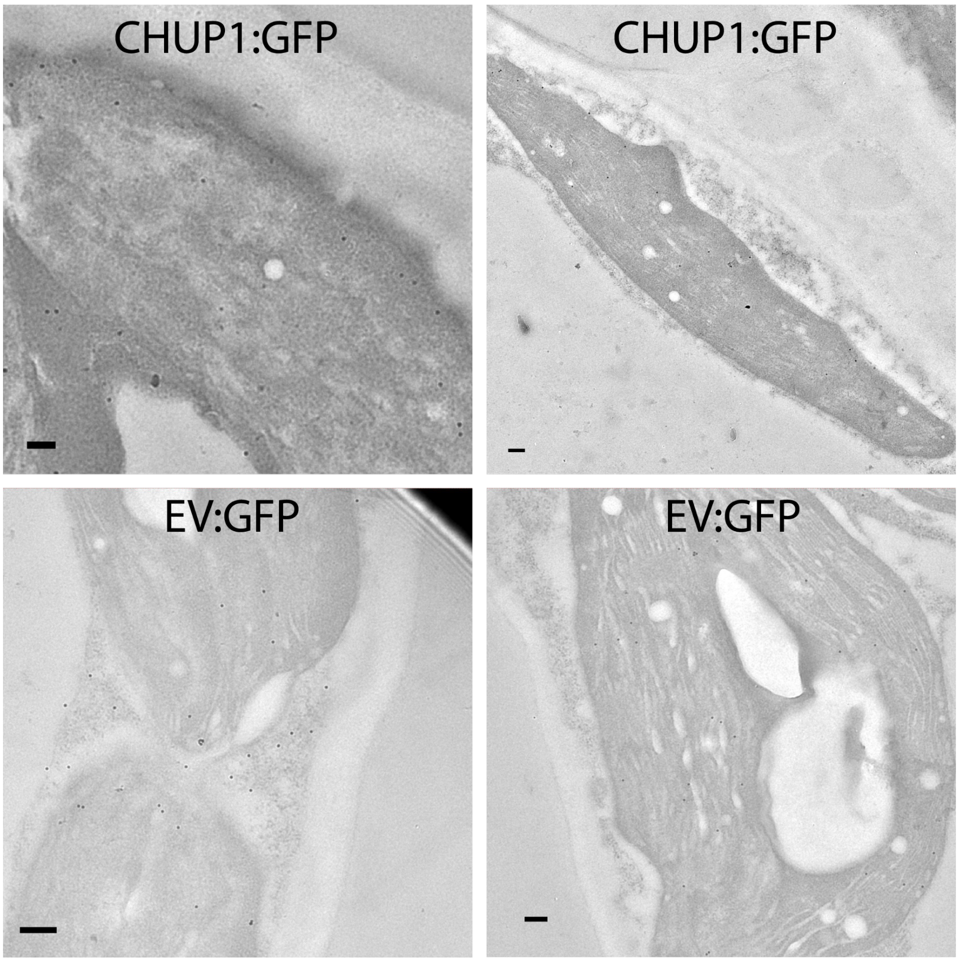

This figure shows immunogold labelling of CHUP1:GFP and control EV:GFP samples. Scale bar represents 200 nm. Specimens have been prepared by high pressure freezing and freeze-substitution.

Together, these complementary high-resolution imaging approaches provide strong support for the presence of MCS between the chloroplast and the PM. However, due to the limitations of immunogold TEM, we cannot conclusively determine the localization of CHUP1 at these sites. Future studies will be needed to investigate the precise localization and the structure of CHUP1 at chloroplast-PM contact sites.

2. In Figures 2 and 5, it is unclear where in the cells these images were taken. Were panels A and B acquired from different cellular locations compared to panels C and D? Please stated in the method. If so, why do chloroplasts also form MCS with the rest of the PM in addition to

the EHM? Only one chloroplast is shown in these images; how frequently do the authors detect these membrane contacts in cells? Please provide lower magnification fluorescence images to offer an overview of the spatial relationships between chloroplasts, the PM, and the EHM around the pathogen penetration site. Additionally, two-color images showing CHUP1-GFP and RFP-REM1.3 before pathogen infection should be included.

Action: In our original submission, Figure 2 panels A and B depict uninfected cells where RFP:REM1.3 labels the plasma membrane, while panels C and D show infected cells, with RFP:REM1.3 marking the EHM. We have mentioned this distinction in the figure legends. Also, Figure 2A already includes a two-color image of CHUP1-GFP and RFP:REM1.3 before pathogen infection. The representative images were selected with a higher magnification focus on individual chloroplasts to provide the most accurate depiction of MCS, as not all chloroplasts lie within the same focal plane.

P. infestans can form multiple haustorial penetration sites within a single infected cell. Therefore, we believe that the additional chloroplast–PM MCS observed in infected cells may serve as pre-positioned or mobile defense hubs, allowing chloroplasts to rapidly respond to multiple or future penetration attempts. Additionally, since *P. infestans* hyphae extend both within and along the exterior of infected cells, MCS-mediated chloroplast defenses, such as the localized production or release of reactive oxygen species (ROS), could also act against hyphal structures outside haustoria. However, in uninfected cells under normal conditions, chloroplasts are known to anchor to the PM to mediate light-dependent positioning. It is therefore reasonable that such anchoring also occurs outside the EHM. The CHUP1–KAC1 axis, originally identified for its role in chloroplast photorelocation (Suetsugu et al., 2010; Kong et al., 2024), appears to be co-opted for immune responses in our study. Thus, CHUP1-mediated chloroplast–PM contacts in uninfected cells may serve functions beyond immunity.

In response to the reviewer's request, we have revised Figure 2 extensively. We performed additional experiments with multiple biological replicates and have now included lower-magnification overview images to better illustrate the spatial relationships between chloroplasts, the PM, and the EHM near the pathogen penetration site. We have also quantified the percentage of chloroplasts exhibiting CHUP1 punctate accumulation at chloroplast-PM and chloroplast-EHM contact sites, which is now detailed in the updated legend.

To improve clarity, we replaced RFP:REM1.3 with NRC4:BFP as an EHM marker (Duggan et al., 2021), ensuring a clear distinction between PM and EHM labeling (Figure 2). Additionally, we have modified the figure legends to explicitly state when confocal images were taken from uninfected leaves with the sentence: "The leaves were not infected."

3. It would be helpful to see time-lapse images demonstrating the process of MCS formation among CHUP1, the PM, and the EHM during pathogen infection. Are the MCS between the chloroplast and the PM or EHM stable or transient?

Response: We thank the reviewer for this valuable suggestion. We agree that time-lapse imaging of chloroplast–PM/EHM MCS formation during *P. infestans* infection would provide

important insights into their dynamics. However, due to the technical challenges associated with live-cell imaging of *P. infestans* haustoria, particularly the need to visualize multiple components with sufficient resolution and stability over time, capturing these events remains difficult. These challenges are compounded by the hemibiotrophic lifestyle of *P. infestans*, which limits the temporal window for imaging. While these experiments are beyond the scope of the current study, we fully agree that they represent an important avenue for future research. Importantly, the conclusions of our manuscript are supported by multiple lines of static imaging, biochemical interaction data, and genetic evidence that collectively support the formation and functional relevance of these MCS during infection.

4. In lines 268-271, the conclusions drawn are not adequately supported by the data. In Figures S1G-H and I-K, chloroplast accumulation around the EHM in chup1 mutants showed no significant differences from the WT, suggesting that MCS formed by CHUP1 are dispensable for this process. Are you certain that these MCS, or even chloroplasts surrounding the EHM, are physiologically relevant to the focal development of callose at the haustorium interface?

Response: We thank the reviewer for highlighting this important point. In the manuscript, we stated that the enhanced susceptibility of *chup1* knockout plants is "most likely" due to impaired MCS formation, reflecting our caution in drawing definitive conclusions. We acknowledge that chloroplast positioning around the EHM is not significantly altered in the *chup1* mutant, as shown in Figures S1G–H and S1I–K. However, our key point is that CHUP1 is not required for chloroplast movement per se, but rather for stable membrane anchoring at the EHM.

This interpretation is supported by several lines of evidence: (i) both CHUP1 and KAC1 are required for MCS formation and colocalize at chloroplast–EHM contact sites, (ii) genetic disruption of either gene results in reduced callose deposition at the haustorial interface, and (iii) biochemical assays support a physical interaction between these proteins. The shared immune phenotype of *chup1* and *kac1* mutants suggests that the membrane-anchoring function mediated by these MCS is more relevant to focal immunity than chloroplast positioning alone.

It is also important to note that chloroplast movement and membrane anchoring are mechanistically distinct processes (Takagi et al., 2009). While chloroplasts can still surround haustoria in *chup1* mutants, their inability to form stable contacts with the EHM may compromise the targeted delivery or local retention of immune components, such as callose or ROS. Thus, our findings support a model where CHUP1-mediated MCS contribute specifically to immune effector deployment rather than chloroplast positioning per se.

5. Additional genetic evidence is required to establish the biological importance of the CHUP1/KAC1 complex in the context of MCS and focal immune responses. If possible, identifying and mutating key interacting domains or amino acids in CHUP1/KAC1 would provide strong supporting evidence.

Response: We appreciate the suggestions regarding further characterization of the CHUP1-KAC1 interaction interface. While we agree that such detailed mechanistic studies would be

valuable, we believe they represent a significant undertaking beyond the scope of the current manuscript:

Firstly, our computational analysis using AlphaFold3 predicts a complex, multidimensional interaction interface between CHUP1 and KAC1 (Figure A below). This complexity suggests that a comprehensive mutagenesis approach would require a systematic analysis of multiple potential interaction domains. To explore this further, we conducted multiple independent preliminary truncation experiments. Consistent with the AlphaFold3 predictions, even with extensive truncations of CHUP1 on both n-terminal side and c-terminal side, the two proteins still exhibited robust interaction *in planta* (Figure B below). These results confirm and indicate that CHUP1 and KAC1 interact extensively.

Secondly, this manuscript presents the novel discovery that CHUP1 and KAC1 play crucial roles in chloroplast-mediated immunity. Our co-immunoprecipitation data, subcellular localization studies, and genetic analyses provide strong evidence for their functional interaction in this context. It is worth noting that the functions of CHUP1 and KAC1 in anchoring chloroplasts to the plasma membrane and mediating chloroplast photorelocation have been well-established in previous genetic studies (Suetsugu et al., 2010; Oikawa et al., 2008). Our work builds upon this, establishing protein-protein interaction between CHUP1 and KAC1, as well as showing their punctate colocalization at the chloroplast-PM interface and demonstrating their novel role in pathogen defense contexts, specifically at the extrahaustorial membrane interface. Importantly, while mapping the precise interaction interface would provide additional mechanistic insights, it would not alter the conclusions and discoveries of our study.

Lastly, the detailed molecular characterization suggested here represents a natural progression of this work but would require extensive additional experiments, including: systematic domain mapping through truncation and point mutations; functional validation of these mutants *in planta*; assessment of their impact on chloroplast-EHM MCS formation; and evaluation of their contribution to focal immunity. Such comprehensive analyses would conservatively require several years of dedicated work by a postdoctoral researcher or PhD student, with associated funding requirements. We propose that our current findings establish the foundation for these future studies, which we are eager to pursue in subsequent work. The present manuscript focuses on the discovery and initial characterization of this novel immune-related roles of CHUP1 and KAC1, which we believe represents a significant advance in understanding organelle-mediated immunity, even without the detailed interaction interface mapping.

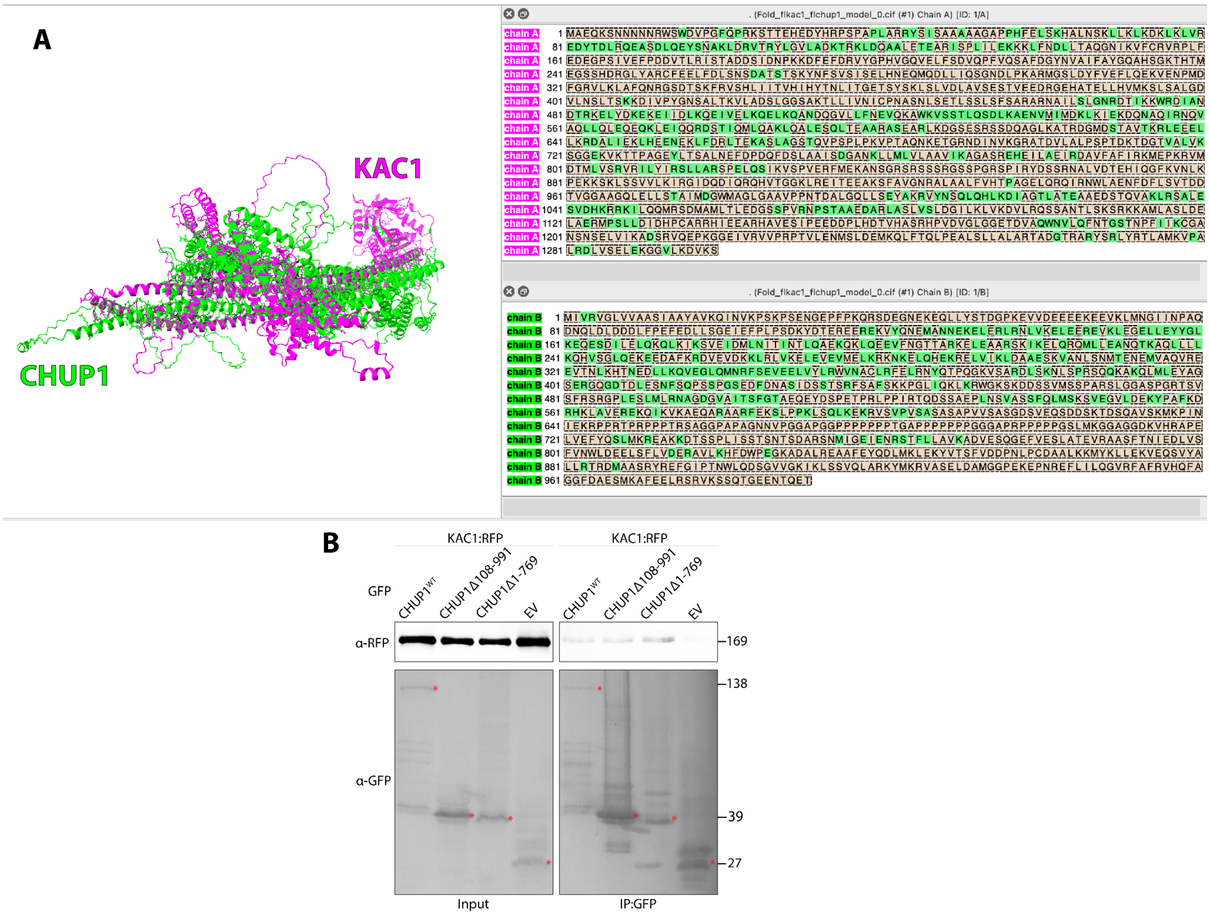

(A) AlphaFold3 predicted model of CHUP1 and KAC1 interaction. CHUP1 is depicted in lime, and KAC1 is depicted in magenta. The putative interacting residues between CHUP1 and KAC1 are predicted by ChimeraX with a stringent distance of 4 Å.

(B) KAC1 interacts with full length and the truncates CHUP1, but not with EV control. KAC1:RFP was transiently co-expressed with CHUP1(WT):GFP, CHUP1Δ108-991:GFP (truncated c-terminal), CHUP1Δ1-769:GFP (truncated n-terminal), or EV:GFP. IPs were obtained with anti-GFP antibody. Total protein extracts were immunoblotted. Red asterisks indicate expected band sizes. Numbers on the right indicate kDa values.

6. It is well-established that cortical actin is required for the focal response to fungal infection. How does the CHUP1/KAC1-mediated MCS form upon pathogen infection? Is cortical actin or cp-actin involved in this process, and if so, how? Moreover, what are the upstream signaling molecules that directly lead to the accumulation of CHUP1 at the chloroplast-PM or chloroplast-EHM interface?

Action: Our data show that CHUP1 colocalizes and interacts with KAC1 *in planta* independently of its actin-binding domain (Figures S7A and S7B), suggesting that actin-binding is dispensable for CHUP1–KAC1-mediated MCS formation.

To further investigate the role of actin, we treated *N. benthamiana* leaves with latrunculin A, which disrupts F-actin polymerization. We confirmed actin disruption using Actin:GFP as a marker (Figure S7C). Despite this disruption, CHUP1 and KAC1 continued to form MCS at

the chloroplast–PM interface (Figure S7D), indicating that F-actin is not required for their formation. However, we cannot rule out the possibility that specific cp-actin filaments contribute to the fine-tuning or stabilization of these MCS, and future studies will be needed to clarify this.

As for the upstream signals that trigger CHUP1 accumulation at chloroplast–PM or chloroplast–EHM contact sites, these remain unknown. Identifying the molecular cues that initiate this relocalization is an exciting direction for future research but is beyond the scope of the present study. Importantly, the absence of this information does not affect the conclusions drawn from our current findings.

Reviewer #3 (Comments for the Author):

The manuscript by Yuen et al. reports defence-related membrane contact sites comprising a membrane tethering complex between chloroplasts and EHM. The association between these two kinds of membranes may depend on the interaction between the chloroplast outer membrane protein CHUP1 and plasma membrane protein KAC1. The loss of function of CHUP1 or KAC1 reduces the focal deposition of callose, thereby affecting plant defence against pathogens. The concept is exciting, but the mechanism needs further study, especially the MCS and immune response results. The current data demonstrate that the interaction between CHUP1 and KAC1 is required for plant immunity, but it is speculative to interpret this phenotype as MCS-related. Further experimentation is needed to show the direct contribution of chloroplasts-EHM MCS to immune responses.

Major issues:

1. The authors suggested that MCS between chloroplast and EHM plays an essential role in plant immune response. However, it is unclear whether CHUP or KAC's loss of function affects MCS's formation. It is essential that the authors provide further data to clarify this. What happens to the Ch-EHM MCS if the interaction between CHUP1 and KAC1 is inhibited? Providing more direct evidence on MCS formation and plant immunity is necessary.

Action: We agree that establishing a clearer link between CHUP1–KAC1 interaction and MCS formation is important to support our conclusions.

In Figure 5C and Figure S6C, we showed that in *chup1* knockout plants, KAC1 fails to form puncta at chloroplast–EHM MCS, indicating that CHUP1 is required for KAC1 recruitment to these sites.

In response to the reviewer's request, we performed additional experiments to assess the effect of KAC1 silencing on CHUP1 localization. Compared to GUS-silenced control plants, KAC1 silencing caused a substantial reduction in CHUP1 punctate accumulation at both chloroplast–PM and chloroplast–EHM MCS. Specifically, CHUP1 puncta formation decreased from 82% to 13% at chloroplast–PM MCS (Figure S6), and from 73% to 23% at chloroplast–EHM MCS (Figure 5E, F). These results provide new and direct evidence that CHUP1 and KAC1 function cooperatively and are mutually required for the efficient formation of chloroplast–EHM MCS. This coordinated activity supports their proposed role in promoting focal immune responses during pathogen infection.

2. In addition, is the immunity phenotype in the chup1 mutant related to EHM-chloroplast MCS formation? We know chloroplasts are implicated in the immune response. Are we expecting an immunity-related phenotype when chloroplast functions are affected in general? Does the chup1 mutant exhibit a general defect in chloroplast function? In other words, the causal relationship between the chup1 phenotype and MCS establishment is unclear. It is not convincing to say "chloroplast-EHM MCS positively regulates plant focal immunity."

Action: In our manuscript, we stated, "Our findings suggest that the chloroplast-EHM attachment complex positively regulates plant focal immunity," carefully framing our conclusion as a suggestion rather than a definitive statement. We acknowledge that conclusively demonstrating its regulatory role in focal immunity would require further investigation and we added additional sentences in the discussion that this is not definitive and needs further research to be conclusive.

Our observations indicate that the general immune functions of the chloroplast are not significantly affected in the *chup1* mutant. As shown in the manuscript, the loss of *chup1* does not impair PTI or ETI responses (Figure S3) nor affect chloroplast movement around haustoria, chloroplast numbers, or total chlorophyll levels (Figure S1). Our results suggest that the chloroplast-EHM MCS involves two independent genes, CHUP1 and KAC1. The fact that knocking out/silencing either gene results in the same immunity-related phenotype strongly supports the role of these MCS in immune responses. Additionally, both proteins localize to the EHM MCS during pathogen infection, reinforcing the significance of these contact sites in immunity. The punctate accumulation of KAC1, a plasma membrane protein, at chloroplast MCS further supports their involvement in immune functions.

We acknowledge that while the precise mechanism by which these MCS influence focal immunity remains unclear, the shared immunity phenotype observed in *chup1* and *kac1* mutants provides compelling evidence that these contact sites contribute to plant immune responses.

3. The authors show that the loss of function of CHUP or KAC affects the accumulation of callose around the haustoria, but the underlying mechanism is unclear. Further data are necessary to show how these two proteins are responsible for this phenotype.

Response: We agree that uncovering the precise molecular mechanism by which CHUP1 and KAC1 influence callose accumulation at the haustorial interface would provide valuable insights. However, dissecting this mechanism involves substantial technical and conceptual challenges, including identifying downstream effectors and transport pathways, which are beyond the scope of the current study.

Our current data establish a strong correlation between the loss of CHUP1 or KAC1 function, impaired chloroplast–EHM MCS formation, and reduced focal callose deposition. These findings suggest that CHUP1–KAC1-mediated MCS may facilitate the targeted delivery or localized retention of immune components, such as callose precursors or ROS, at the site of pathogen attack. While further mechanistic work is certainly warranted, the conclusions of this study remain well supported by the genetic, cell biological, and biochemical evidence presented. We aim to explore the downstream molecular mechanisms in future research.

4. Most subcellular structures are recruited to the EHM region after infection in tobacco leaf epidermal cells. So, is the CHUP1-KAC1 regulated pathway specific to chloroplast-EHM MCS? Could it affect the enrichment of other membrane or cytoskeletal structures at the EHM?

Response: To our knowledge, CHUP1 and KAC1 have not been shown to influence membrane or cytoskeletal structures beyond their roles in chloroplast positioning and anchoring. In our study, we focused on their function in forming chloroplast–EHM membrane contact sites during infection. Whether the CHUP1–KAC1 pathway also affects the recruitment or organization of other membrane or cytoskeletal components at the EHM remains unknown. This is an intriguing possibility and represents an exciting direction for future investigation, though it lies beyond the scope of the present study.

5. Since this is likely the first time Ch-EHM MCS has been discovered, I think the authors should better define this type of MCS. Some additional TEM studies will be helpful, as the resolution of light microscopy is insufficient to resolve MCS.

In response to the requests, we have employed both cryogenic electron tomography (CryoET) and transmission electron microscopy (TEM) with immunogold labeling to investigate membrane contact sites (MCS) between the chloroplast and the plasma membrane (PM) and to assess the localization of CHUP1.

CryoET: Data from cryoET revealed clear contact points between the chloroplast outer membrane and plasma membrane. These take the form of membrane protrusions linked by a layer of ~30 nm long and potentially highly flexible protein. The thickness of this protein layer is consistent with the predicted size of the putative CHUP1:KAC1 complex. The high quality of the data is apparent, as we have been able to resolve RuBisCo complexes in the chloroplast stroma and even individual cellulose fibres in the cell wall.

Importantly, to obtain these data we had to develop a new and highly technically challenging workflow for cryogenic imaging of plant tissues (protocol described in Methods). This would be the first published example of a similar approach used to study plant cells.

Immunogold TEM labeling: We performed immunogold labeling using anti-GFP antibodies to localize CHUP1:GFP. However, the results were inconclusive due to non-specific labeling of both CHUP1:GFP and EV:GFP. Although we observed instances where gold particles labeled CHUP1:GFP at the chloroplast-PM MCS, these findings are not definitively conclusive, as we also observed widespread non-specific immunogold labeling across the chloroplast in both experimental and control samples (see figure below). This is consistent with the known tendency of immunogold labelling for non-specific binding, especially with specimens containing plastids. Given these limitations, TEM-based immunogold labeling is not a reliable method for confirming CHUP1 localization at MCS in our system. Reassuringly, in the immunogold TEM data (which was prepared through high pressure freezing and freeze-substitution), we have notice membrane protrusions with those observed in cryoET.

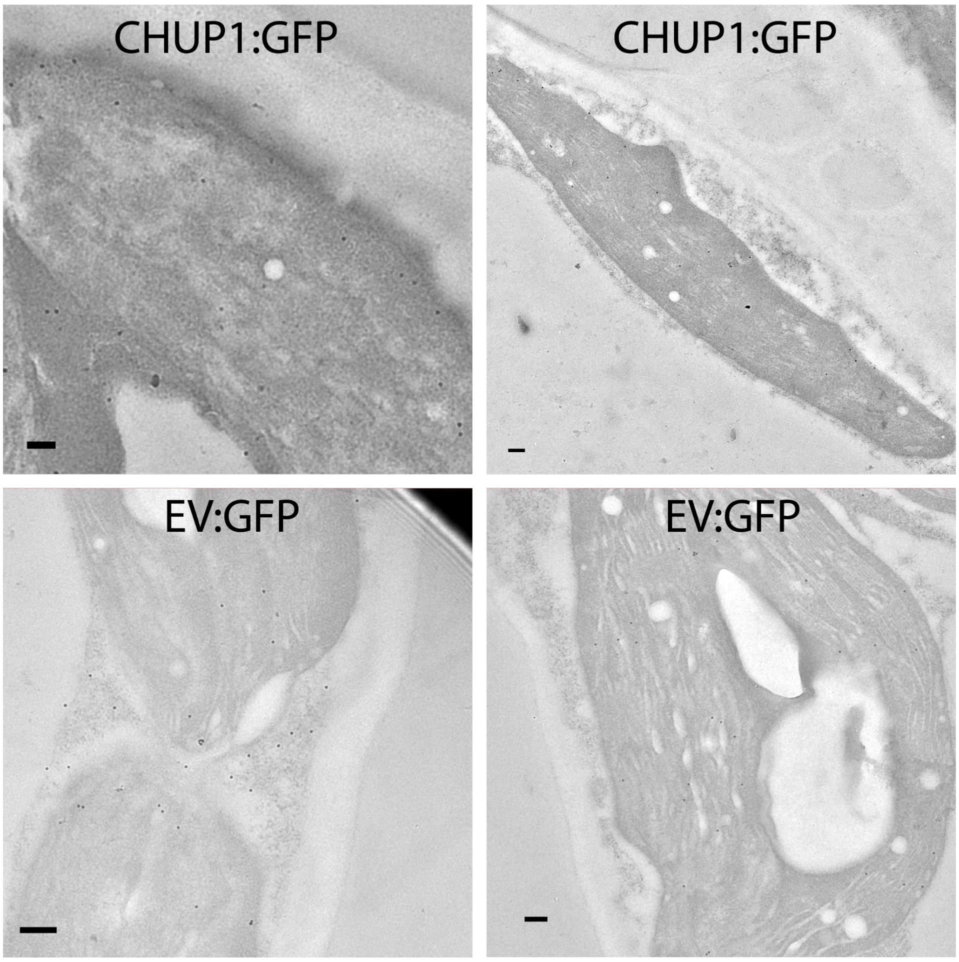

This figure shows immunogold labelling of CHUP1:GFP and control EV:GFP samples. Scale bar represents 200 nm. Specimens have been prepared by high pressure freezing and freeze-substitution.

Together, these complementary high-resolution imaging approaches provide strong support for the presence of MCS between the chloroplast and the PM. However, due to the limitations of immunogold TEM, we cannot conclusively determine the localization of CHUP1 at these sites. Future studies will be needed to investigate the precise localization and the structure of CHUP1 at chloroplast-PM contact sites.

6. Figure 4: It is necessary to use more than one RNAi line to study the phenotype.

Response: In response to the reviewer's request, we have generated an additional independent RNAi construct (RNAi:KAC#2) targeting regions of KAC1 and KAC2 distinct from those targeted by the RNAi:KAC#1 construct. We verified the functionality of the RNAi:KAC#2 construct through RT-PCR analysis and conducted *P. infestans* infection assays using plants expressing RNAi:KAC#2. The results demonstrate a significant increase in pathogen hyphal growth in plants expressing RNAi:KAC#2 compared to control silencing plants, further confirming that KACs contribute to plant immunity against *P. infestans*.

7. Figure 5: Please use multiple methods to confirm the interaction between CHUP and KAC.

Response: In our study, we employed co-IP, a well-established method for detecting interactions under native conditions. Given that CHUP1 and KAC1 interact at membrane contact sites, approaches such as yeast two-hybrid, which rely on nuclear localization, may not be suitable for confirming membrane-associated interactions.

To support the robustness of our co-IP results, we included multiple controls. For example, Toc64, another chloroplast outer membrane protein, was used as a control, while the N-terminal region of CHUP1, which is required for its localization to the chloroplast outer envelope, served as a negative control. Additionally, our confocal imaging data show specific co-localization of CHUP1 and KAC1 in punctate structures at chloroplast–PM and chloroplast–EHM contact sites, providing spatial context to support the biochemical interaction.

Our findings are also consistent with prior literature showing that CHUP1 and KAC1 function together in cp-actin-mediated chloroplast positioning (Suetsugu et al., 2012), further supporting their interaction. Moreover, the same co-IP and localization methodologies have been successfully applied in our previous studies (e.g., Duggan et al., 2021; Pandey et al., 2018), demonstrating their reliability in detecting membrane-associated protein complexes.

We believe that the combined biochemical and imaging data presented here provide strong and physiologically relevant evidence for the CHUP1–KAC1 interaction.

Minor issues:

1. Figure S1K shows the chloroplast distribution in FNR and chup1 mutants. It seems that these two images show different layers of the cell. Please also label the cell margin.

Action: The phenotype of *chup1* KO plants shows chloroplasts clustering around the nucleus (Figure S1I). Therefore, the clustering difference observed in Figure S1K is due to chloroplast clustering around the nucleus for *chup1* KO plants, compared to the control FNR plants.

The confocal images presented are Z-stack projections, making it impractical to label single-plane cell margins. In response to the reviewer's request, we have included 3D projection movies of these confocal images as Supplemental Movies 1 and 2.

2. Figure S4, the expression level of housekeeping gene GAPDH is different on the left panel. Please ensure the amount of the cDNA template used for each reaction is identical.

Action: We have repeated the RT-PCR, and the housekeeping gene GAPDH now shows a consistent cDNA expression level.

**Date:**	May 14, 2025
**To:**	"Tolga Osman Bozkurt" o.bozkurt@imperial.ac.uk
**From:**	"The Plant Cell" tpc-submissions@aspb.org
**Subject:**	TPC-2025-0380 Decision Letter

Ref.: Ms. No. TPC-2025-0380
Membrane Contact Sites Between Chloroplasts and Pathogen Interface Underpin Plant Focal Immune Responses
The Plant Cell

Dear Tolga:

We have received reviews of the manuscript above. On the basis of the advice received, the board of reviewing editors would like to accept your manuscript for publication in The Plant Cell. This acceptance is contingent on revision based on the comments of our reviewers. In particular, all three reviewers suggested further confirmation on CHUP1-KAC1 protein-protein interaction using an independent method of co-IP.

Please highlight all changes and include a detailed annotation of changes of the text, with line numbers, and noting your responses to the comments. A link to submit your revision is below the reviewer comments.

If you have any questions about the revision submission procedures, please contact the Editorial Office Staff (tpc-submissions@aspb.org). If you cannot return the revised manuscript within 30 days, please let us know. Otherwise, we will assume that you have elected not to revise the manuscript and withdraw it.

Thank you very much for the privilege of reviewing this work. I look forward to receiving the next version.

On behalf of the editorial board,

Wenbo Ma, Senior Editor
Pablo Manavella, Editor-in-Chief
The Plant Cell

Reviewers' comments:

Reviewer 1: The authors have addressed most of my concerns; however, I still believe the interaction between CHUP1 and KAC1 requires further confirmation. According to the methods section, Co-IP was performed using transiently transformed N. benthamiana leaves. This indicates that two proteins can interact without infection, raising the question of where this interaction occurs and whether this interaction is directly related to the EHM-Chloroplast MCS. It would be more convincing to have the localisation information of such an interaction, using Bifc (with careful controls) or FRET, or other recently established methods (e.g., Split-Fast). Additionally, I feel it is also necessary to show the localization of CHUP1 and KAC1 in non-infected cells (similar to Figure 2, with low and high magnifications).

Reviewer 2: The revised manuscript addresses several earlier concerns and shows clear improvement in clarity and depth. I acknowledge the authors' efforts to investigate the CHUP1-KAC1 interaction interface. It is clear that considerable work was undertaken, and the negative results from the truncation analyses are informative in themselves. That said, I continue to hold the view that elucidating the mechanism by which the CHUP1/KAC1 complex forms would provide direct evidence for its role in mediating MCSs during focal immune responses, beyond the functional association currently demonstrated. I fully understand that such mechanistic dissection is challenging and likely beyond the scope of the present study. The current version of the manuscript presents relevant and novel findings that warrant publication. Nonetheless, I encourage future efforts to further strengthen the importance of the EHM-chloroplast MCS in focal immunity.

Reviewer 3: The revised manuscript presents a compelling and well-structured body of work. The new microscopy data are convincing and add substantial value to the field. However, I remain concerned regarding the validation of the CHUP1-KAC1 protein-protein interaction, which constitutes a central pillar of the study. The rest of the questions/comments were satisfactorily answered.

While co-immunoprecipitation (co-IP) under native conditions is indeed a strong method and is well controlled in the present manuscript, I believe that additional evidence is required to support a direct physical interaction, especially given the centrality of this interaction to the authors' model.
The authors argue that methods such as yeast two-hybrid (Y2H) are not appropriate due to subcellular localization constraints. However, Y2H remains a widely used and accepted method for validating protein-protein interactions, including those involving membrane-associated proteins, provided appropriate design considerations are made. Therefore, the rationale provided is, in my opinion, not sufficient to dismiss this or other orthogonal approaches altogether.
Furthermore, based on previous publications and the apparent resources available to the authors, I believe it would be both feasible and appropriate to include at least one additional method to confirm the interaction (e.g., pull-down, FRET-FLIM, or Y2H). This would considerably strengthen the manuscript and ensure that the conclusions drawn are fully supported.

In summary, while the manuscript is otherwise strong and of interest, I encourage the authors to provide further experimental validation of the CHUP1-KAC1 interaction using a method independent of co-IP.

****

Please also note the following:

-The Plant Cell now requires authors to complete and submit an author revisions checklist upon submission of a revised manuscript. The aim of the checklist is to aid authors in preparing a high-quality manuscript, facilitate the review and assessment of revised manuscripts, and help to ensure that journal standards are maintained across the board. If your manuscript is accepted, the completed checklist will be published as supplemental material attached to the article online. Please download a copy of the checklist (pdf fillable form) at this link, for submission with your revised manuscript: https://tpc.msubmit.net/html/Author_Revisions_Checklist.pdf.

-Supplemental materials should be restricted to large datasets and tables, presentation of replicates, and validation of reagents, methods, or genotypes. Any data that are used to support the major claims must be in the main manuscript. Supplemental figure legends must indicate what figure in the main manuscript is supported by the supplemental data presented. Please justify how each of the supplemental figures meet the criteria.

-Sampling methods and the nature of "biological replicates" should be described precisely (i.e. different plants, parts of plants, pooled tissue, independent pools of tissue, sampled at different times, etc.), along with a clear description of and rationale for any statistical analyses conducted. The reader should know exactly what was sampled; what forms the basis of the calculation of any means and statistical parameters reported. This is also necessary to ensure that proper statistical analysis was conducted.

In compliance with data protection regulations, you may request that we remove your personal registration details at any time. (Use the following URL: https://www.editorialmanager.com/tpc/login.asp?a=r). Please contact the publication office if you have any questions.

Author's Response To Reviewer Comments

Close

Dear Prof. Ma and Prof. Manavella,

We sincerely appreciate the time and expertise that the editors and reviewers have dedicated to evaluating our manuscript. We are especially grateful for the tentative acceptance of our study at The Plant Cell. The reviewers' constructive and insightful feedback has significantly enhanced the clarity and experimental depth of our work. Below, we provide a unified response, detailed point-by-point replies to each reviewer, and a complete annotation of the changes made to the manuscript.

United response

In this final revision, we have incorporated two key datasets that directly address the remaining concerns and provide strong, independent validation of our main conclusions:
1. Fluorescence-lifetime imaging microscopy–Förster resonance energy transfer (FLIM-FRET) demonstrating in planta molecular interaction between CHUP1 and KAC1 (Figure S11).
2. Immunogold labeling transmission electron microscopy (TEM) revealing strong enrichment of CHUP1 at chloroplast–plasma membrane (PM) contact sites (Figures 5J–L).

The FLIM-FRET data show that co-expression of CHUP1:GFP and KAC1:RFP results in a 143 ps reduction in donor fluorescence lifetime compared to CHUP1:GFP-only controls. This corresponds to approximately 60% of the maximal FRET signal observed in a covalent GFP-RFP fusion and suggests that CHUP1 and KAC1 are within ~10 nm of each other in planta. These data, together with our co-immunoprecipitation results, provide convergent biochemical and biophysical evidence that CHUP1 and KAC1 form a complex at chloroplast–PM membrane contact sites.

The immunogold TEM analysis supports the spatial localization of CHUP1 at these sites. In CHUP1:GFP-expressing samples prepared by high-pressure freezing and freeze substitution, gold particles recognizing GFP were consistently enriched at chloroplast–PM MCS. In contrast, EV:GFP controls exhibited a more random chloroplast distribution. Quantitative assessment confirmed a significantly higher MCS-to-chloroplast labeling ratio in CHUP1:GFP samples, reinforcing the functional relevance of CHUP1 localization to these contact sites.

Response to each reviewer

Reviewer 1

Comments:
"The authors have addressed most of my concerns; however, I still believe the interaction between CHUP1 and KAC1 requires further confirmation. According to the methods section, Co-IP was performed using transiently transformed N. benthamiana leaves. This indicates that two proteins can interact without infection, raising the question of where this interaction occurs and whether this interaction is directly related to the EHM-Chloroplast MCS. It would be more convincing to have the localisation information of such an interaction, using Bifc (with careful controls) or FRET, or other recently established methods (e.g., Split-Fast). Additionally, I feel it is also necessary to show the localization of CHUP1 and KAC1 in non-infected cells (similar to Figure 2, with low and high magnifications)."

Response:
We thank the reviewer for raising this important point. In response, we have now conducted Fluorescence-lifetime imaging microscopy–Förster resonance energy transfer (FLIM-FRET). The FLIM-FRET data show that co-expression of CHUP1:GFP and KAC1:RFP results in a 143 ps reduction in donor fluorescence lifetime compared to CHUP1:GFP-only controls. This corresponds to approximately 60% of the maximal FRET signal observed in a covalent GFP-RFP fusion and suggests that CHUP1 and KAC1 are within ~10 nm of each other in planta. These data, together with our co-immunoprecipitation results, provide convergent biochemical and biophysical evidence that CHUP1 and KAC1 form a complex at chloroplast–PM membrane contact sites.

We also agree that spatial context is crucial. To address this, we performed immunogold transmission electron microscopy (Figures 5J–L). The immunogold TEM analysis supports the spatial localization of CHUP1 at the chloroplast-PM MCS. In CHUP1:GFP-expressing samples prepared by high-pressure freezing and freeze substitution, gold particles recognizing GFP were consistently enriched at chloroplast–PM MCS. In contrast, EV:GFP controls exhibited a more random chloroplast distribution. Quantitative assessment confirmed a significantly higher MCS-to-chloroplast labeling

ratio in CHUP1:GFP samples, reinforcing the functional relevance of CHUP1 localization to these contact sites.

Finally, regarding the request for CHUP1 and KAC1 localization in non-infected cells, we note that these data are already included in Figure S5.

Reviewer 2

Comments:
"The revised manuscript addresses several earlier concerns and shows clear improvement in clarity and depth. I acknowledge the authors' efforts to investigate the CHUP1-KAC1 interaction interface. It is clear that considerable work was undertaken, and the negative results from the truncation analyses are informative in themselves. That said, I continue to hold the view that elucidating the mechanism by which the CHUP1/KAC1 complex forms would provide direct evidence for its role in mediating MCSs during focal immune responses, beyond the functional association currently demonstrated. I fully understand that such mechanistic dissection is challenging and likely beyond the scope of the present study. The current version of the manuscript presents relevant and novel findings that warrant publication. Nonetheless, I encourage future efforts to further strengthen the importance of the EHM-chloroplast MCS in focal immunity."

Response:
We are grateful for the reviewer's thoughtful evaluation and their recognition of the value and novelty of our findings. We fully agree that defining the exact mechanism of CHUP1-KAC1 complex formation is an important future direction, and we appreciate the understanding that such dissection may be beyond the current scope. To strengthen our current conclusions, we have added FLIM-FRET analysis that provides biophysical evidence for a direct interaction between CHUP1 and KAC1 in planta. We believe these new results significantly advance our understanding of the CHUP1/KAC1 complex interaction.

Reviewer 3

Comments:
"The revised manuscript presents a compelling and well-structured body of work. The new microscopy data are convincing and add substantial value to the field. However, I remain concerned regarding the validation of the CHUP1-KAC1 protein-protein interaction, which constitutes a central pillar of the study. The rest of the questions/comments were satisfactorily answered. While co-immunoprecipitation (co-IP) under native conditions is indeed a strong method and is well controlled in the present manuscript, I believe that additional evidence is required to support a direct physical interaction, especially given the centrality of this interaction to the authors' model. The authors argue that methods such as yeast two-hybrid (Y2H) are not appropriate due to subcellular localization constraints. However, Y2H remains a widely used and accepted method for validating protein-protein interactions, including those involving membrane-associated proteins, provided appropriate design considerations are made. Therefore, the rationale provided is, in my opinion, not sufficient to dismiss this or other orthogonal approaches altogether. Furthermore, based on previous publications and the apparent resources available to the authors, I believe it would be both feasible and appropriate to include at least one additional method to confirm the interaction (e.g., pull-down, FRET-FLIM, or Y2H). This would considerably strengthen the manuscript and ensure that the conclusions drawn are fully supported. In summary, while the manuscript is otherwise strong and of interest, I encourage the authors to provide further experimental validation of the CHUP1-KAC1 interaction using a method independent of co-IP."

Response:
We thank the reviewer for their positive comments and for highlighting the central importance of validating the CHUP1-KAC1 interaction. In response, we have performed FLIM-FRET analysis. The FLIM-FRET data show that co-expression of CHUP1:GFP and KAC1:RFP results in a 143 ps reduction in donor fluorescence lifetime compared to CHUP1:GFP-only controls. This corresponds to approximately 60% of the maximal FRET signal observed in a covalent GFP-RFP fusion and suggests that CHUP1 and KAC1 are within ~10 nm of each other in planta. These data, together with our co-immunoprecipitation results, provide convergent biochemical and biophysical evidence that CHUP1 and KAC1 form a complex at chloroplast–PM membrane contact sites.

Complete annotation of changes to the text (highlighted in red in the manuscript file)
- Added three new co-authors and their respective affiliations.
- Revised main text describing cryo-electron tomography (cryoET) (Lines 400–408).
- Introduced new main text detailing the immunogold labeling experiments (Lines 408–414).
- Added a new section to the Materials and Methods detailing the immunogold labeling methodology (Lines 775–788).
- Included Figures 5J, 5K, and 5L illustrating immunogold labeling results (Lines 494–504).
- Incorporated main text describing the non-fitting FLIM-FRET analysis (Lines 426–440).
- Added a new section to the Materials and Methods detailing the non-fitting FLIM-FRET methodology (Lines 713–725).
- Added Figure S11 relating to the non-fitting FLIM-FRET data (Lines 1390–1403).
- Updated the Acknowledgements and Funding sections to include additional contributors and sources of support (Lines 911–926).

Conclusion
We believe these additional experiments and comprehensive revisions fully resolve all outstanding concerns and substantially strengthen the manuscript. Thank you again for your thoughtful feedback and support throughout the

review process. We look forward to your final decision on our revised submission.

Sincerely,
Dr. Tolga Bozkurt

Close

Date: Jul 26, 2025
To: "Tolga Osman Bozkurt" o.bozkurt@imperial.ac.uk

From: "The Plant Cell" tpc-submissions@aspb.org
Subject: TPC-2025-0380R1 Decision Letter

Ref.: Ms. No. TPC-2025-0380R1
Membrane Contact Sites Between Chloroplasts and Pathogen Interface Underpin Plant Focal Immune Responses
The Plant Cell

Dear Tolga,

I am very pleased to inform you that your revised manuscript "Membrane Contact Sites Between Chloroplasts and Pathogen Interface Underpin Plant Focal Immune Responses" has been accepted for publication in The Plant Cell. Congratulations on this beautiful paper!

At this stage, your manuscript will be sent for additional pre-publication science editing if it is one of the following article types: Research Article, Large-Scale Biology, Breakthrough Report. The Science Editors will review the manuscript with respect to its presentation of scientific content, compliance with journal policies, and presentation for a broad readership. The Plant Cell has contracted with Plant Editors (planteditors.com) to provide this service to our authors, and you will soon receive additional information on this process.

ORCID ID NEEDED: Please note that each author needs to link their ORCID identifier to their account in the system before your manuscript can be published. If any authors do not have an ORCID linked to their account, they will receive a message with a link to complete this task. Please ensure that ALL of your coauthors have completed this task as soon as possible.

LICENSE INFORMATION Shortly after your paper is sent to production, you will receive an email from our publisher's author support team, SciPris, with information on article processing charges, as well as the types of Open Access license you would be able to purchase. The accepted version of your manuscript will not appear on our Advance Articles page until the license is selected and signed, so please look out for an email from SciPris. For more information see the Article Publication Fees section of our author guidelines: https://academic.oup.com/plcell/pages/General-Instructions

The Plant Cell and The Arabidopsis Information Resource (TAIR) are collaborating to collect functional annotation data about Arabidopsis genes from authors. This includes information about the gene's molecular function (e.g., kinase activity, ATP synthetase activity), the biological process/es it is involved in (e.g., endosperm development, threonine biosynthesis), its subcellular location (e.g., nucleus, ER), anatomical or developmental expression pattern (e.g., leaf, ovule, flower stage 10, seedling stage), or its partner in a protein-protein interaction (e.g., AT1G01010 interacts with AT1G01020).

If your paper contains results falling into one or more of these categories for Arabidopsis genes, we request that you now submit these data for inclusion in TAIR by filling in the form provided at the following URL: https://www.arabidopsis.org/doc/submit/functional_annotation/123. If you need further clarification on what types of data can be submitted please contact curator@arabidopsis.org.

Finally, we encourage your submission of artwork for the journal cover. If there is an image or illustration related to your paper that you would like to have considered, please email it in .pdf or .tif format to clowe@aspb.org. An image title, brief, 2-3 sentence description of the image, and the name of the person credited for the image are also encouraged. If your image is selected to appear on the online cover of the journal, you will be contacted with the scheduled issue date and further information.

We look forward to seeing your paper published.

On behalf of the editorial board,

Sincerely,

Wenbo Ma, Senior Editor

Pablo Manavella, Editor-in-Chief
The Plant Cell

=====
IMPORTANT REMINDER: PEER REVIEW REPORTS
=====

If you opted to publish a peer review report along with your article during the original submission process, it will be prepared by the editorial staff and publicly posted with your manuscript, inside the zip file that contains any other supplemental material. As a reminder, the peer review report is a public record of all comments from editors and reviewers, as well as your prior responses, as you received them in the decision letters for each draft of your manuscript. If you agreed to publish this report and have changed your mind, or are not sure if you selected this option, please contact the editorial office as soon as possible before signing the license agreement from our publisher.

Comments from the Editors and Reviewers:

Reviewers' comments:

****

Ref.: Ms. No. TPC-2025-0380R1
Membrane Contact Sites Between Chloroplasts and Pathogen Interface Underpin Plant Focal Immune Responses
The Plant Cell

In compliance with data protection regulations, you may request that we remove your personal registration details at any time. (Use the following URL: https://www.editorialmanager.com/tpc/login.asp?a=r). Please contact the publication office if you have any questions.
